# Design, synthesis, and evaluation of chiral thiophosphorus acids as organocatalysts

**DOI:** 10.3762/bjoc.18.154

**Published:** 2022-10-17

**Authors:** Karen R Winters, Jean-Luc Montchamp

**Affiliations:** 1 Department of Chemistry and Biochemistry, Texas Christian University, Fort Worth, Texas 76129, United States

**Keywords:** asymmetric, heterocycles, organocatalysis, phosphorus, synthesis

## Abstract

A series of *P*-stereogenic chiral phosphorus acids (CPAs) were synthesized to determine the requirements for efficient asymmetric organocatalysis. In order to eliminate the need for *C*_2_-symmetry in common CPAs, various scaffolds containing *C*_1_-symmetrical thiophosphorus acids were chosen. These new compounds were synthesized and evaluated in the asymmetric transfer hydrogenation of 2-phenylquinoline. Although the efficacy of the thiophosphorus acids was disappointing for this reaction, the work should be useful for developing structural design elements.

## Introduction

**Figure 1:**
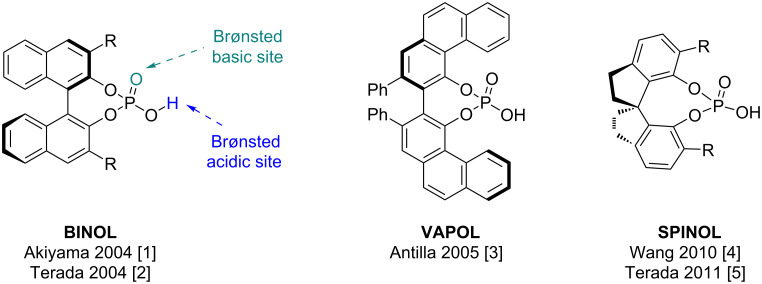
Chiral phosphorus acids (CPAs) derived from BINOL, VAPOL, and SPINOL. R = H, Ph, 4-PhC_6_H_4_-, 4-β-naphthylphenyl, 9-anthryl, 3,5-dimesitylphenyl, 3,5-diphenylphenyl, 4-MeC_6_H_4_-, 4-CF_3_C_6_H_4_-, 4-*t*-BuC_6_H_4_-, β-naphthyl, 3,5-*t*-Bu_2_C_6_H_3_-, 2,4,6-Me_3_C_6_H_2_-, 2,4,6-iPr_3_C_6_H_2_-, Ph_3_Si-, etc.

The importance of asymmetric organocatalysis was demonstrated by the 2021 Nobel Prize in Chemistry awarded to McMillan and List. A subclass of organocatalysts introduced independently by Akiyama and Terada in 2004 [1–2], are the *C*_2_-symmetrical chiral phosphorus acids (CPAs) initially derived from the BINOL scaffold, and later extended to other scaffolds such as VAPOL [3] and SPINOL [4–5] (Figure 1). The great success of these CPAs in asymmetric organocatalysis, is demonstrated by the publication of thousands of articles and reviews [6–17]. In all cases the *C*_2_-symmetry is required because of the prototropic tautomeric equilibrium in the hydroxyphosphoryl (P(=O)OH) moiety which renders the phosphorus atom achiral. Substituents can be introduced on the ring system by ortho-functionalization with R groups on each ring. This functionalization helps introduce steric bulk and a range of electron densities extending the *C*_2_-symmetry of the BINOL, creating a chiral pocket or environment for enantioselective transformations within the proximity of the acidic proton and phosphoryl oxygen. Additionally, the choice of phosphoric acid diesters also provides a bifunctional catalyst containing both an acidic and basic site (Figure 1).

Despite the proven value of the CPAs described in the literature, several disadvantages can be identified [18]. As mentioned above, *C*_2_-symmetry is required for the catalysts to provide a chiral pocket around the phosphorus. As a result, the CPAs have very high molecular weights (>> 450 g/mol) and require a wasteful duplicative functionalization of the backbone. Moreover, commercially available CPAs are extremely expensive (>> 500,000 $/mol) and immobilizing the CPAs on a solid support is not straightforward [19–20]. In order to avoid this, a significant investment in time must be made to complete the multistep-syntheses that are required [1–4]. Additionally, whereas either enantiomer of BINOL is relatively inexpensive (109 $/mol), it is not the case with SPINOL (17,000 $/mol), and VAPOL is not commercially available. Although one could synthesize these precursors as well, this multistep synthesis is time-consuming and costly. For example, the resolution of racemic SPINOL uses 2.4 equivalents of menthyl chloroformate [21] which itself costs 1,000 $/mol. Furthermore, the R group often needs to be optimized to obtain good enantioselectivities and there does not seem to be a universally successful CPA at this time. Consequently, the availability of each CPA enantiomer requires significant synthetic efforts from the diphenol precursor.

**Scheme 1:**
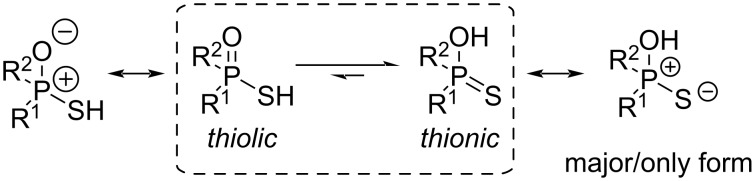
The thiolic/thionic tautomeric equilibrium in thiophosphorus acids.

In order to address these issues, we became interested in exploring *C*_1_-symmetrical CPAs, in which the chirality resides exclusively at the phosphorus atom. For this exploratory work, thiophosphorus acids were chosen due to their appropriate acidity and intrinsic chirality. Thiophosphorus acids undergo a tautomeric equilibrium between the thiolic and the thionic forms [22] (Scheme 1). If the substituents R^1^ and R^2^ are different, the phosphorus atom is always chiral. Chiral thiophosphorus acids have been obtained by resolution with a chiral amine as early as 1958 [23–27], or from other precursors [28–30].

**Figure 2:**
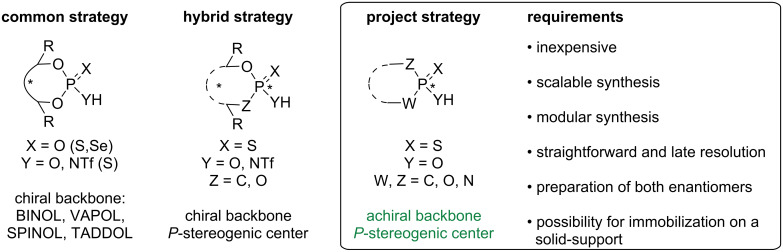
Project strategy and requirements for *C*_1_-symmetrical CPAs.

Having selected chiral thiophosphorus acids for our model study, further design requirements were included (Figure 2) to address some issues listed above for the *C*_2_-symmetrical catalysts. First and foremost, the compounds must be inexpensive to make, which implies that their syntheses should be easily scaled. A modular synthesis is also desirable if some structure optimization is required. The resolution of the phosphorus center should be straightforward and accomplished late-stage, to avoid carrying the chirality through multiple steps and the possible erosion of enantiomeric excesses. Preferably, both enantiomers of the CPA should also be available and immobilization of the CPA on a solid support should be possible. In this paper, we report our progress towards these objectives.

It should be noted that a few examples of a "hybrid strategy" in which both the backbone and the phosphorus atom are chiral have been reported by Guinchard [31] and Murai [32].

## Results and Discussion

### CPA Design

**Figure 3:**
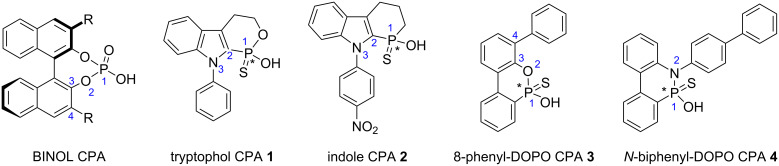
BINOL CPA and *C*_1_-symmetrical CPA targets **1**–**4**.

At the outset, we were interested in probing the geometry and influence of the substituent position in the CPAs (Figure 3). In the BINOL-derived CPA, the R-substituent and the phosphorus atom are separated by three bonds. In the indole-based CPAs **1** and **2**, the distance is reduced to two bonds, whereas in CPA **3** it is three bonds, and in **4** it is just one bond. Both **3** and **4** are based on 9,10-dihydro-9-oxa-10-phosphaphenanthrene-10-oxide (DOPO) [33].

### Synthesis

In this section, the syntheses of CPA targets are described. It should be noted that little yield optimization was accomplished since only a small amount of product was needed for the evaluation as an enantioselective catalyst. On the other hand, their successful completions attest to the inexpensive and scalable requirements we had set.

#### Indole scaffolds

**Scheme 2:**
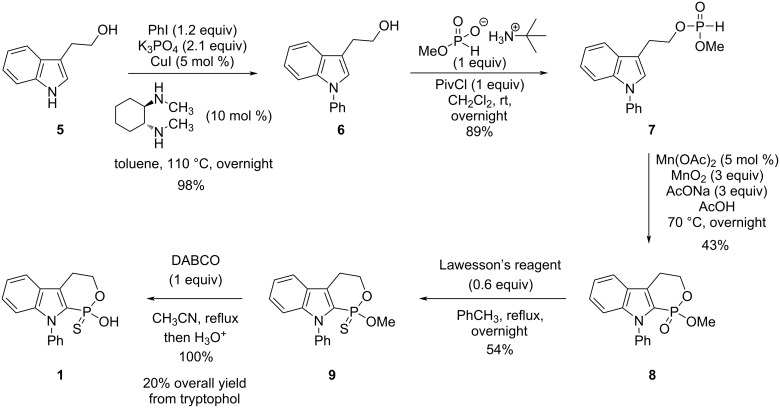
Synthesis of tryptophol-derived thiophosphorus acid **1**.

The synthesis of racemic tryptophol CPA **1** is shown in Scheme 2. Commercially available tryptophol (**5**, 225 $/mol) was N-arylated into **6** via copper-catalyzed cross-coupling [34] in excellent yield. Esterification of **6** with monomethyl *H*-phosphonate *tert*-butylamine salt [35] resulted in the mixed *H*-phosphonate ester **7** in excellent yield.

**Scheme 3:**
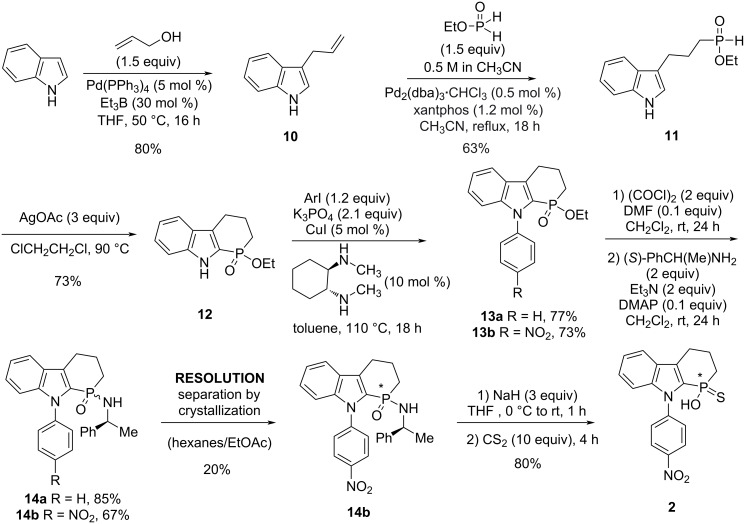
Synthesis of indole-derived thiophosphorus acid **2**.

Cyclization using our homolytic aromatic substitution methodology [36] gave *P*-heterocycle **8** in modest yield. Other methods based on silver either gave a complex mixture or unreacted starting material. Phosphonate **8** was converted into the corresponding thiophosphonate **9** in moderate yield using Lawesson's reagent. Cleavage of the methyl ester was easily accomplished in quantitative yield, producing racemic tryptophol CPA **1**. The resolution of compound **1** was not conducted at this point because its synthesis was deemed problematic. While relatively short (5 steps), the overall yield was only 20% due to a low-yielding key step and a problematic thionation step immediately following. Unfortunately, thionation of **8** with an alternative [37] to Lawesson's reagent did not solve the problem. This prompted our search for alternative methodologies for the synthesis of thiophosphorus acids [38], particularly using the Stec reaction [39–40]. This work also led to the synthesis of CPA **3** [38]. Alternatives to the Stec reaction to prepare chiral thiophosphorus acids have been described [41–43]. Once equipped with our new methods [38], the synthesis of indole-derived **2** was undertaken (Scheme 3).

Known 3-allylindole (**10**) [44] was obtained from indole uneventfully. Intermediate **11** was furnished in moderate yield via our palladium-catalyzed hydrophosphinylation [45]. The key heterocyclization of **11** into **12** was accomplished using silver-promoted homolytic aromatic substitution [46], which was superior to our own manganese methodology (43% yield) [36]. Copper-catalyzed arylation [34] of **12** with iodobenzene and 4-nitroiodobenzene gave intermediates **13a** and **13b**, respectively. Next, conversion of ethyl phosphinate **13** into phosphinamide **14** was accomplished uneventfully [38] with inexpensive (*S*)-1-phenylethylamine (15 $/mol) as the chiral element. A single diastereoisomer of phosphinamide **14b** was easily obtained by crystallization in 20% yield. Subsequent Stec reaction [38–40] gave chiral CPA **2** stereospecifically with retention of configuration [39]. This synthesis accomplishes a few of the requirements that were set inititally (see Figure 2). The chemistry is straightforward and can be scaled easily. The indole N-substituent can be introduced later to make the synthesis more modular, and the resolution is straightforward late in the synthesis. Additionally, the presence of the nitro group in CPA **2** was chosen for two reasons: 1) the possibility to further functionalize at this position through reduction, diazotization, and metal-catalyzed cross-coupling, and 2) immobilization on a solid support via reduction and reaction of the aniline with an electrophile such as polystyrene isocyanate.

#### DOPO scaffold

We previously reported the syntheses of both enantiomers of 8-phenyl DOPO **3** [38]. The syntheses proceed in only three steps (including the separation of the (*S*)-1-phenylethylamine-derived phosphonamide diastereoisomers) with *S*_P_-**3** and *R*_P_-**3** obtained in 13% and 9% respectively starting from 2,6-diphenylphenol.

**Scheme 4:**
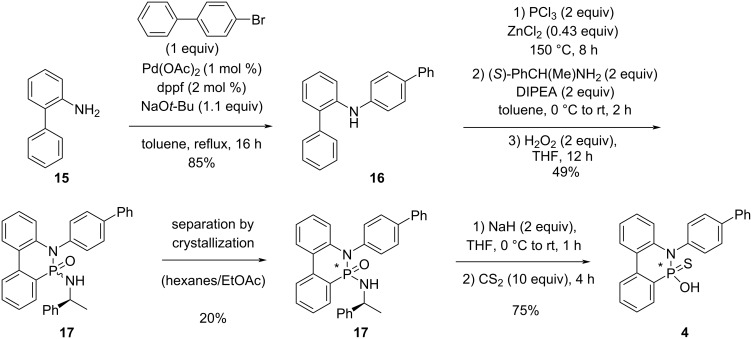
Synthesis of *N*-biphenyl-DOPO CPA **4**.

Finally, *N*-biphenyl-DOPO CPA **4** was synthesized in four steps as shown in Scheme 4. Although compound **16** is commercially available, it was synthesized from 2-aminobiphenyl according to the literature [47]. Subsequent reaction with phosphorus trichloride and electrophilic aromatic substitution gave a chlorophosphine intermediate, which was directly reacted with (*S*)-1-phenylethylamine, then hydrogen peroxide. Phosphonamide diastereoisomers **17** were obtained in moderate yield. Crystallization gave a single diastereoisomer in 20% yield. Stec reaction [38–40] finally gave the desired CPA **4**. Although the entire sequence proceeded in only 6% overall yield, it was conducted on a multigram-scale so that more than 0.4 g of **4** was obtained.

#### Evaluation of the catalysts

**Scheme 5:**
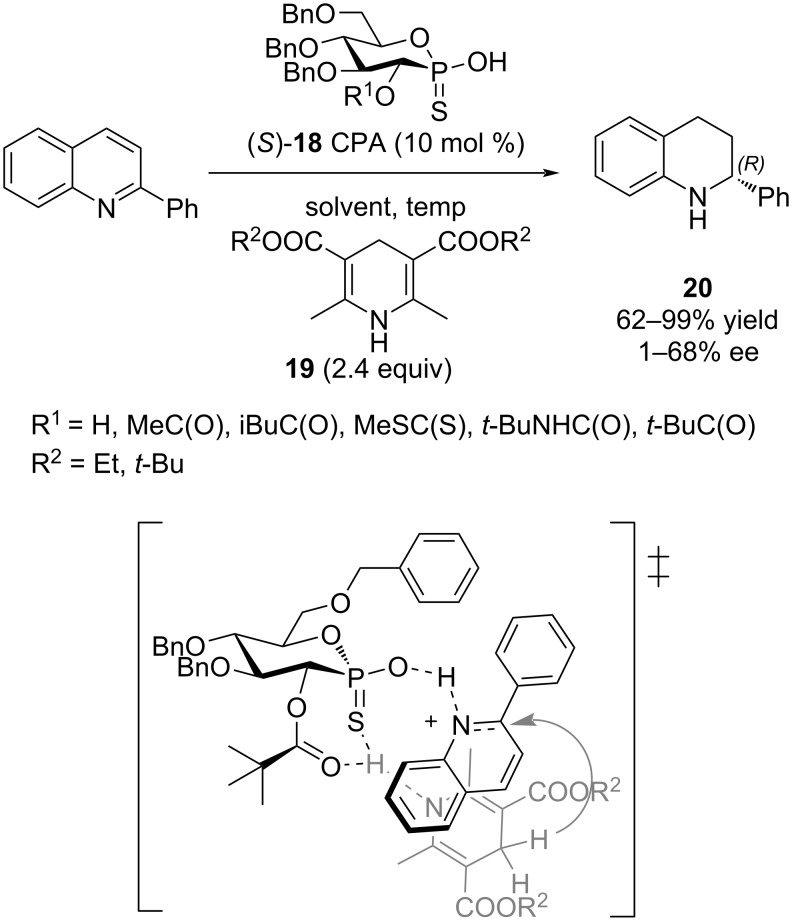
Transfer hydrogenation of 2-phenylquinoline and transition-state proposed by Guinchard and coworkers [28].

With our various CPAs **2**–**4** in hand, their evaluation in asymmetric organocatalysis was conducted. The reaction could have been chosen from a tremendous number of possibilities [1–17]. We selected the one Guinchard used to evaluate his thioacid hybrid-CPAs (Scheme 5) [31]. The transfer hydrogenation of 2-phenylquinoline with a Hantzsch ester **19** is a test reaction commonly used in asymmetric synthesis. The best performing of Guinchard's thiophostones **18** was the pivalate ester (R^1^ = *t*-BuC(O)) with an 86% yield of **20** and a 52% ee (**19** R^2^ = Et (2.4 equiv), toluene, 60 °C). Further optimization with the pivalate led to **20** in 82% yield and 68% ee (**19** R^2^ = *t*-Bu (2 equiv), cyclopentyl methyl ether, 22 °C).

To account for the best results observed with pivalate **18**, Guinchard and coworkers proposed the transition-state shown in Scheme 5 [31]. Based on the fact that the cis-configuration between the sulfur and the pivalate was absolutely required for enantioselectivity, an interaction between both the sulfur and pivalate carbonyl oxygen with the hydrogen of Hantzsch ester's NH was proposed (Scheme 5). Thus, rather weak interactions might still be important in the assembly of a ternary complex and the enantioselectivity of the reaction.

The evaluation of the catalysts is shown in Table 1. CPA **4** was completely ineffective at inducing chirality (Table 1, entry 1) and catalyst **2** was not much better (entry 2). Catalyst **3** on the other hand showed a modest induction (entry 3).

**Table 1:** *P*-stereogenic CPAs in the transfer hydrogenation of quinolines.

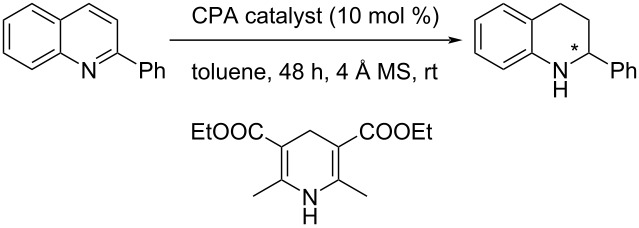			

Entry	CPA catalyst	Yield (%)	ee (%)^a^
1	**4**	95	2
2	**2**	90	10
3	**3**	95	30

^a^Enantiomeric excess was determined by HPLC with a Chiracel OD-H column (hexane/iPrOH 95:5, 1 min/mL).

## Conclusion

Exploratory efforts toward new C1-symmetrical CPAs were described. Four CPAs were synthesized and three evaluated. The syntheses are straightforward, inexpensive, and scalable. Resolution via the separation of diastereoisomeric phosphorus amides could be accomplished easily, either by chromatography over silica gel or crystallization. Subsequent Stec reaction proved to be a reliable method to convert the resolved amide into the chiral thiophosphorus acids.

The CPAs synthesized clearly failed to induce any significant asymmetry. It is interesting to note, however, that the enantiomeric excess increases with an increase in bond length separation between the phosphorus and the R group. From the reaction evaluation we found that dual activation might be required from the catalyst in certain enantioselective reactions. Thus, CPA platforms that reintroduce a dual donor–acceptor role, such as *P*-stereogenic triflamide CPAs P(O)NHSO_2_CF_3_, is currently under investigation since BINOL-derived triflamides have been successful [48–49]. Another possibility would be to look at reactions in which the catalyst would not require a Brønsted basic site. Both directions are currently under investigation and results will be shared in due course.

## Supporting Information

File 1 Experimental procedures and copies of spectra.
