# Supplementary material for: Design, synthesis, and evaluation of chiral thiophosphorus acids as organocatalysts
Source: Beilstein J Org Chem. 2022 Oct 17;18:1471–8. doi: 10.3762/bjoc.18.154 (PMC9592963; doi:10.3762/bjoc.18.154)
Supplement: File 1 — Experimental procedures and copies of spectra. [file Beilstein_J_Org_Chem-18-1471-s001.pdf]

BEILSTEIN JOURNAL OF ORGANIC CHEMISTRY

Supporting Information

for

Design, synthesis, and evaluation of chiral thiophosphorus acids as organocatalysts

Karen R. Winters and Jean-Luc Montchamp

Beilstein J. Org. Chem. **2022,** *18,* 1471–1478. doi:10.3762/bjoc.18.154

Experimental procedures and copies of spectra

License and Terms: This is a supporting information file under the terms of the Creative Commons Attribution License (https://creativecommons.org/licenses/by/4.0). Please note that the reuse, redistribution and reproduction in particular requires that the author(s) and source are credited and that individual graphics may be subject to special legal provisions.
The license is subject to the *Beilstein Journal of Organic Chemistry* terms and conditions: (https://www.beilstein-journals.org/bjoc/terms)

Table of contents **Page**

General chemistry S2

Reagents and solvents S2

Experimental procedures S3

HPLC chromatograms S17

NMR spectra S22

General chemistry.

^{1}H NMR spectra were recorded on a 400 MHz Bruker Avance spectrometer. Chemical shifts for ^{1}H NMR spectra (in parts per million) are relative to internal tetramethylsilane (Me_4Si, δ = 0.00 ppm) with deuterated chloroform. $^{13}C\{^1H\}$NMR spectra were recorded at 101 MHz. Chemical shifts for $^{13}C\{^1H\}$ NMR spectra are reported (in parts per million) relative to $CDCl_3$ (δ = 77.0 ppm). ^{31}P NMR spectra were recorded at 162 MHz, and chemical shifts reported (in parts per million) are relative to external 85% phosphoric acid (δ = 0.0 ppm). Flash chromatography experiments were carried out on silica gel premium Rf grade (40–75 µm). Ethyl acetate/hexane or ethyl acetate/methanol mixtures were used as the eluent for chromatographic purifications. TLC plates were visualized by UV or immersion in permanganate potassium (3 g of $KMnO_4$, 20 g of K_2CO_3, 5 mL of 5% aq NaOH, 300 mL of water) followed by heating. Chiral HPLC analyses were recorded on an Agilent 1200 Series HPLC system. Chiral HPLC resolutions were done with an *(S,S)*-Whelk-01 Column (250 × 4.6 mm, 5 µm) from Regis Technologies or CHIRALCEL OD-H from Daicel, using hexanes/isopropanol mixtures as the mobile phase. Mass spectrometry was provided by Louisiana State University Mass Spectrometry Resource. High-resolution mass spectra (HRMS) were obtained by electrospray ionization using a TOF analyzer.

Reagent and solvents.

All starting materials were purchased from commercial sources and used as received, unless otherwise noted. Anhydrous THF and DMF were purchased and used as received. The solvents were distilled under N_2 and dried according to standard procedures (CH_3CN, toluene, and dichloromethane from CaH_2).

Experimental procedures.

N-Phenyl tryptophol (**6**)

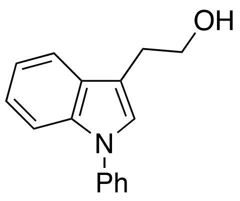

To a screw-cap test tube was added CuI (0.05 equiv, 1.5 mmol), tryptophol (**5**, 1.0 equiv, 31.0 mmol), K_3PO_4 (2.1 equiv, 65.1 mmol) and the vessel was evacuated and back-filled with nitrogen. Iodobenzene (1.2 equiv, 37.2 mmol), *trans*-N^1,N^2-dimethylcyclohexane-1,2-diamine (10 mol %, 3.1 mmol) and toluene (32 mL) were added under nitrogen. The reaction tube was sealed, and the contents were stirred, with heating from an oil bath at 110 °C overnight. The reaction mixture was cooled to ambient temperature, diluted with ethyl acetate (20 mL), and filtered through a plug of Celite, eluted with additional ethyl acetate (20 mL). The filtrate was concentrated under vacuum and the resulting residue was purified by column chromatography on silica gel (hexanes/ethyl acetate 95:05 to 50:50) to provide **6** as a colorless oil (7.3 g, 98%). ^{1}H NMR (400 MHz, $CDCl_3$) δ 7.72 (ddd, *J* = 7.8, 1.4, 0.8 Hz, 1H), 7.62 (dt, *J* = 8.2, 0.9 Hz, 1H), 7.54 (d, *J* = 5.7 Hz, 4H), 7.44 – 7.33 (m, 1H), 7.31 – 7.28 (m, 2H), 7.27 – 7.19 (m, 1H), 4.00 (t, *J* = 6.4 Hz, 2H), 3.13 (td, *J* = 6.4, 0.9 Hz, 2H), 1.72 (s, 1H); ^{13}C NMR (101 MHz, $CDCl_3$) δ 139.7, 136.3, 129.7, 128.9, 126.30, 126.28, 124.2, 122.7, 120.1, 119.2, 113.5, 110.7, 62.6, 28.7.

Methyl (2-(1-phenyl-1H-indol-3-yl)ethyl)-H-phosphonate (**7**)

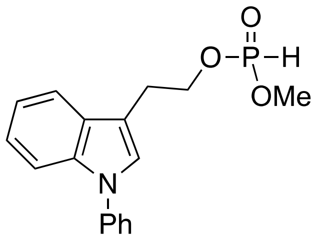

Synthesis of the *tert*-butylamine methyl phosphonate salt was made according to a known procedure.[1] To a solution of *tert*-butylamine methyl phosphonate salt (1.0 equiv, 1.7 mmol) in anhydrous CH_2Cl_2 (22 mL) was added pivaloyl chloride (1.0 equiv, 1.7 mmol) at room temperature under nitrogen. After stirring for 1 h, **6** (1.0 equiv, 1.7 mmol) was added at room temperature and left to stir overnight. The mixture was washed with saturated aqueous $NaHCO_3$ (10 mL) and extracted with CH_2Cl_2 (10 mL × 2). The combined extracts were washed with brine, dried over anhydrous $MgSO_4$, filtered, and concentrated under vacuum. The residue was purified by column chromatography on silica gel (hexanes/ethyl acetate 8:2 to 6:4 to 2:8) to give **7** colorless oil (0.5 g, 89%). ^{31}P NMR (162 MHz, $CDCl_3$) δ 9.25 (d); ^{1}H NMR (400 MHz, $CDCl_3$) δ 7.73 – 7.69 (m, 1H), 7.68 – 5.93 (d, *J* = 698.2 Hz, 1H), 7.63 – 7.57 (m, 1H), 7.40 – 7.34 (m, 1H), 7.32 – 7.20 (m, 3H), 4.50 – 4.37 (m, 2H), 3.74 (d, *J* = 12.0 Hz, 3H), 3.28 (d, *J* = 0.9 Hz, 2H); ^{13}C NMR (101 MHz, $CDCl_3$) δ 139.6, 136.0, 129.7, 128.7, 126.4 (d, *J* = 12.8 Hz), 124.2, 122.7, 120.3, 119.0, 112.2, 110.7, 65.6 (d, *J* = 6.2 Hz), 52.0 (d, *J* = 5.8 Hz), 26.6 (d, *J* = 6.0 Hz). HRMS (EI+) *m*/*z* $[M + H]^+$ calcd for $C_{17}H_{18}NO_3P$ 315.1019, found 316.1093.

1-Methoxy-9-phenyl-3,4,9-trihydro[1,2]oxaphosphinino[3,4-b]indole 1-oxide (**8**)

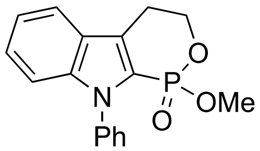

To a solution of **7** (1.0 equiv, 2.2 mmol) in acetic acid (15 mL) was added $Mn(OAc)_2$ (5 mol %, 0.111 mmol), MnO_2 (85% activated, 3.0 equiv, 6.6 mmol), and sodium acetate (3.0 equiv, 6.6 mmol). The suspension was stirred overnight at 70 °C under nitrogen. The reaction mixture was cooled to room temperature, diluted with ethyl acetate (20 mL) and a 0.1 M aqueous solution of $Na_2S_2O_4$ saturated with NaCl (20 mL) was added. The mixture was stirred for 5 min and the suspension was filtered

over Celite. The organic layer was washed with aqueous solutions of $Na_2S_2O_4$ saturated with NaCl (20 mL × 2) and saturated $NaHCO_3$ (20 mL × 5). The combined extracts were washed with brine, dried with $MgSO_4$, filtered, and concentrated under vacuum. The residue was purified by column chromatography on silica gel (hexanes/ethyl acetate 90:10 to 0:100) to give **8** as a colorless oil (0.3 g, 43%). ^{31}P NMR (162 MHz, $CDCl_3$) δ 7.1 ppm (s); ^{1}H NMR (400 MHz, $CDCl_3$) δ 7.66 – 7.61 (m, 3H), 7.57 (m, 2H), 7.51 – 7.43 (m, 1H), 7.42 – 7.29 (m, 2H), 7.24 (m, 1H), 4.82 – 4.57 (m, 2H), 3.48 (d, *J* = 11.6 Hz, 3H), 3.24 (dddd, *J* = 16.7, 6.7, 4.4, 2.7 Hz, 2H); HRMS (EI+) *m/z* $[M + H]^+$ calcd for $C_{17}H_{16}NO_3P$ 313.0862, found 314.0951.

1-Methoxy-9-phenyl-3,4,9-trihydro[1,2]oxaphosphinino[3,4-b]indole 1-sulfide (**9**)

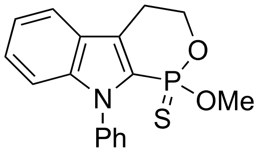

To a solution of **8** (1.0 equiv, 6.7 mmol) in anhydrous toluene (40 mL) was added Lawesson's Reagent (0.6 equiv, 4.0 mmol) under nitrogen. The solution was refluxed for 16 h, cooled to room temperature, and concentrated under vacuum. The residue was purified by column chromatography on silica gel (hexanes/ethyl acetate 98:02 to 80:20) to give **9** as a colorless oil (1.2 g, 54%). ^{31}P NMR (162 MHz, $CDCl_3$) δ 67.8 ppm (s); ^{1}H NMR (400 MHz, $CDCl_3$) δ 7.69 – 7.63 (m, 1H), 7.53 (m, 5H), 7.33 (dd, *J* = 8.6, 6.7 Hz, 1H), 7.23 (dd, *J* = 8.0, 6.3 Hz, 2H), 4.68 (dq, *J* = 17.6, 4.3 Hz, 2H), 3.66 (d, *J* = 14.5 Hz, 3H), 3.42 – 3.10 (m, 2H).

1-Hydroxy-3,4,9-trihydro-[1,2]oxaphosphinino[3,4-b]indole 1-sulfide (**1**)

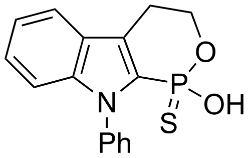

To a reaction tube was added **9** (1.0 equiv, 0.16 mmol) in anhydrous CH_3CN (6 mL) and 1,4-diazabicyclo[2.2.2]octane (1.0 equiv, 0.16 mmol). The tube was placed in a synthesizer and stirred for 12 h at 85 °C under nitrogen. The solution was cooled to room temperature, acidified with 1 M HCl (10 mL) and extracted with CH_2Cl_2 (10 mL × 2). The combined extracts were dried with $MgSO_4$, filtered and condensed under vacuum to yield **1** (NMR yield: 100%). ^{31}P NMR (162 MHz, $CDCl_3$) δ 49.0 ppm (s).

3-Allylindole (**10**)

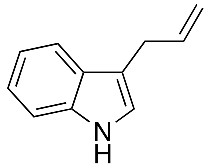

To a reaction tube was added indole (1 equiv, 20 mmol) and allyl alcohol (1.5 equiv, 30 mmol) in THF (90 mL). The tube was flushed with argon for 10 minutes. Next, $Pd(PPh_3)_4$ (5 mol %, 1 mmol) and Et_3B (30 mol %, 6 mmol, 1 M in THF) were added and flushed with argon for 10 minutes. The tube was sealed and brought to 50 °C in an oil bath for 16 h. The reaction mixture was then cooled to rt and diluted with ethyl acetate (40 mL). The solution was transferred to a separatory funnel and washed with a saturated aqueous solution of $NaHCO_3$ and brine. The organic layer was dried over $MgSO_4$, filtered and concentrated under vacuum. The crude product was purified by column chromatography on silica gel (hexanes/ethyl acetate 95:5) to give **10** as a yellow oil (2.5 g, 80%). ^1H NMR (400 MHz, $CDCl_3$) δ 7.83 – 7.76 (m, 2H), 7.45 – 7.27 (m, 3H), 7.01 (dt, J = 2.1, 1.0 Hz, 1H), 6.25 (ddt, J = 17.1, 10.0, 6.5 Hz, 1H), 5.44 – 5.12 (m, 2H), 3.74 – 3.65 (m, 2H); ^{13}C NMR (101 MHz, $CDCl_3$) δ 137.5, 136.5, 127.5, 122.1, 121.9, 119.4, 119.3, 115.4, 114.5, 111.3, 30.0.

Ethyl (3-(1H-indol-3-yl)propyl)phosphinate (**11**)

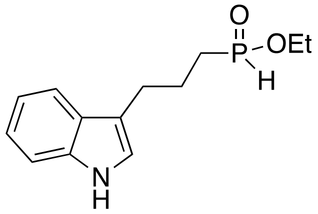

In a round-bottomed flask was added $EtOP(O)H_2$ (1.5 equiv, 47.7 mmol, 0.5 M in CH_3CN), **10** (1 equiv, 31.8 mmol), $Pd_2dba_3{\cdot}CHCl_3$ (0.5 mol %, 0.3 mmol), and xantphos (1.2 mol %, 0.38 mmol). The flask was flushed with argon for 10 min then brought to reflux for 18 h. After cooling to rt, the mixture was diluted with ethyl acetate (60 mL). The solution was transferred to a separatory funnel and washed with a saturated aqueous solution of $NaHCO_3$ and brine. The organic layer was dried over $MgSO_4$, filtered, and concentrated under vacuum. The crude product was purified by column chromatography on silica gel (ethyl acetate/methanol 100:0 to 90:10) to give **11** as an oil (4.88 g, 63%). ^{31}P NMR (162 MHz, $CDCl_3$) δ 39.2 (d, *J* = 528.3 Hz); ^{1}H NMR (400 MHz, $CDCl_3$) δ 7.75 (dt, *J* = 526.7, 1.9 Hz, 1H), 7.61 (dt, *J* = 8.0, 1.0 Hz, 1H), 7.39 (dt, *J* = 8.1, 1.0 Hz, 1H), 7.21 (ddd, *J* = 8.2, 7.0, 1.3 Hz, 1H), 7.14 (ddd, *J* = 8.0, 7.0, 1.1 Hz, 1H), 6.99 (d, *J* = 2.4 Hz, 1H), 4.36 – 3.94 (m, 3H), 2.95 – 2.86 (m, 2H), 2.06 – 1.97 (m, 2H), 1.87 (dddd, *J* = 15.2, 9.3, 6.7, 3.9 Hz, 2H), 1.37 (d, *J* = 7.1 Hz, 3H); ^{13}C NMR (101 MHz, $CDCl_3$) δ 136.4, 127.3, 122.1, 121.7, 119.3 (d, *J* = 54.9 Hz), 114.9, 111.2, 62.4 (d, *J* = 7.0 Hz), 28.9, 27.9, 25.8 (d, *J* = 16.5 Hz), 21.4 (d, *J* = 2.8 Hz), 16.3 (d, *J* = 6.2 Hz); HRMS (EI+) *m*/*z* $[M + H]^+$ calcd for $C_{13}H_{18}NO_2P$ 251.107, found 252.1148.

1-Ethoxy-2,3,4,9-tetrahydrophosphinino[2,3-b]indole 1-oxide (**12**)

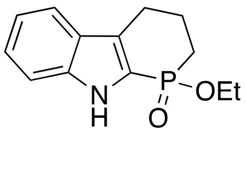

To a reaction tube was added **11** (1 equiv, 1.2 mmol), silver(I) acetate (3 equiv, 3.5 mmol) in DCE (8 mL) and flushed with argon for 10 min. The tube was brought to 90 °C in an oil bath for 18 h. The reaction mixture was then cooled to rt, diluted with DCM (15 mL) and filtered over Celite. The filtrate was concentrated under vacuum and the crude product purified by column chromatography on silica gel (hexanes/ethyl acetate 20:80 to 10:90) to afford **12** as a tan solid (0.2 g, 73%). ^{31}P NMR (162 MHz, $CDCl_3$) δ 34.1 (s); ^{1}H NMR (400 MHz, $CDCl_3$) δ 11.74 (d, *J* = 6.6 Hz, 1H), 7.67 – 7.53 (m, 2H), 7.36 – 7.25 (m, 1H), 7.14 (ddd, *J* = 8.1, 7.1, 1.0 Hz, 1H), 4.36 – 4.10 (m, 2H), 3.19 – 2.82 (m, 2H), 2.68 – 2.25 (m, 3H), 2.23 – 2.00 (m, 1H), 1.24 (t, *J* = 7.1 Hz, 3H); ^{13}C NMR (101 MHz, $CDCl_3$) δ 138.8 (d, *J* = 11.6 Hz), 126.1 (d, *J* = 12.0 Hz), 125.7 (d, *J* = 15.0 Hz), 124.6, 123.5, 122.2, 120.1 – 119.3 (m), 112.7 (d, *J* = 1.4 Hz), 61.9 (d, *J* = 6.8 Hz), 27.5, 26.5, 22.6 (dd, *J* = 10.0, 5.0 Hz), 16.5 (d, *J* = 6.7 Hz); HRMS (EI+) *m/z* $[M + H]^+$ calcd for $C_{13}H_{16}NO_2P$ 249.0913, found 250.0990.

1-Ethoxy-9-phenyl-2,3,4,9-tetrahydrophosphinino[2,3-b]indole 1-oxide (**13a**)

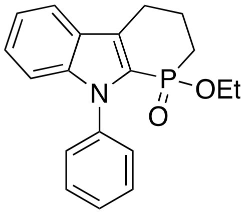

To a round-bottomed flask was added **12** (1 equiv, 4 mmol), iodobenzene (1.2 equiv, 4.8 mmol), K_3PO_4 (2.1 equiv, 8.4 mmol), CuI (5 mol %, 0.2 mmol), and DMEDA (10 mol %, 0.4 mmol) in toluene (20 mL). The flask was flushed with argon for 10 min then the mixture was brought to reflux for 18 h. After cooling to rt, the solvent was evaporated under vacuum. The crude was dissolved in DCM and transferred to a separatory funnel. The organic layer was washed with NH_4Cl (aq), then washed with brine. The organic layer was separated and dried with $MgSO_4$, filtered, and concentrated under vacuum. The crude product was purified by column

chromatography on silica gel (hexanes/ethyl acetate 25:75) to afford the pure product **13a** as a colorless oil (1 g, 77%). ^{31}P NMR (162 MHz, $CDCl_3$) δ 32.2 (s); ^{1}H NMR (400 MHz, $CDCl_3$) δ 7.66 (t, *J* = 8.7 Hz, 2H), 7.55 (t, *J* = 7.7 Hz, 2H), 7.49 – 7.41 (m, 1H), 7.35 – 7.25 (m, 3H), 7.20 (ddd, *J* = 7.8, 6.3, 1.6 Hz, 1H), 3.89 – 3.73 (m, 1H), 3.67 – 3.50 (m, 1H), 3.12 – 2.98 (m, 2H), 2.50 – 2.28 (m, 2H), 2.26 – 2.01 (m, 2H), 1.03 (d, *J* = 7.0 Hz, 3H); ^{13}C NMR (101 MHz, $CDCl_3$) δ 139.4 (d, *J* = 9.2 Hz), 138.5, 129.2, 128.0, 127.8 (d, *J* = 8.1 Hz), 127.6 (d, *J* = 3.8 Hz), 126.5 (d, *J* = 12.5 Hz), 126.2, 125.3, 120.4, 120.0 (d, *J* = 1.7 Hz), 111.1, 60.9 (d, *J* = 6.2 Hz), 28.4 (d, *J* = 99.1 Hz), 23.1 (d, *J* = 4.0 Hz), 21.8 (d, *J* = 5.8 Hz), 16.4 (d, *J* = 6.2 Hz); HRMS (EI+) *m/z* $[M + H]^+$ calcd for $C_{19}H_{20}NO_2P$ 325.1226, found 326.1303.

1-Ethoxy-9-(p-nitrophenyl)-2,3,4,9-tetrahydrophosphinino[2,3-b]indole 1-oxide (**13b**)

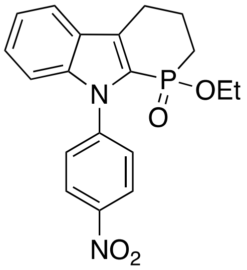

To a round-bottomed flask was added **12** (1 equiv, 1.5 mmol), iodo-nitrobenzene (1.2 equiv, 1.8 mmol), K_3PO_4 (2.1 equiv, 3.1 mmol), CuI (5 mol %, 0.075 mmol), and DMEDA (10 mol %, 0.15 mmol) in toluene (10 mL). The flask was flushed with argon for 10 min then the mixture was brought to reflux for 18 h. After cooling to rt, the solvent was evaporated under vacuum. The crude was dissolved in DCM and transferred to a separatory funnel. The organic layer was washed with NH_4Cl (sat. aq), then washed with brine. The organic layer was separated and dried with $MgSO_4$, filtered, and concentrated under vacuum. The crude product was purified by column chromatography on silica gel (hexanes/ethyl acetate 25:75) to afford **13b** as a yellow solid (0.4 g, 73%). ^{31}P NMR (162 MHz, $CDCl_3$) δ 32.2 (s); ^{1}H NMR (400 MHz, $CDCl_3$)

δ 8.47 – 8.38 (m, 2H), 7.97 – 7.89 (m, 2H), 7.67 (dt, J = 8.0, 1.1 Hz, 1H), 7.42 – 7.32 (m, 2H), 7.30 – 7.22 (m, 1H), 3.96 – 3.73 (m, 2H), 3.05 (td, J = 6.0, 3.1 Hz, 2H), 2.38 (s, 2H), 2.29 – 2.04 (m, 2H), 1.11 (t, J = 7.0 Hz, 3H); ^{13}C NMR (101 MHz, $CDCl_3$) δ 146.4, 144.4, 138.8 (d, J = 8.8 Hz), 129.8 (d, J = 14.0 Hz), 127.8, 127.2 (d, J = 12.3 Hz), 126.9 (d, J = 137.5 Hz), 126.3, 124.8, 121.5, 120.4 (d, J = 1.5 Hz), 110.7 (d, J = 1.6 Hz), 61.1 (d, J = 6.0 Hz), 27.9 (d, J = 98.2 Hz), 23.1 (d, J = 4.1 Hz), 21.5 (d, J = 6.1 Hz), 16.5 (d, J = 5.9 Hz); HRMS (EI+) m/z $[M + H]^+$ calcd for $C_{19}H_{19}N_2O_4P$ 370.1077, found 371.1151.

9-Phenyl-1-(((S)-1-phenylethyl)amino)-2,3,4,9-tetrahydrophosphinino[2,3-b]indole 1-oxide (**14b**)

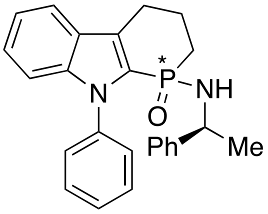

To a round-bottomed flask was added **13a** (1 equiv, 2 mmol) in DCM (5 mL). Oxalyl chloride (2 equiv, 4 mmol) was added dropwise followed by DMF (10 mol %, 0.2 mmol). The reaction mixture was brought to reflux and stirred for 24 h under argon. To a separate flask was added (S)-1-phenylethylamine (2 equiv, 4 mmol), Et_3N (2 equiv, 4 mmol), and DMAP (0.1 equiv, 0.2 mmol) in DCM (5 mL). To this the P(O)Cl mixture was added via cannula at rt and stirred for 24 h. The reaction mixture was transferred to a separatory funnel and washed with $NaHCO_3$ (sat. aq), NH_4Cl (sat. aq), and then brine. The organic layer was separated, dried with $MgSO_4$, filtered, and concentrated under vacuum. The mixture was concentrated under vacuum and directly purified and resolved by column chromatography on silica gel (hexanes/ethyl acetate 30:70) to afford the phosphoramide **14a** as a beige solid (0.7 g, 85%, resolved yield 47%). *Mixture:* ^{31}P NMR (162 MHz, $CDCl_3$) δ 23.5 (s), 20.4 (s); *Resolved:* ^{31}P NMR

(162 MHz, $CDCl_3$) δ 23.6 (s); ^{1}H NMR (400 MHz, $CDCl_3$) δ 7.70 – 7.56 (m, 2H), 7.55 – 7.39 (m, 3H), 7.36 – 7.28 (m, 3H), 7.26 – 7.18 (m, 4H), 7.16 (d, *J* = 1.9 Hz, 2H), 4.28 (dtt, *J* = 13.6, 9.1, 4.6 Hz, 1H), 3.02 – 2.80 (m, 2H), 2.65 (dd, *J* = 10.6, 8.9 Hz, 1H), 2.26 (ddtdd, *J* = 17.0, 8.0, 6.0, 4.1, 2.1 Hz, 1H), 2.11 (dddd, *J* = 20.6, 9.4, 4.4, 2.0 Hz, 1H), 2.04 – 1.83 (m, 1H), 1.82 – 1.66 (m, 1H), 1.02 (d, *J* = 6.8 Hz, 3H); ^{13}C NMR (101 MHz, $CDCl_3$) δ 145.9 (d, *J* = 2.9 Hz), 139.3 (d, *J* = 8.2 Hz), 138.5, 129.2, 128.8, 128.5, 127.9, 127.7 (d, *J* = 3.8 Hz), 126.9, 126.7 (d, *J* = 11.4 Hz), 126.2, 125.8, 125.3, 120.5, 120.0 (d, *J* = 1.5 Hz), 111.1 (d, *J* = 1.5 Hz), 49.9, 30.6 (d, *J* = 93.6 Hz), 25.5 (d, *J* = 6.0 Hz), 23.3 (d, *J* = 3.4 Hz), 21.5 (d, *J* = 5.8 Hz).

9-(4-Nitrophenyl)-1-(((S)-1-phenylethyl)amino)-2,3,4,9-tetrahydrophosphinino[2,3-b]indole 1-oxide (**14b**)

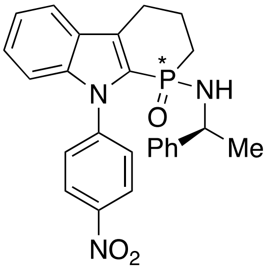

To a round-bottomed flask was added **13b** (1 equiv, 0.4 mmol) in DCM (2 mL). Oxalyl chloride (2 equiv, 0.81 mmol) was added dropwise followed by DMF (10 mol %, 0.04 mmol). The reaction mixture was brought to reflux and stirred for 24 h under argon. To a separate flask was added (*S*)-1-phenylethylamine (2 equiv, 0.81 mmol), Et_3N (2 equiv, 0.81 mmol), and DMAP (0.1 equiv, 0.04 mmol) in DCM (1 mL). To this the P(O)Cl mixture was added via cannula at rt, and stirred for 24 h. The reaction mixture was transferred to a separatory funnel and washed with $NaHCO_3$ (sat. aq), NH_4Cl (sat. aq), and then brine. The organic layer was separated, dried with $MgSO_4$, filtered, and concentrated under vacuum. The mixture was concentrated under vacuum and directly purified and resolved by column chromatography on silica gel

(hexanes/ethyl acetate 30:70) to afford the product **14b** (0.12 g, 67%, resolved 20%). *Mixture:* ^{31}P NMR (162 MHz, $CDCl_3$) δ 22.3 (s), 21.3 (s); *Resolved:* ^{31}P NMR (162 MHz, $CDCl_3$) δ 20.4 (s); ^{1}H NMR (400 MHz, $CDCl_3$) δ 8.34 (d, *J* = 8.6 Hz, 2H), 7.68 (d, *J* = 8.4 Hz, 1H), 7.42 – 7.38 (m, 2H), 7.24 (td, *J* = 13.6, 8.1 Hz, 5H), 7.06 – 7.01 (m, 2H), 6.96 (d, *J* = 7.1 Hz, 1H), 3.88 (q, *J* = 8.0 Hz, 1H), 3.08 (dd, *J* = 16.9, 4.4 Hz, 2H), 2.40 (s, 3H), 2.22 – 2.17 (m, 1H), 2.07 – 1.92 (m, 1H), 1.03 (d, *J* = 6.7 Hz, 3H); ^{13}C NMR (101 MHz, $CDCl_3$) δ 146.0, 144.8, 144.1, 138.6 (d, *J* = 7.5 Hz), 131.0, 130.5, 128.6 (d, *J* = 4.0 Hz), 127.2 (d, *J* = 8.3 Hz), 126.3, 125.5, 124.8, 121.6, 120.5, 110.8 (d, *J* = 8.8 Hz), 50.5 (d, *J* = 15.4 Hz), 30.6 (d, *J* = 9.1 Hz), 29.7 (d, *J* = 8.9 Hz), 26.1 (dd, *J* = 13.0, 5.9 Hz), 23.3 (dd, *J* = 7.3, 3.7 Hz), 21.2 (d, *J* = 5.8 Hz), 20.8 (d, *J* = 5.8 Hz); HRMS (EI+) *m/z* $[M + H]^+$ calcd for $C_{25}H_{24}N_3O_3P$ 445.1550, found 446.1633.

*1-Hydroxy-9-(4-nitrophenyl)-2,3,4,9-tetrahydrophosphinino[2,3-b]indole 1-sulfide (**2**)*

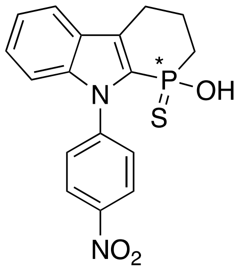

To a round-bottomed flask was added the (R_p) or (S_p)-**14b** (1 equiv, 0.9 mmol) in THF (3 mL) under argon. The reaction mixture was cooled to 0 °C in an ice bath. NaH (3 equiv, 2.7 mmol, 60% in mineral oil) was added in one portion and the mixture brought to rt and stirred for 1 h, then CS_2 (10 equiv, 9 mmol) was added dropwise and the reaction stirred at rt overnight. The reaction mixture was cooled to rt and diluted with ethyl acetate (30 mL) and transferred to a separatory funnel. The organic layer was extracted with $NaHCO_3$ (sat. aq, 3 ×), and the layers separated. The basic layer was acidified with 3 M HCl (pH 1) and extracted with ethyl acetate. The organic layer

was separated and dried with $MgSO_4$, filtered and concentrated under vacuum to afford the product **2** as a yellow solid (0.3 g, 80%). ^{31}P NMR (162 MHz, DMSO-d_6) δ 58.1 (s); ^{1}H NMR (400 MHz, DMSO-d_6) δ 11.94 (s, 1H), 8.45 – 8.36 (m, 2H), 7.91 – 7.83 (m, 2H), 7.71 (dt, J = 7.9, 1.0 Hz, 1H), 7.42 – 7.27 (m, 2H), 7.24 (ddd, J = 7.9, 6.6, 1.3 Hz, 1H), 3.11 – 2.99 (m, 1H), 2.99 – 2.86 (m, 1H), 2.39 (ddt, J = 17.3, 9.8, 3.4 Hz, 1H), 2.33 – 2.08 (m, 3H); ^{13}C NMR (101 MHz, DMSO-d_6) δ 146.4, 144.6, 138.6 (d, J = 7.9 Hz), 130.4 (d, J = 109.2 Hz), 128.9, 126.9 (d, J = 11.0 Hz), 126.2, 125.1, 124.9 (d, J = 11.8 Hz), 121.7, 121.0, 110.8, 37.3 (d, J = 78.5 Hz), 23.1 (d, J = 3.3 Hz), 20.9 (d, J = 7.0 Hz); HRMS (EI+) m/z $[M + H]^+$ calcd for $C_{17}H_{15}N_2O_3P$ 358.0536, found 359.0618.

N-(1,1'-Biphenyl-4-yl)-1,1'-biphenyl-2-amine (**16**)

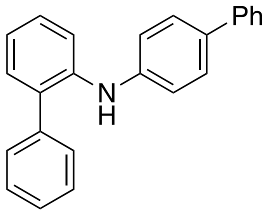

To a round-bottomed flask was added 1-aminobiphenyl (1 equiv, 36 mmol) and 4-bromobiphenyl (1 equiv, 36 mmol) in toluene (67 mL). The reaction mixture was flushed with argon for 10 min, then $Pd(OAc)_2$ (1 mol %, 0.37 mmol), dppf (2 mol %, 0.66 mmol) and NaO*t*-Bu (1.1 equiv, 47 mmol) were added and the reaction mixture brought to reflux for 16 h. The mixture was then cooled to rt, and H_2O (30 mL) was added then the mixture was transferred to a separatory funnel. The organic layer was washed with H_2O, extracted with toluene, and the layers were separated. The organic layer was dried with $MgSO_4$, filtered, and concentrated in vacuum. The crude product was purified by column chromatography to yield the product **16** (2.5 g, 85%).[2] ^{1}H NMR (400 MHz, $CDCl_3$) δ 7.65 – 7.58 (m, 2H), 7.56 – 7.53 (m, 2H), 7.53 – 7.48 (m, 5H),

7.47 – 7.39 (m, 3H), 7.34 (td, J = 6.8, 1.6 Hz, 3H), 7.19 – 7.13 (m, 2H), 7.09 (dd, J = 7.4, 1.2 Hz, 1H), 5.72 (s, 1H).

5-([1,1'-Biphenyl]-4-yl)-6-((1-phenylethyl)amino)-5H-dibenzo[c,e][1,2]azaphosphinine 6-oxide (**17**)

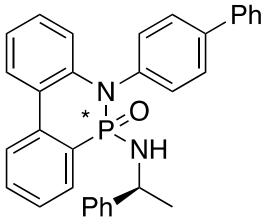

In a manner similar to [38] under neat conditions to a round-bottomed flask was added **16** (1 equiv, 7.8 mmol) and phosphorus trichloride (2.3 equiv, 18.13 mmol), and the mixture brought to 50 °C in an oil bath and stirred for 3 h under argon. The reaction mixture was cooled to rt, and zinc chloride (0.43 equiv, 0.36 mmol) was added. The reaction mixture was brought to 150 °C and stirred for 8 h under argon. After cooling to 0 °C, the crude was solubilized in toluene (30 mL), and DIPEA (2.0 equiv, 15.6 mmol) and (*S*)-1-phenylethylamine (2.0 equiv, 15.6 mmol) were added and stirred at rt for 2 h under argon. To the reaction mixture H_2O_2 (35 wt % in H_2O, 5.0 equiv, 39 mmol) and THF (10 mL) were added at 0 °C and then stirred for 4 h at rt. The organic layer was poured into 1 M HCl and diluted with EtOAc, then transferred to a separatory funnel. The organic layer was washed with $NaHCO_3$ (sat. aq.) and brine. The layers were separated, and the organic layer dried over $MgSO_4$, filtered, and concentrated under a vacuum. The crude product was purified by column chromatography (hexanes/EtOAc 50:50) to afford the phosphonamide as a white solid **17** (1.9 g, 49%). The solid was dissolved in hot EtOAc, hexane was added, and the flask was placed in the refrigerator (−18 °C) overnight. The resulting solid precipitant were filtered and washed with hexanes to afford the resolved product (0.375 g, 20%). ^{31}P NMR (162 MHz, $CDCl_3$) δ 9.23 (s); ^{1}H NMR (400 MHz, $CDCl_3$) δ 8.19 – 8.01 (m,

2H), 7.91 (ddd, J = 14.3, 7.7, 1.5 Hz, 1H), 7.70 (ddt, J = 8.3, 7.2, 1.2 Hz, 1H), 7.64 – 7.58 (m, 2H), 7.55 (d, J = 8.5 Hz, 2H), 7.49 (dd, J = 8.3, 6.8 Hz, 3H), 7.41 (d, J = 7.4 Hz, 1H), 7.37 – 7.32 (m, 2H), 7.26 – 7.09 (m, 5H), 7.06 – 6.97 (m, 2H), 6.71 (dt, J = 8.2, 1.2 Hz, 1H), 4.41 – 4.20 (m, 1H), 3.08 (t, J = 9.8 Hz, 1H), 1.15 (d, J = 6.8 Hz, 3H).

5-([1,1'-Biphenyl]-4-yl)-6-hydroxy-5H-dibenzo[c,e][1,2]azaphosphinine 6-sulfide (**4**)

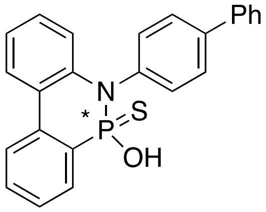

To a solution of (S_p) or (R_p)-**17** in dry THF (8 mL) was added at 0 °C NaH (3.0 equiv, 1.5 mmol, 60% dispersion in mineral oil) under argon. The reaction mixture was stirred for 1 h at rt, and then carbon disulfide (10.0 equiv, 7.7 mmol) was added dropwise and stirred for 4 h at rt. Ethyl acetate and hexanes were added and washed (3 ×) with a saturated aqueous solution of $NaHCO_3$. The two layers were separated, and the aqueous layer was acidified with 3 M HCl until pH 1 and extracted with ethyl acetate. The organic layer was dried over $MgSO_4$, filtered, and concentrated under vacuum. The crude mixture was solubilized in DCM and the precipitate filtered out. The filtrate was concentrated under a vacuum to afford the product **4** as an orange oil (0.58 g, 75%). ^{31}P NMR (162 MHz, $CDCl_3$) δ 61.8; ^{1}H NMR (400 MHz, $CDCl_3$) δ 9.34 (s, 1H), 8.30 (ddd, J = 17.0, 7.7, 1.4 Hz, 1H), 8.13 – 8.05 (m, 2H), 7.73 (dd, J = 8.1, 6.6 Hz, 3H), 7.70 – 7.63 (m, 2H), 7.58 (td, J = 7.6, 3.4 Hz, 1H), 7.52 – 7.44 (m, 4H), 7.45 – 7.37 (m, 1H), 7.28 – 7.25 (m, 1H), 7.20 (t, J = 7.5 Hz, 1H), 6.78 (d, J = 8.2 Hz, 1H).

References:

[1] Bryant, D.; Kilner, C.; Kee, T. *Inorg. Chim. Acta.* **2009**, *362*, 614.

[2] Riedmüller, S.; Kaufhold, O.; Spreitzer, H.; Nachtsheim, B. J. *Eur. J. Org. Chem.* **2014**, 1391–1394.

[3] Ferry, F.; Stemper, J.; Marinetti, A.; Voituriez, A.; Guinchard, X. *Eur. J. Org. Chem.,* **2014**, 188–193. DOI: 10.1002/ejoc.201301253.

HPLC chromatograms.

Representative procedure for the asymmetric hydrogenation of 2-phenylquinoline (**20**)

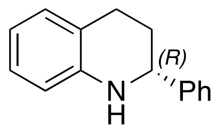

To a reaction tube was added 2-phenylquinoline (1 equiv, 0.25 mmol), the Hantzsch ester **19** (2.4 equiv, 0.58 mmol) in toluene (5 mL) under argon. The reaction mixture was cooled to 0 °C, then the CPA catalyst **2–4** (0.005 mmol, 2 mol %) was added. The reaction mixture was brought to rt and stirred for 24 h. The reaction mixture was concentrated under vacuum, and the crude product was purified directly by column chromatography (hexanes/ethyl acetate 95:5) to yield known compound **20,** as a colorless oil in quantitative yield.[3] Enantiomeric excess was determined by HPLC with a Chiralcel OD-H column (hexane/iPrOH 95:5, 1.0 mL/min).

Racemic 2-phenyl-1,2,3,4-tetra-hydroquinoline (**20**) *HPLC.*

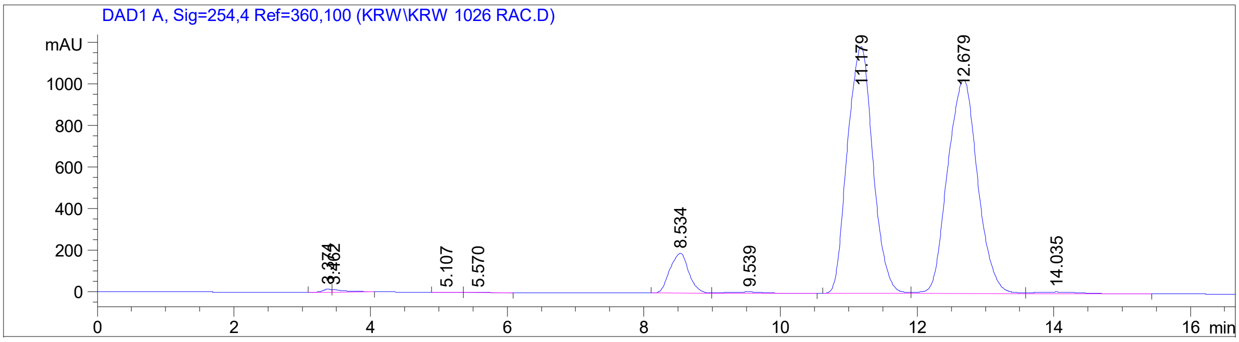

Signal 1: DAD1 A, Sig=254,4 Ref=360,100

Peak #	RetTime [min]	Type	Width [min]	Area [mAU*s]	Height [mAU]	Area %
1	3.374	BV	0.1217	107.46756	13.67281	0.1607
2	3.462	VB	0.1805	162.36674	11.80467	0.2428
3	5.107	BV	0.1814	17.68106	1.47967	0.0264
4	5.570	VB	0.1998	16.04880	1.21773	0.0240
5	8.534	BV	0.3105	3673.62305	192.60971	5.4927
6	9.539	VB	0.4760	226.74150	6.26831	0.3390
7	11.179	BV	0.4184	3.08269e4	1189.19141	46.0919
8	12.679	VB	0.4916	3.14381e4	1031.29443	47.0057
9	14.035	BB	0.5761	412.46259	8.56110	0.6167

Totals : 6.68814e4 2456.09984

*2-Phenyl-1,2,3,4-tetra-hydroquinoline from CPA **2** HPLC*

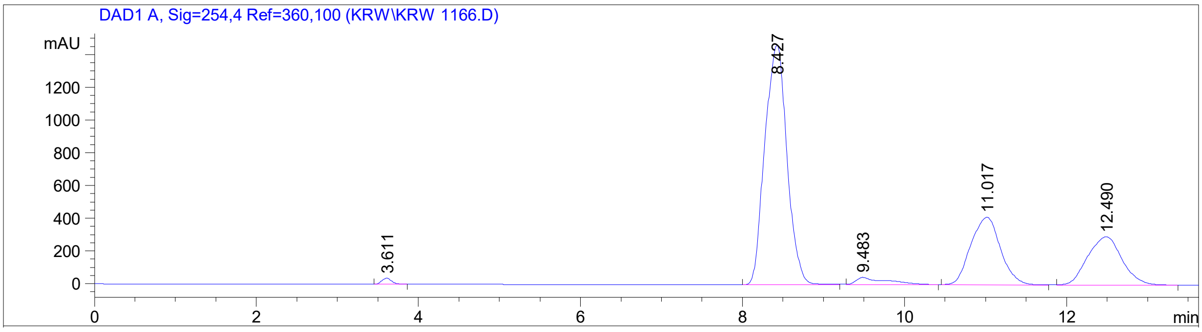

Signal 1: DAD1 A, Sig=254,4 Ref=360,100

Peak #	RetTime [min]	Type	Width [min]	Area [mAU*s]	Height [mAU]	Area %
1	3.611	BB	0.1295	311.49915	38.06655	0.6414
2	8.427	BB	0.3080	2.78476e4	1463.50867	57.3414
3	9.483	BB	0.3455	1234.98560	45.95943	2.5430
4	11.017	BB	0.4118	1.05513e4	413.30026	21.7262
5	12.490	BB	0.4731	8619.25293	294.80676	17.7480
Totals :				4.85646e4	2255.64167	

*2-Phenyl-1,2,3,4-tetrahydroquinoline from CPA **3** HPLC.*

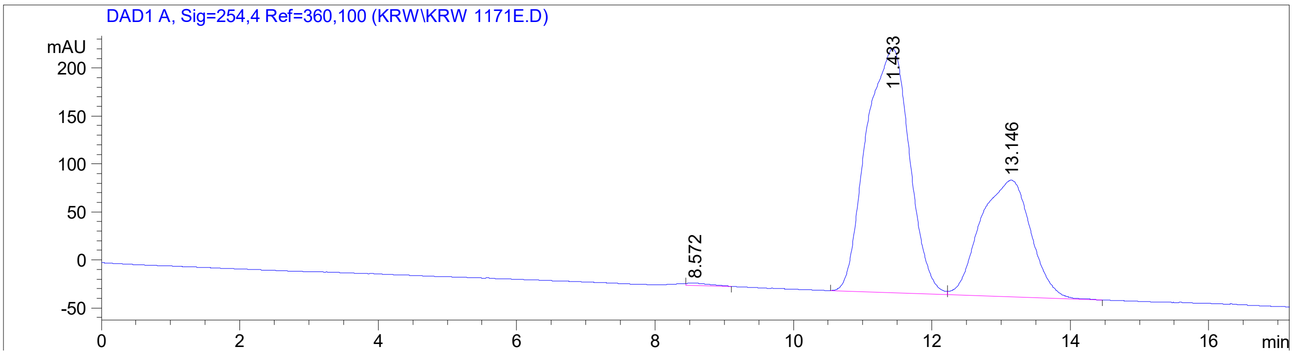

Signal 1: DAD1 A, Sig=254,4 Ref=360,100

Peak #	RetTime [min]	Type	Width [min]	Area [mAU*s]	Height [mAU]	Area %
1	8.572	BB	0.3008	65.14135	2.63140	0.3780
2	11.433	BV	0.5957	1.10346e4	254.57335	64.0239
3	13.146	VB	0.6986	6135.39111	121.60060	35.5982

Totals : 1.72351e4 378.80535

*2-Phenyl-1,2,3,4-tetrahydroquinoline from CPA **4** HPLC:*

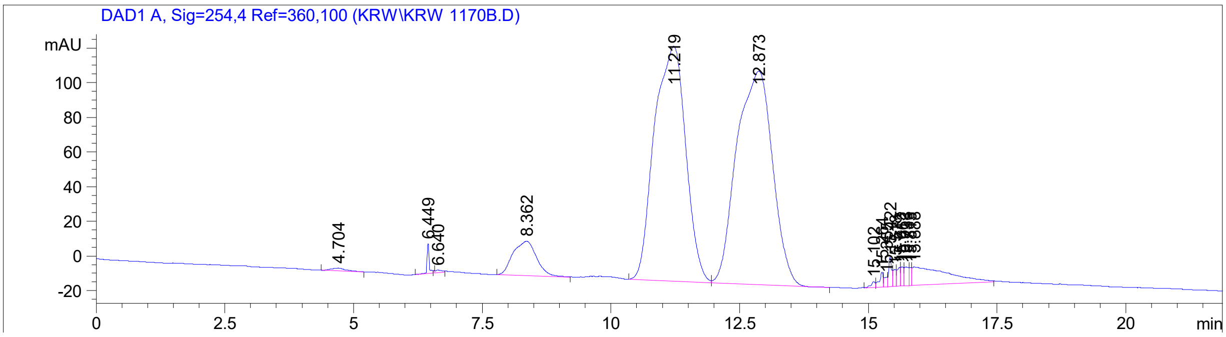

Signal 1: DAD1 A, Sig=254,4 Ref=360,100

Peak #	RetTime [min]	Type	Width [min]	Area [mAU*s]	Height [mAU]	Area %
1	4.704	BB	0.2404	29.89528	1.54059	0.2247
2	6.449	BB	0.0466	50.97886	17.19250	0.3832
3	6.640	BB	0.1065	12.50844	1.54424	0.0940
4	8.362	BB	0.4317	627.11212	19.89706	4.7134
5	11.219	BV	0.5768	5721.33643	135.43665	43.0020
6	12.873	VB	0.6832	5966.75293	123.16451	44.8466
7	15.102	VV	0.0800	19.11582	3.37819	0.1437
8	15.264	VV	0.0691	43.87333	8.66000	0.3298
9	15.355	VV	0.0607	26.47861	5.65116	0.1990
10	15.422	VV	0.0582	67.97022	17.16884	0.5109
11	15.518	VV	0.0551	39.45806	9.77429	0.2966
12	15.613	VV	0.0622	50.56691	10.89502	0.3801
13	15.662	VV	0.0565	41.87500	10.97777	0.3147
14	15.733	VV	0.0802	61.59865	10.85541	0.4630
15	15.813	VV	0.0508	34.66135	10.41127	0.2605
16	15.888	VB	0.5615	510.62433	10.80262	3.8379

Totals : 1.33048e4 397.35012

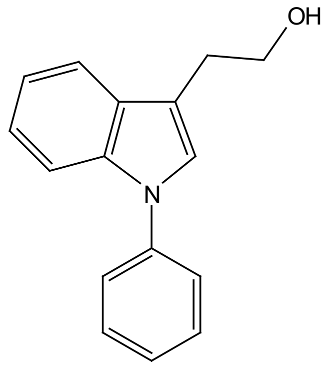
OH
N

6

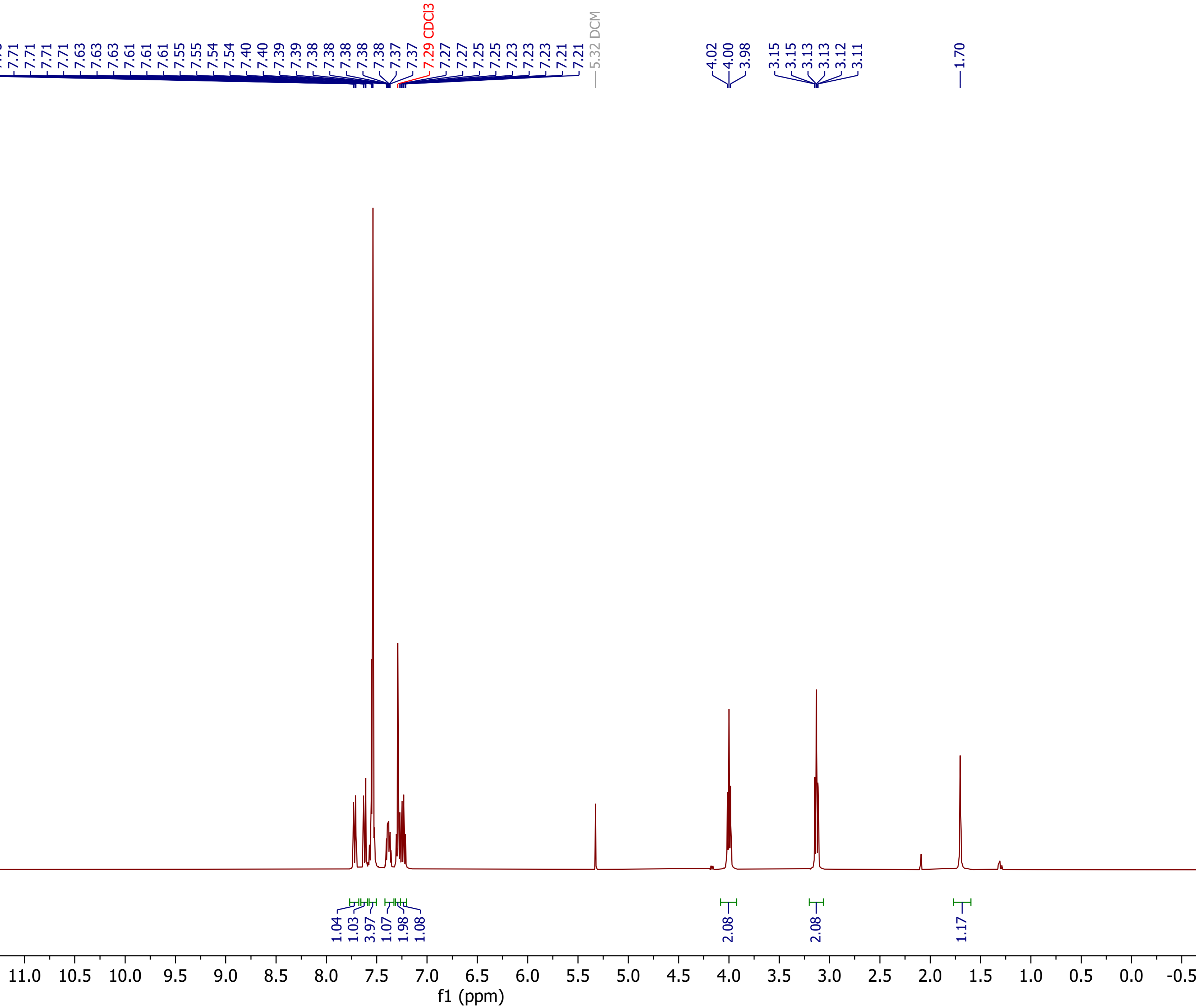
7.73
7.73
7.73
7.73
7.71
7.71
7.71
7.71
7.63
7.63
7.63
7.61
7.61
7.61
7.55
7.55
7.54
7.54
7.40
7.40
7.39
7.39
7.38
7.38
7.38
7.38
7.38
7.37
7.37
7.29 CDCl3
7.27
7.27
7.25
7.25
7.23
7.23
7.23
7.21
7.21
5.32 DCM
4.02
4.00
3.98
3.15
3.15
3.13
3.13
3.12
3.11
1.70
1.04
1.03
3.97
1.07
1.98
1.08
2.08
2.08
1.17
13.5
13.0
12.5
12.0
11.5
11.0
10.5
10.0
9.5
9.0
8.5
8.0
7.5
7.0
6.5
6.0
5.5
5.0
4.5
4.0
3.5
3.0
2.5
2.0
1.5
1.0
0.5
0.0
-0.5
f1 (ppm)

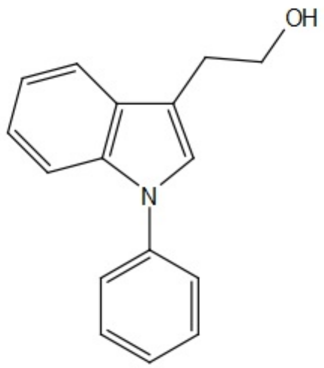
OH
N

6

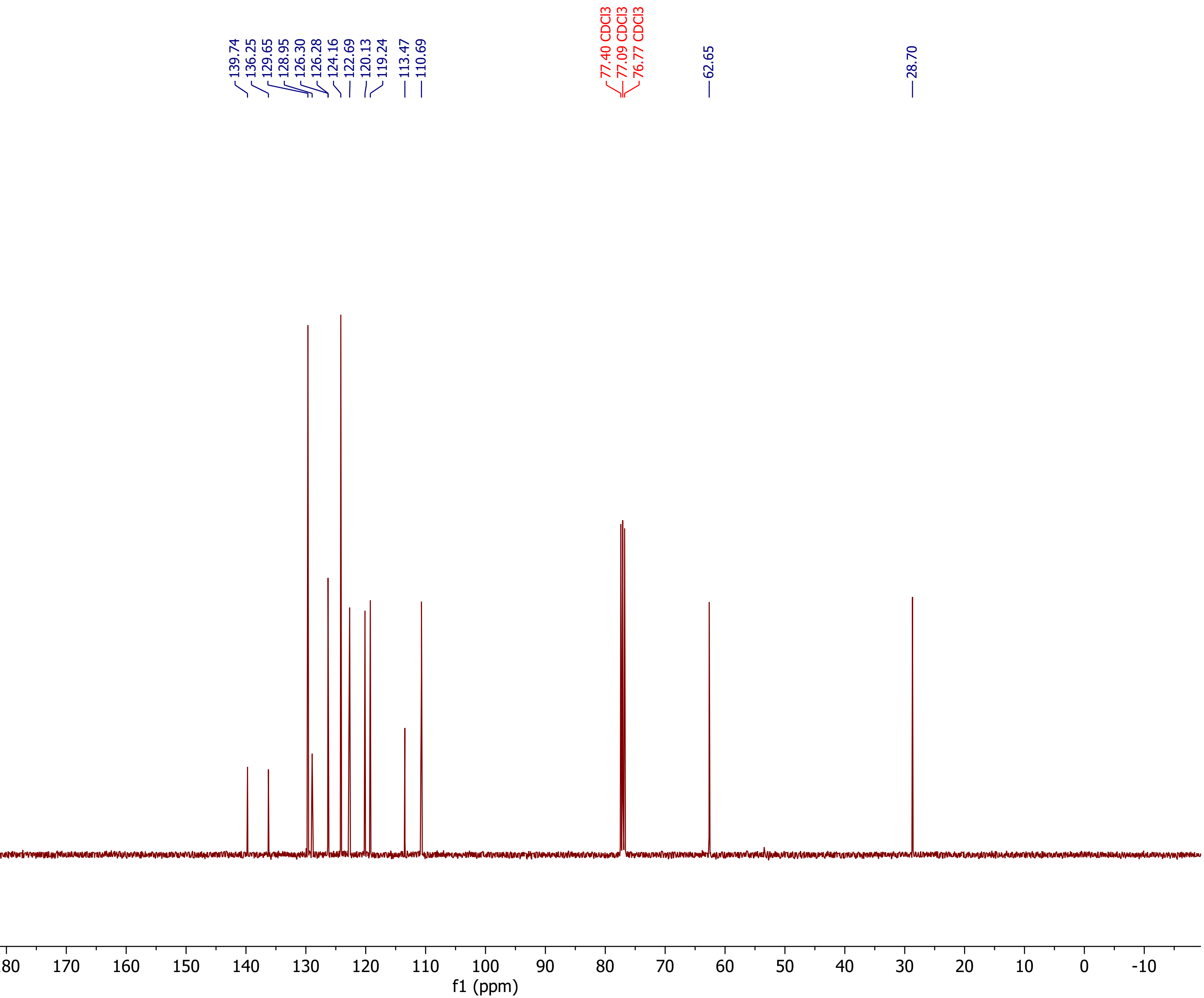
139.74
136.25
129.65
128.95
126.30
126.28
124.16
122.69
120.13
119.24
113.47
110.69
77.40 CDCl3
77.09 CDCl3
76.77 CDCl3
62.65
28.70
210
200
190
180
170
160
150
140
130
120
110
100
90
80
70
60
50
40
30
20
10
0
-10
f1 (ppm)

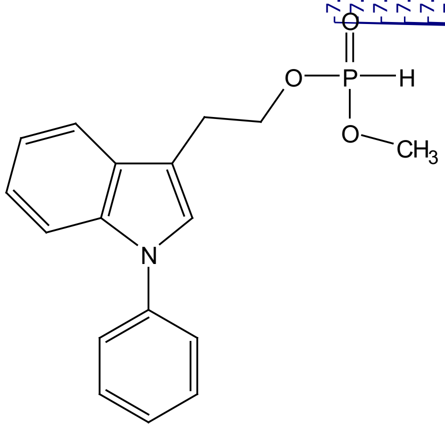
O
O
P
H
O
CH3
N

7

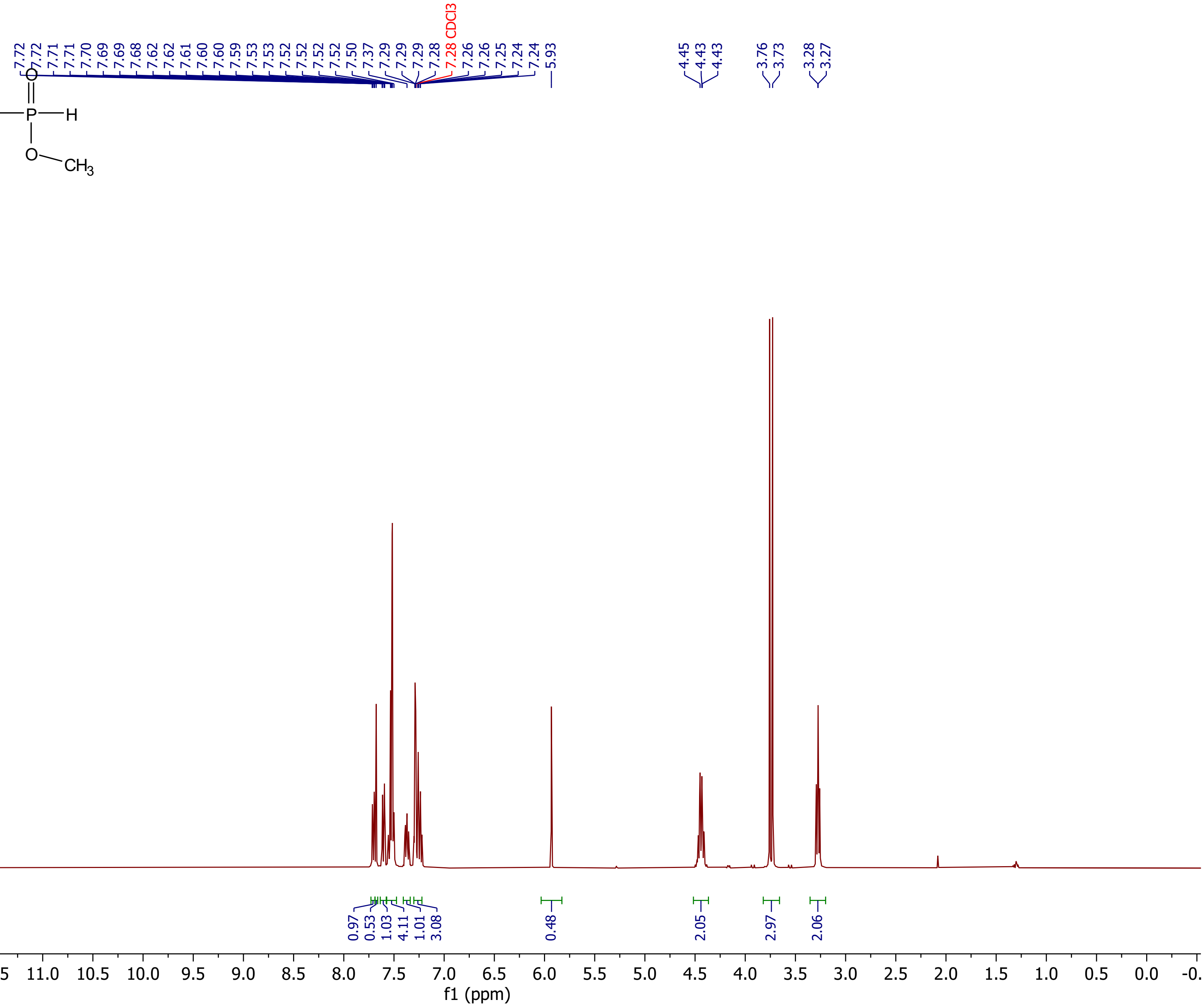
7.72
7.72
7.71
7.71
7.70
7.69
7.69
7.68
7.62
7.62
7.61
7.60
7.60
7.59
7.53
7.53
7.52
7.52
7.52
7.52
7.50
7.37
7.29
7.29
7.29
7.28
7.28 CDCl3
7.26
7.26
7.25
7.24
7.24
5.93
4.45
4.43
4.43
3.76
3.73
3.28
3.27
0.97
0.53
1.03
4.11
1.01
3.08
0.48
2.05
2.97
2.06
13.5 13.0 12.5 12.0 11.5 11.0 10.5 10.0 9.5 9.0 8.5 8.0 7.5 7.0 6.5 6.0 5.5 5.0 4.5 4.0 3.5 3.0 2.5 2.0 1.5 1.0 0.5 0.0 -0.
f1 (ppm)

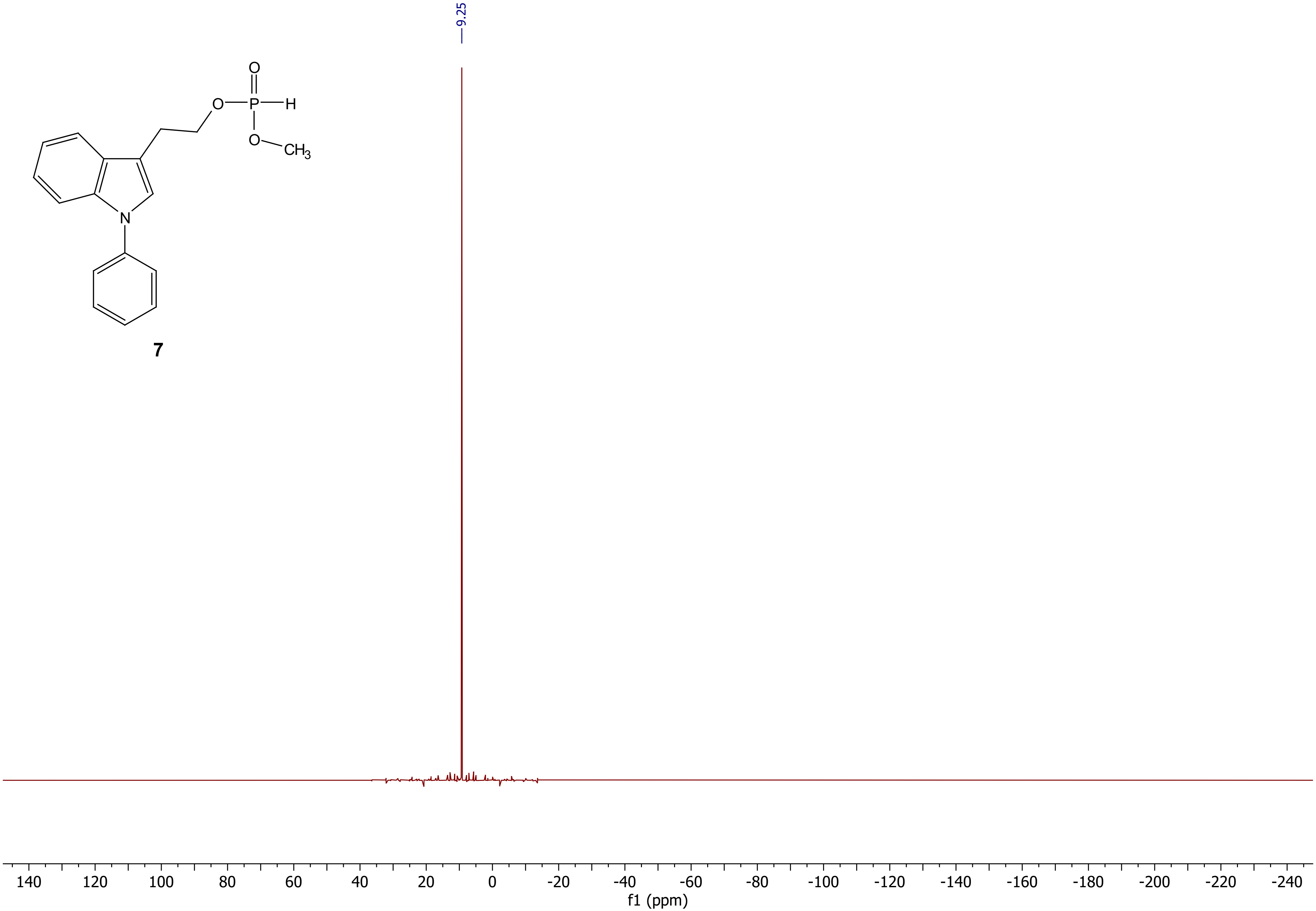
O
O
P
H
O
CH3
N
9.25
140
120
100
80
60
40
20
0
-20
-40
-60
-80
-100
-120
-140
-160
-180
-200
-220
-240
f1 (ppm)

7

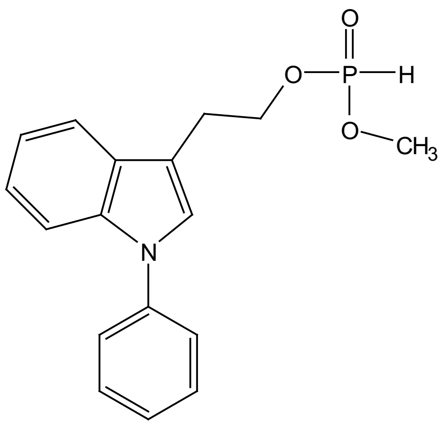
O
O
P
H
O
CH3
N

7

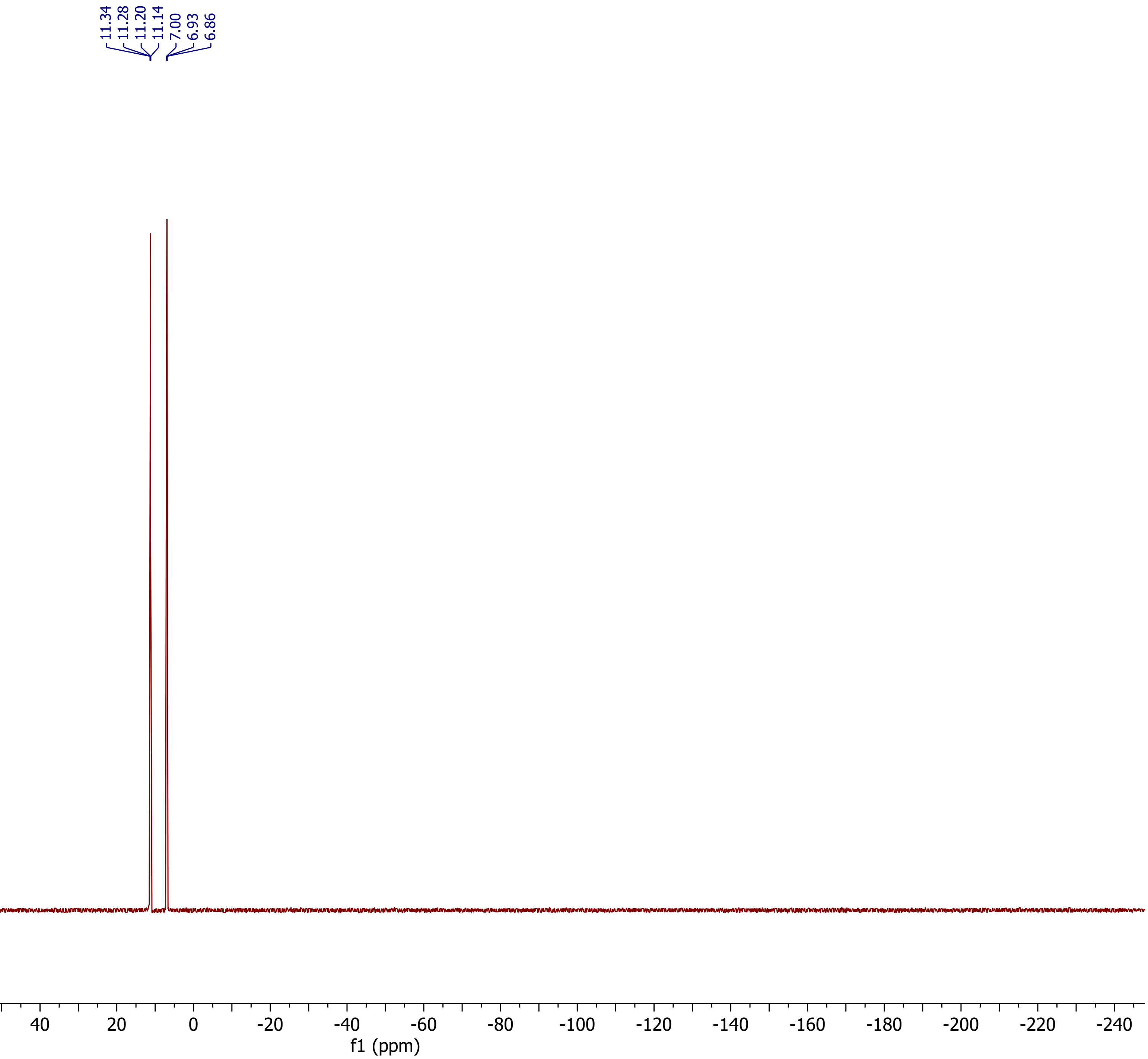
11.34
11.28
11.20
11.14
7.00
6.93
6.86
140
120
100
80
60
40
20
0
-20
-40
-60
-80
-100
-120
-140
-160
-180
-200
-220
-240
f1 (ppm)

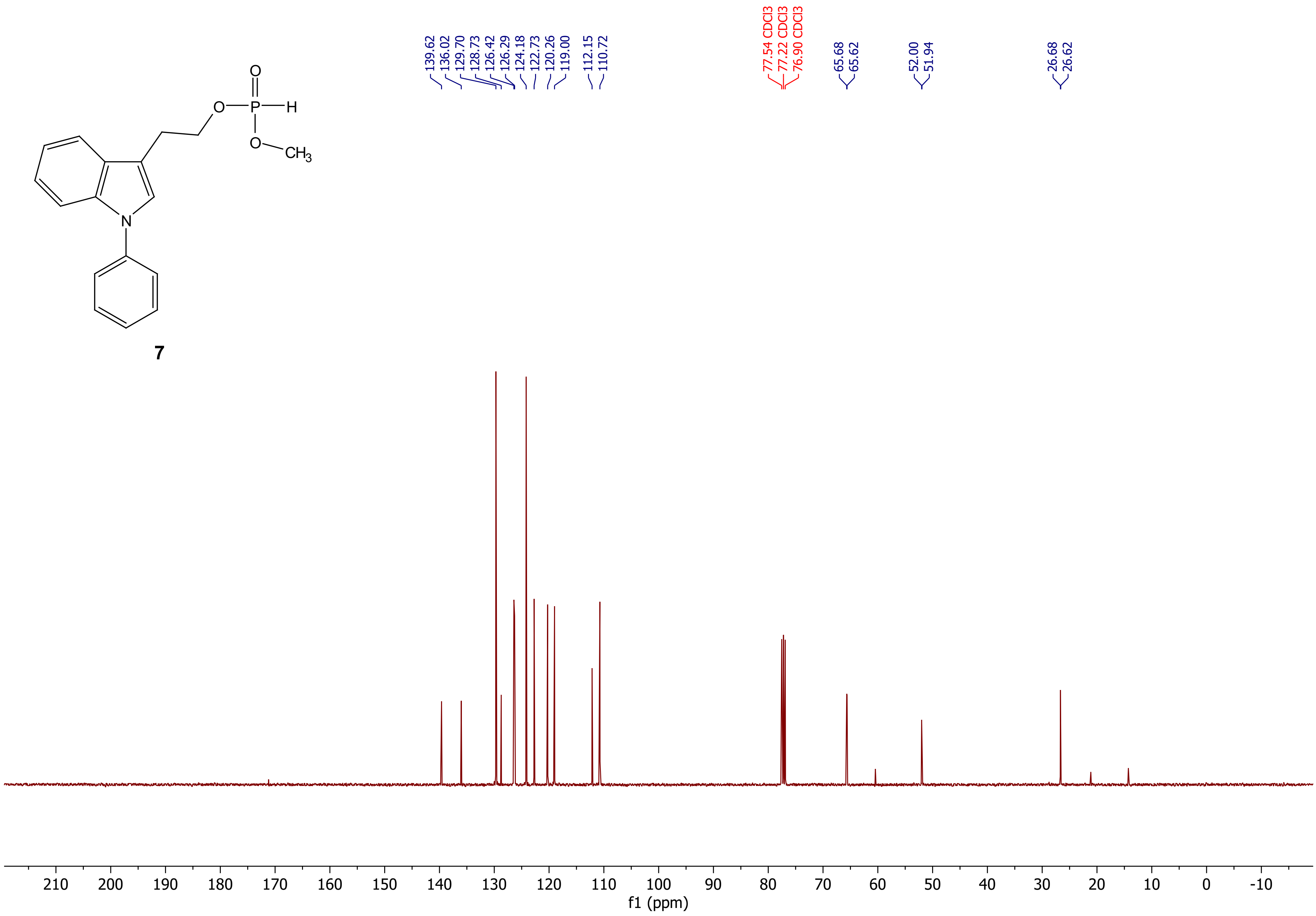

O
O—P—H
O—CH3
N
7
139.62
136.02
129.70
128.73
126.42
126.29
124.18
122.73
120.26
119.00
112.15
110.72
77.54 CDCl3
77.22 CDCl3
76.90 CDCl3
65.68
65.62
52.00
51.94
26.68
26.62
210 200 190 180 170 160 150 140 130 120 110 100 90 80 70 60 50 40 30 20 10 0 -10
f1 (ppm)

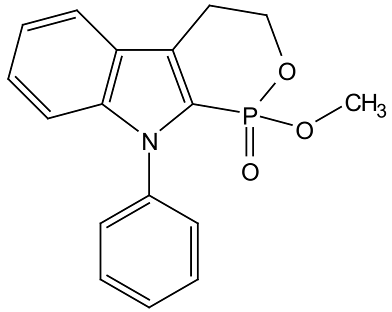

O
P
O
CH3
N
O

8

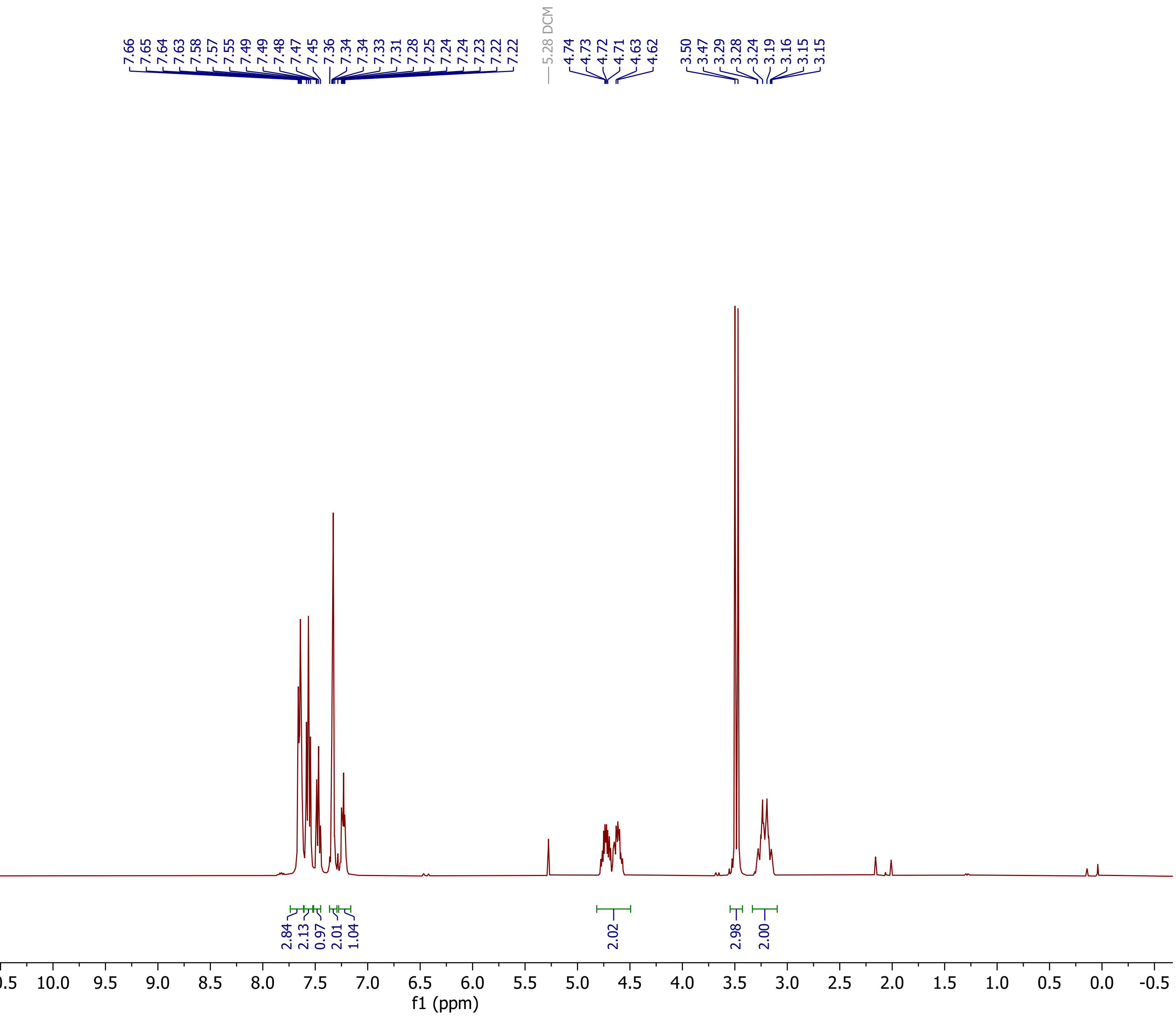

7.66 7.65 7.64 7.63 7.58 7.57 7.55 7.49 7.49 7.48 7.47 7.45 7.36 7.34 7.34 7.33 7.31 7.28 7.25 7.24 7.24 7.23 7.22 7.22
5.28 DCM
4.74 4.73 4.72 4.71 4.63 4.62
3.50 3.47 3.29 3.28 3.24 3.19 3.16 3.15 3.15
2.84 2.13 0.97 2.01 1.04
2.02
2.98 2.00
13.0 12.5 12.0 11.5 11.0 10.5 10.0 9.5 9.0 8.5 8.0 7.5 7.0 6.5 6.0 5.5 5.0 4.5 4.0 3.5 3.0 2.5 2.0 1.5 1.0 0.5 0.0 -0.5
f1 (ppm)

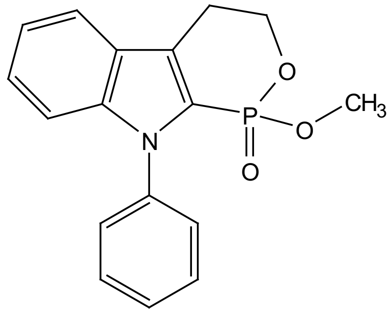

8

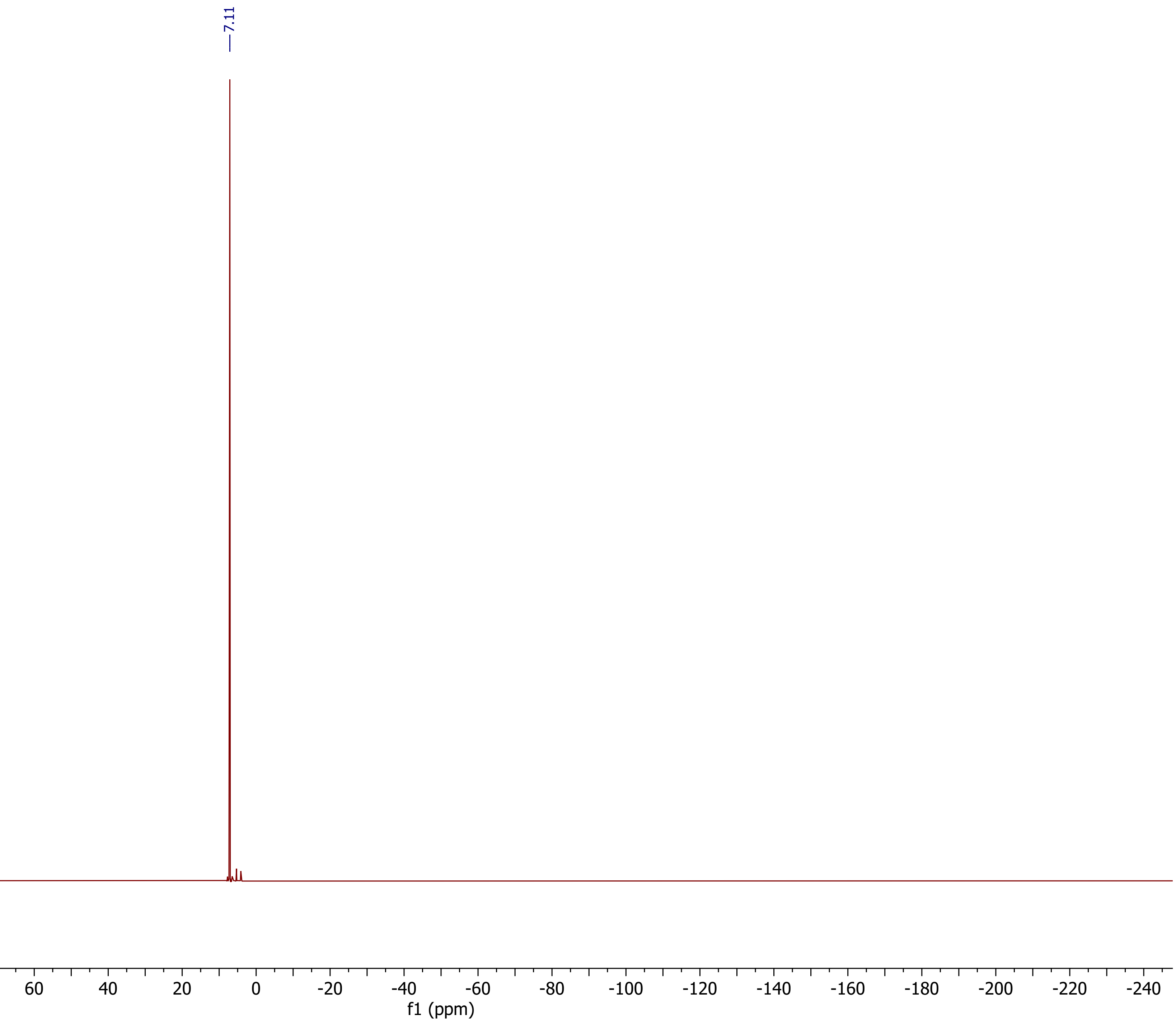

7.11
140
120
100
80
60
40
20
0
-20
-40
-60
-80
-100
-120
-140
-160
-180
-200
-220
-240
f1 (ppm)

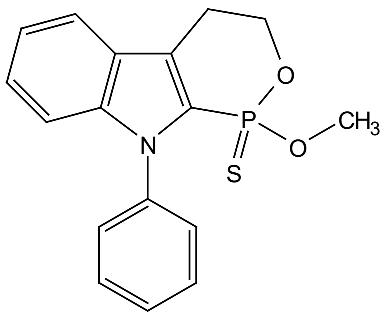

9

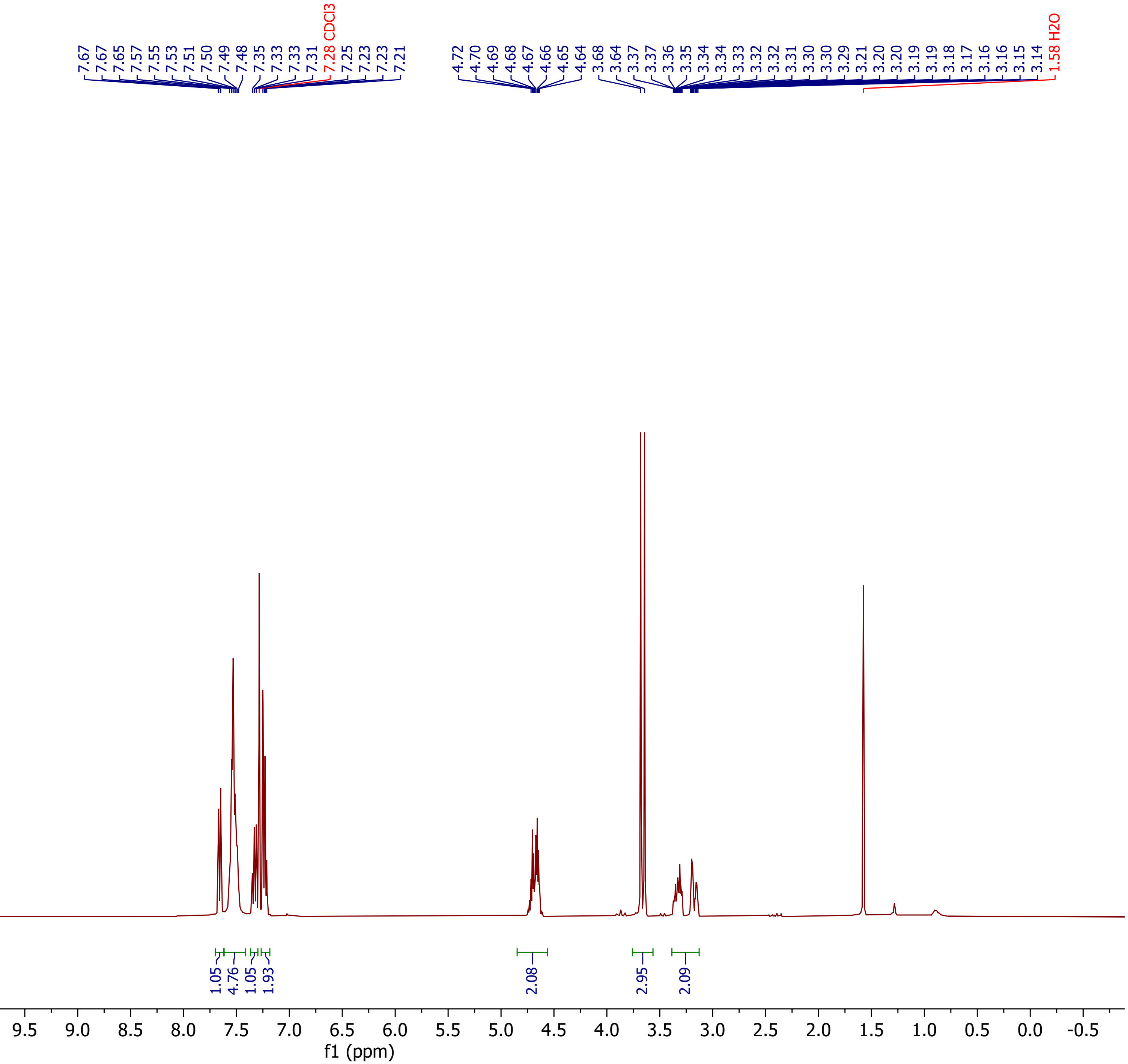

7.67
7.67
7.65
7.57
7.55
7.53
7.51
7.50
7.49
7.48
7.35
7.33
7.33
7.31
7.28 CDCl3
7.25
7.23
7.23
7.21
4.72
4.70
4.69
4.68
4.67
4.66
4.65
4.64
3.68
3.64
3.37
3.37
3.36
3.35
3.34
3.34
3.33
3.32
3.32
3.31
3.30
3.30
3.29
3.21
3.20
3.20
3.19
3.19
3.18
3.17
3.16
3.16
3.15
3.14
1.58 H2O
1.05
4.76
1.05
1.93
2.08
2.95
2.09
f1 (ppm)

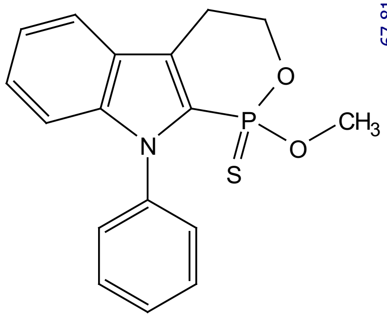
O
P
O
CH3
N
S

9

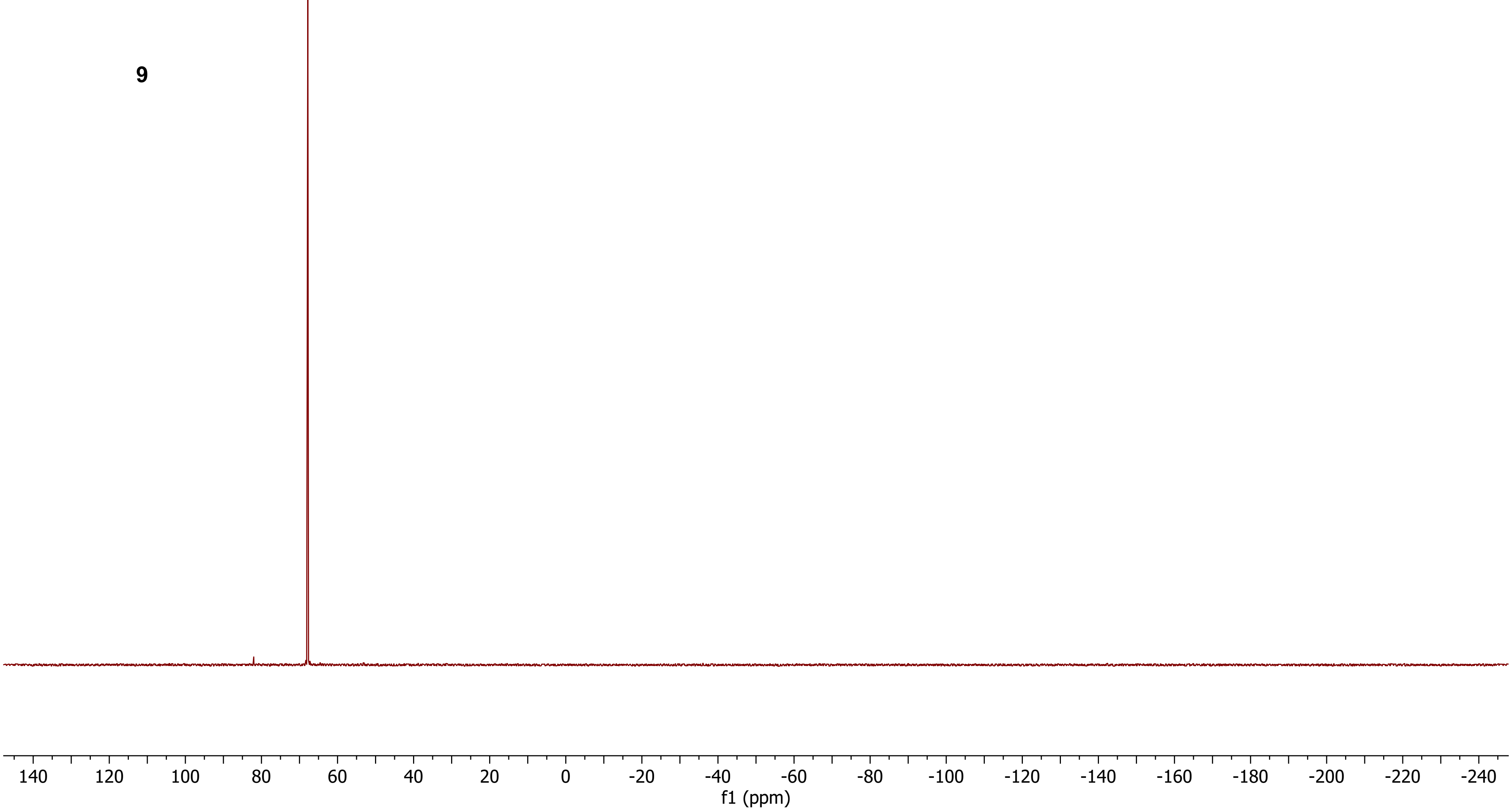
67.81
67.78
140 120 100 80 60 40 20 0 -20 -40 -60 -80 -100 -120 -140 -160 -180 -200 -220 -240
f1 (ppm)

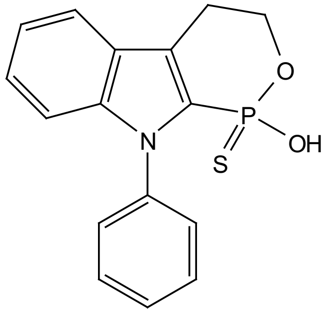

1

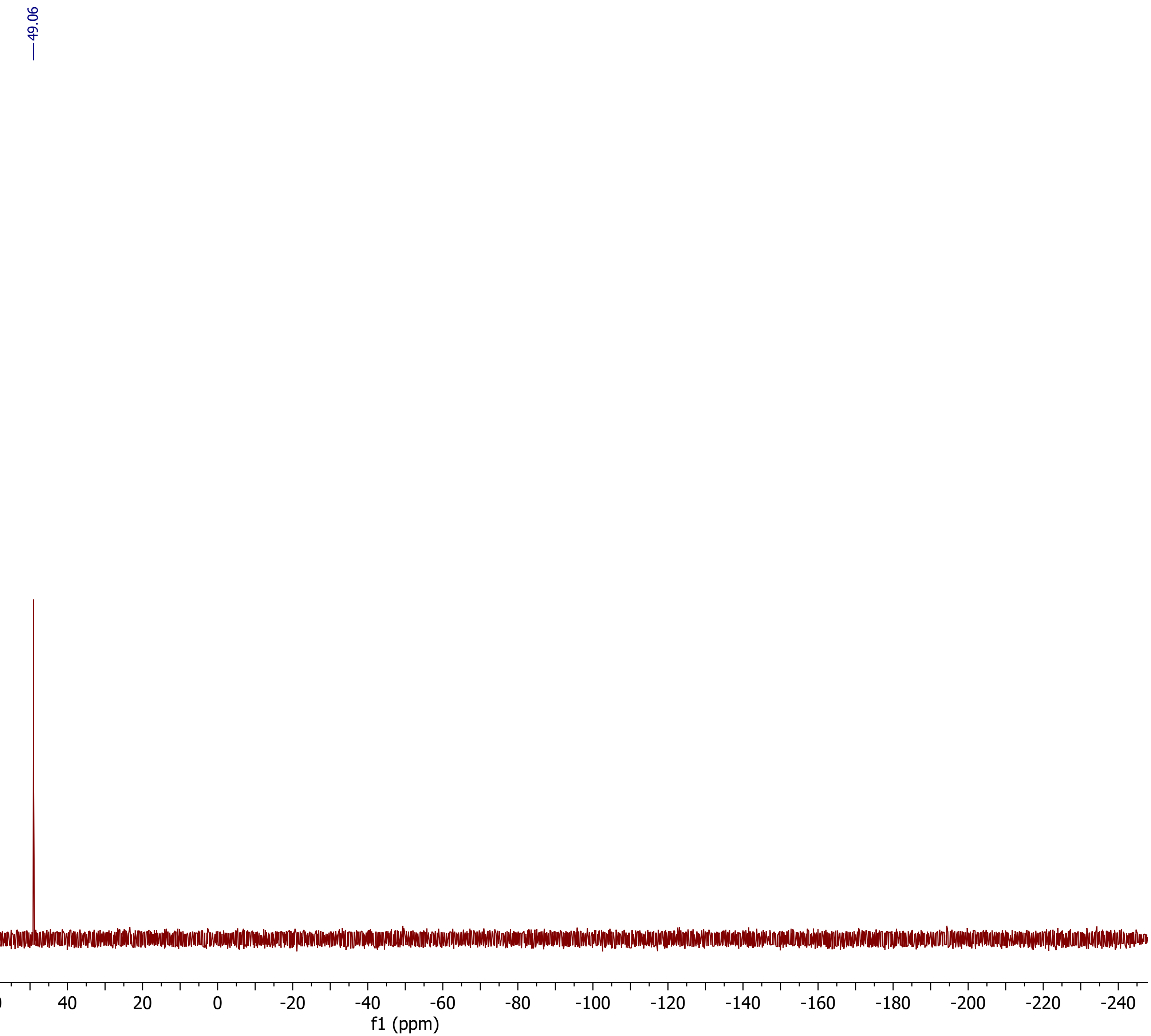

49.06
140
120
100
80
60
40
20
0
-20
-40
-60
-80
-100
-120
-140
-160
-180
-200
-220
-240
f1 (ppm)

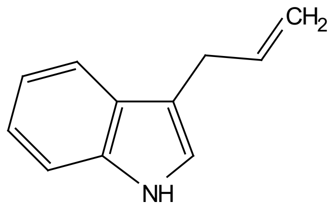
CH2
NH

10

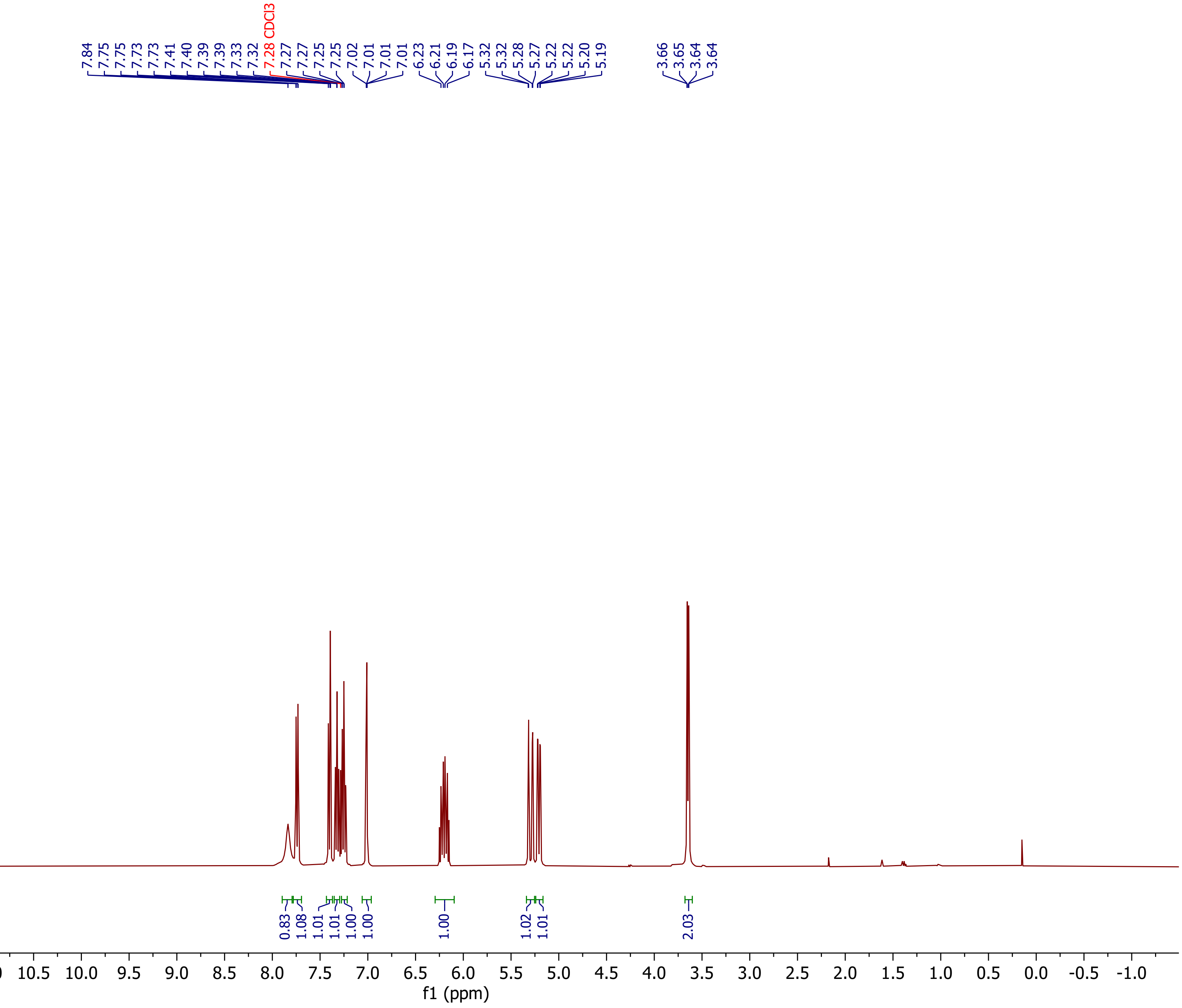
7.84
7.75
7.75
7.73
7.73
7.41
7.40
7.39
7.39
7.33
7.32
7.28 CDCl3
7.27
7.27
7.25
7.25
7.02
7.01
7.01
7.01
6.23
6.21
6.19
6.17
5.32
5.32
5.28
5.27
5.22
5.22
5.20
5.19
3.66
3.65
3.64
3.64
0.83
1.08
1.01
1.01
1.00
1.00
1.00
1.02
1.01
2.03
13.5 13.0 12.5 12.0 11.5 11.0 10.5 10.0 9.5 9.0 8.5 8.0 7.5 7.0 6.5 6.0 5.5 5.0 4.5 4.0 3.5 3.0 2.5 2.0 1.5 1.0 0.5 0.0 -0.5 -1.0
f1 (ppm)

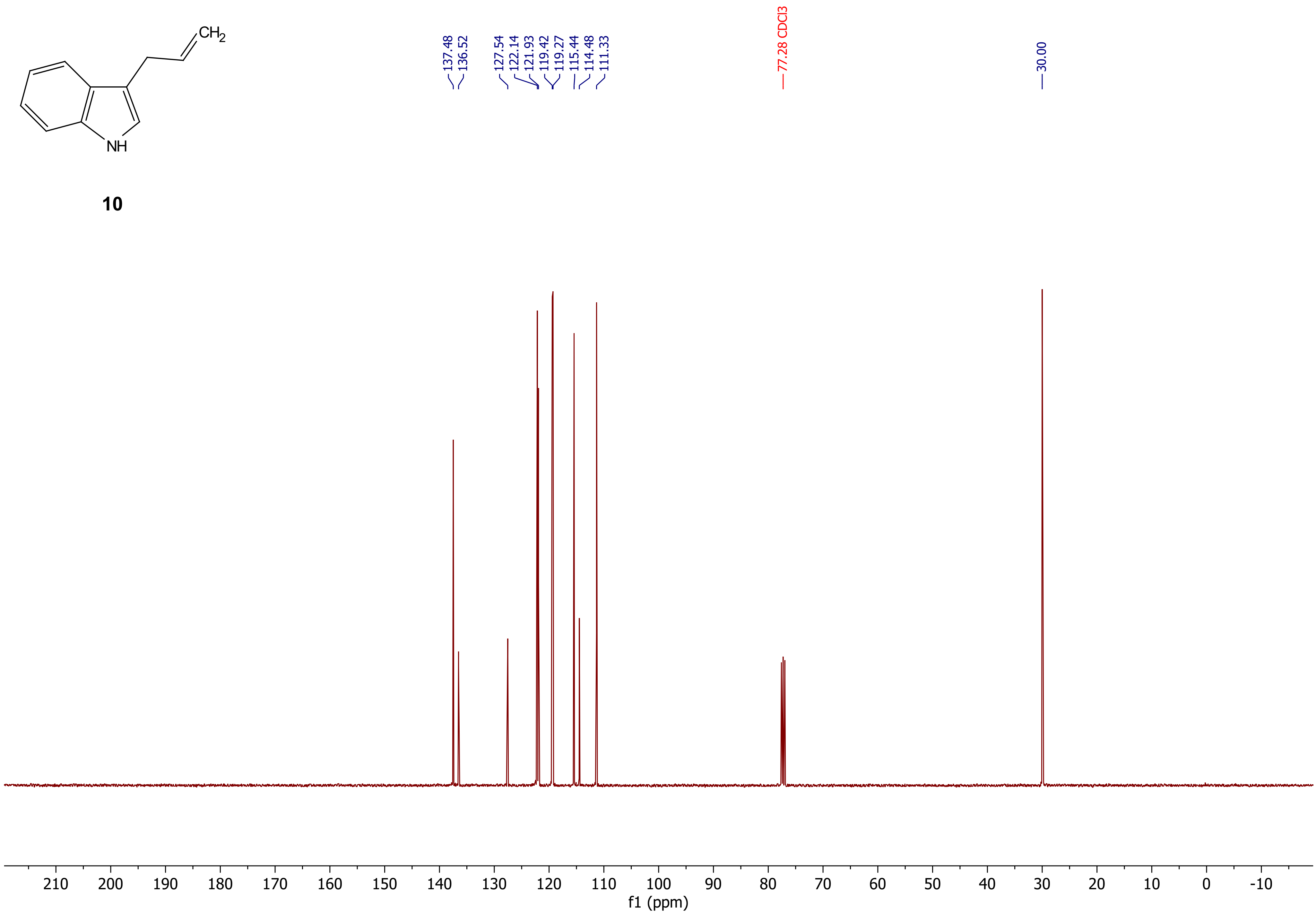
CH2
NH
10
137.48
136.52
127.54
122.14
121.93
119.42
119.27
115.44
114.48
111.33
77.28 CDCl3
30.00
210 200 190 180 170 160 150 140 130 120 110 100 90 80 70 60 50 40 30 20 10 0 -10
f1 (ppm)

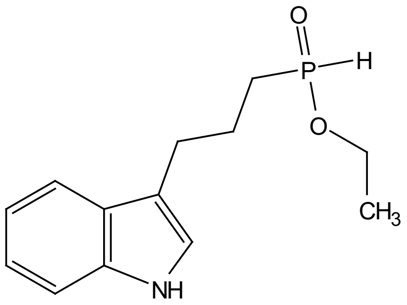

11

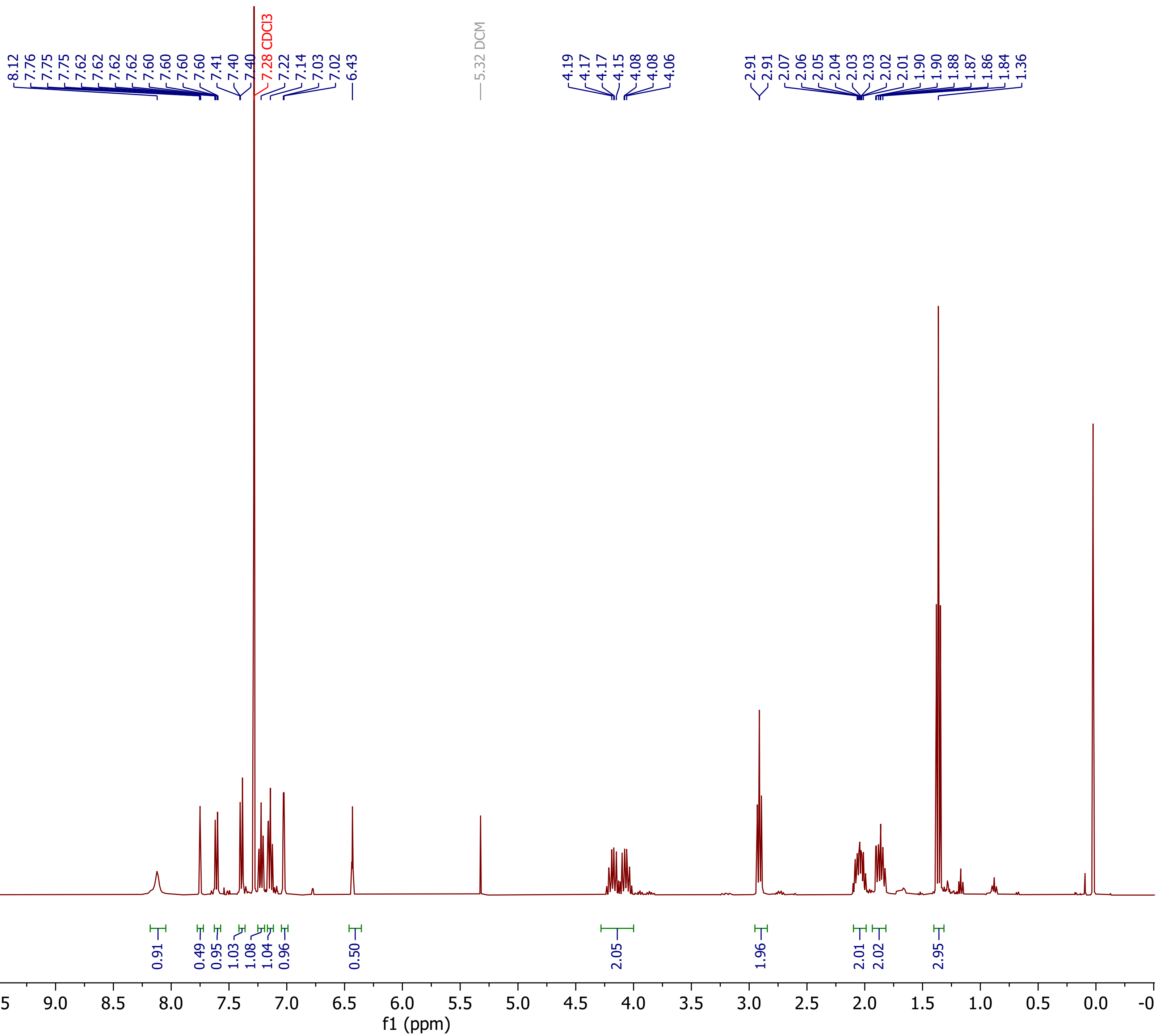

8.12 7.76 7.75 7.75 7.62 7.62 7.62 7.62 7.60 7.60 7.60 7.60 7.41 7.40 7.40
7.28 CDCl3
7.22 7.14 7.03 7.02 6.43
5.32 DCM
4.19 4.17 4.17 4.15 4.08 4.08 4.06
2.91 2.91 2.07 2.06 2.05 2.04 2.03 2.03 2.02 2.01 1.90 1.90 1.88 1.87 1.86 1.84 1.36
0.91 0.49 0.95 1.03 1.08 1.04 0.96 0.50 2.05 1.96 2.01 2.02 2.95
12.0 11.5 11.0 10.5 10.0 9.5 9.0 8.5 8.0 7.5 7.0 6.5 6.0 5.5 5.0 4.5 4.0 3.5 3.0 2.5 2.0 1.5 1.0 0.5 0.0 -0
f1 (ppm)

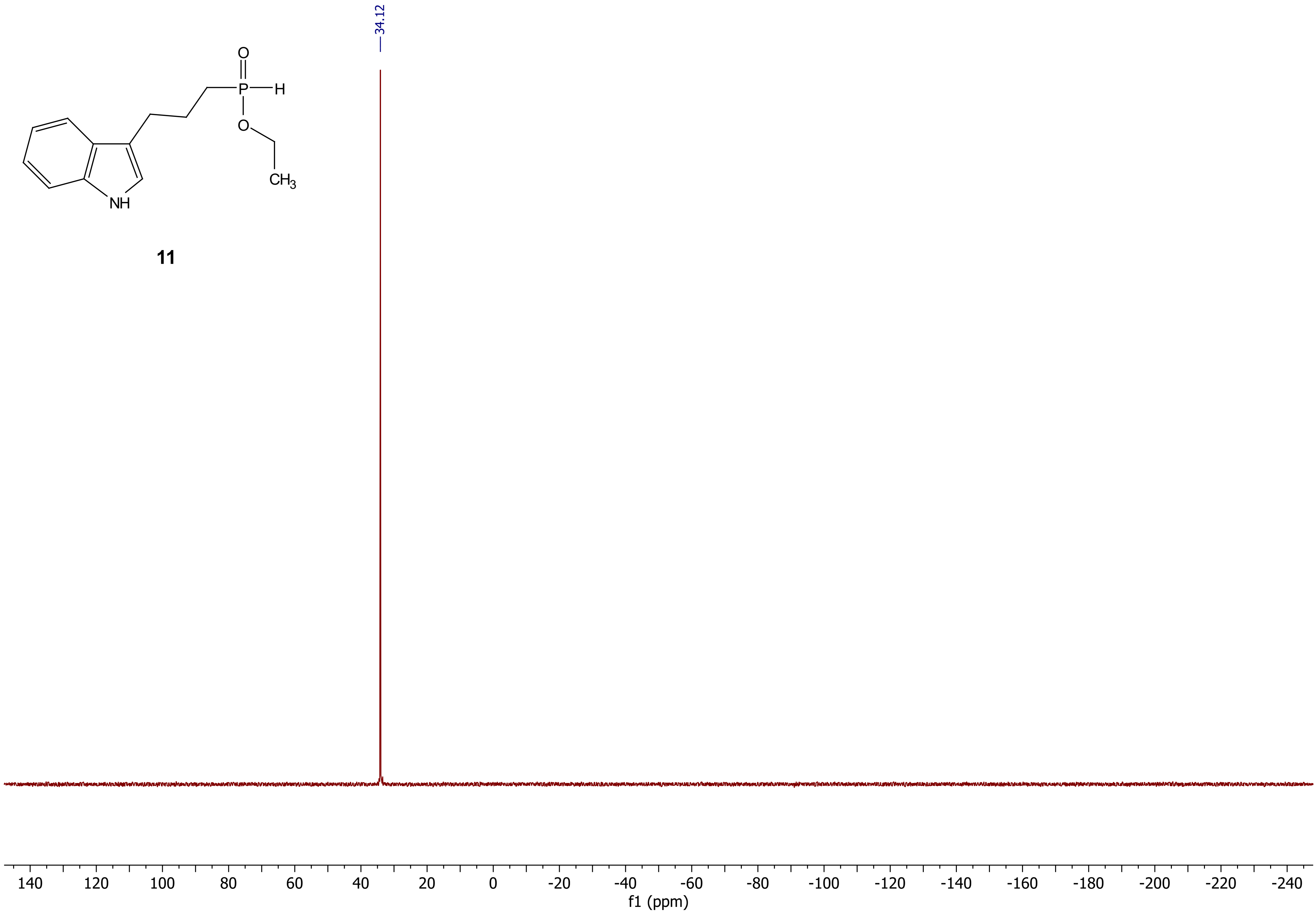
O
P
H
O
CH3
NH
34.12
140
120
100
80
60
40
20
0
-20
-40
-60
-80
-100
-120
-140
-160
-180
-200
-220
-240
f1 (ppm)

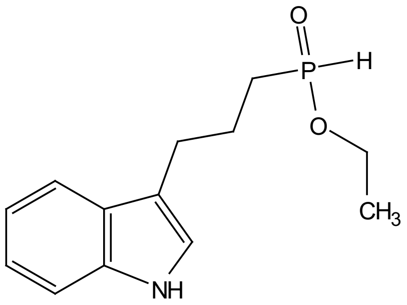
O
P
H
O
CH3
NH

11

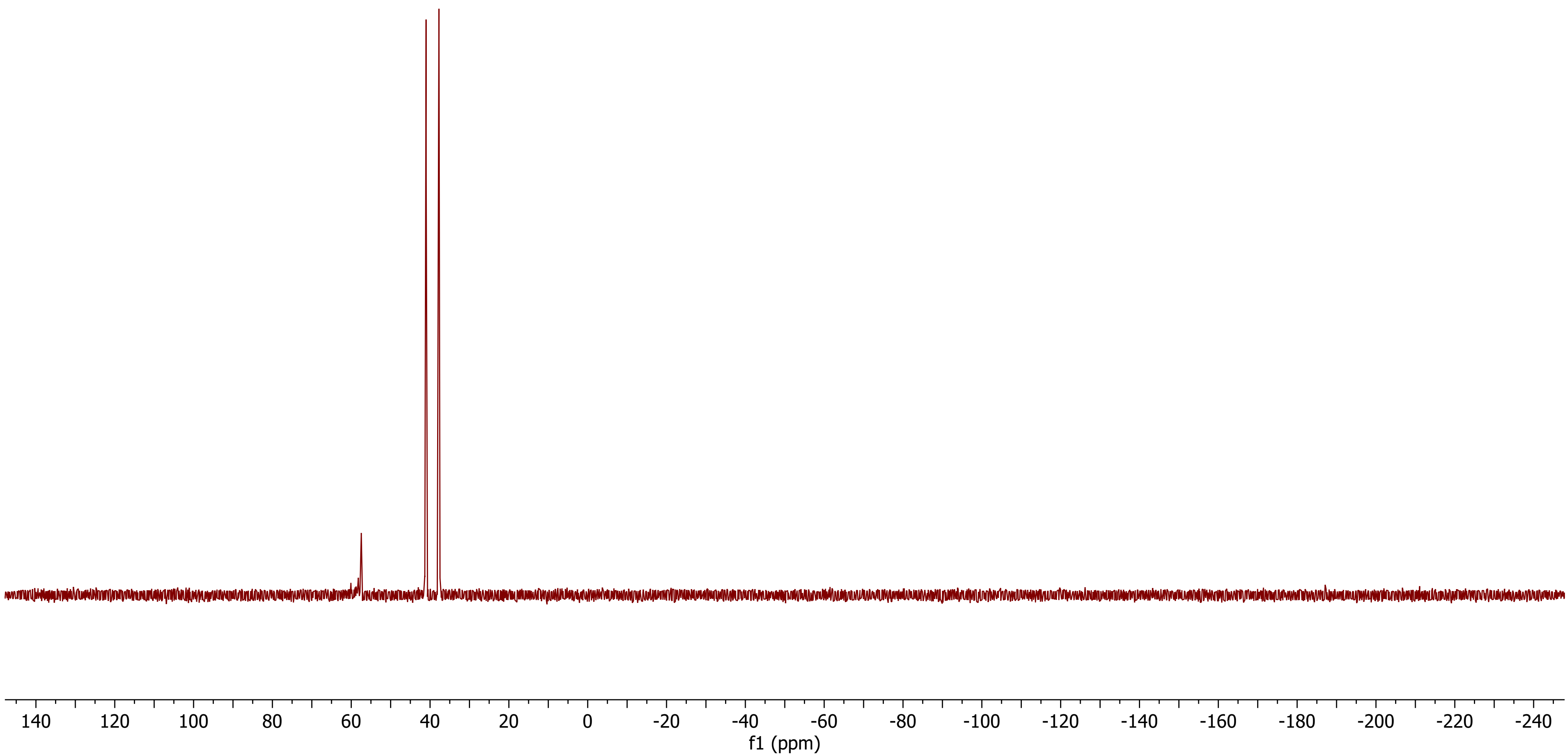
41.00
37.74
140
120
100
80
60
40
20
0
-20
-40
-60
-80
-100
-120
-140
-160
-180
-200
-220
-240
f1 (ppm)

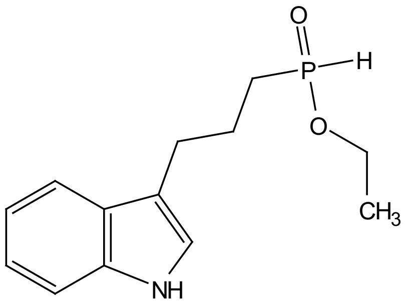

11

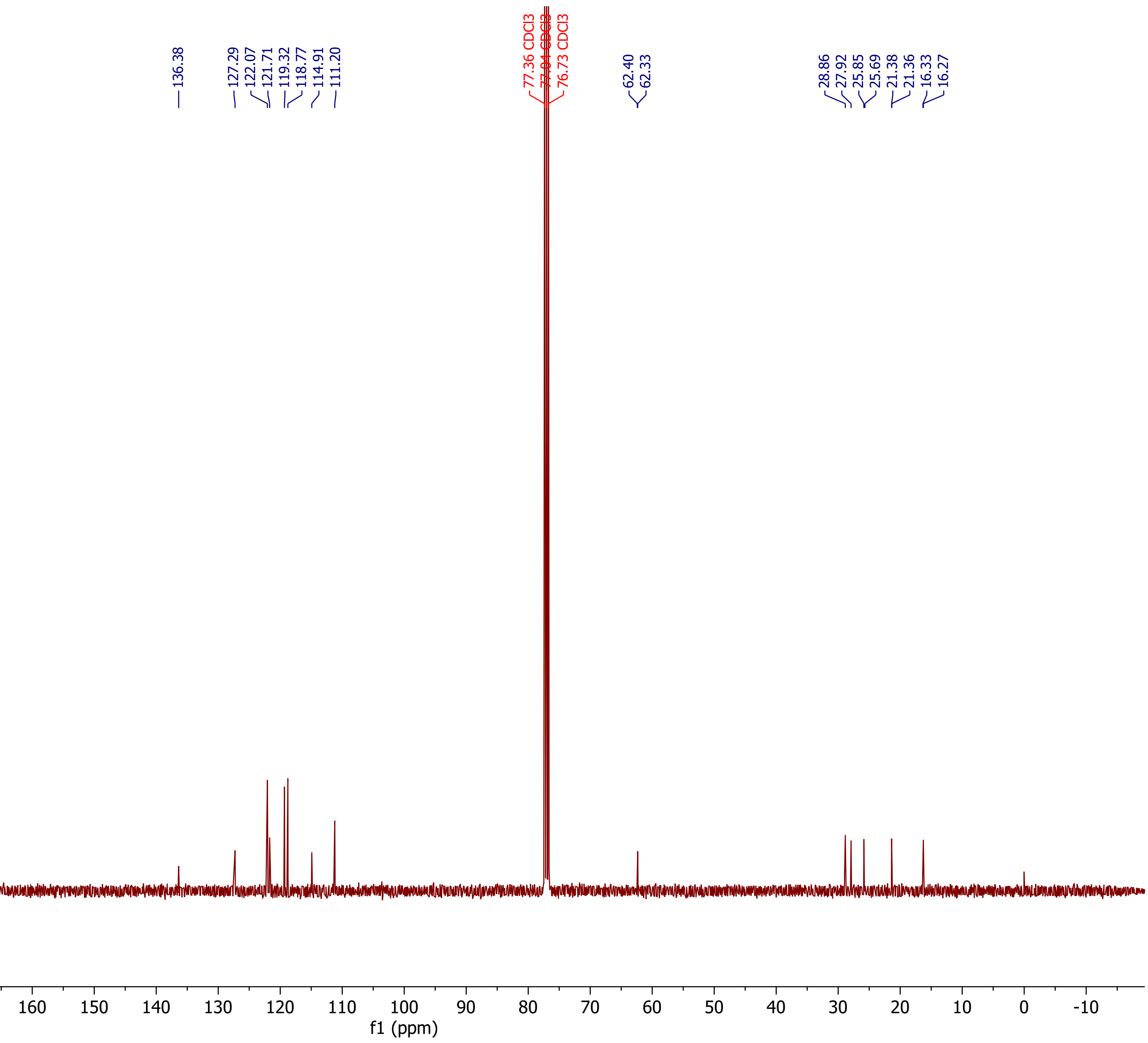

136.38
127.29
122.07
121.71
119.32
118.77
114.91
111.20
77.36 CDCl3
77.04 CDCl3
76.73 CDCl3
62.40
62.33
28.86
27.92
25.85
25.69
21.38
21.36
16.33
16.27
210 200 190 180 170 160 150 140 130 120 110 100 90 80 70 60 50 40 30 20 10 0 -10
f1 (ppm)

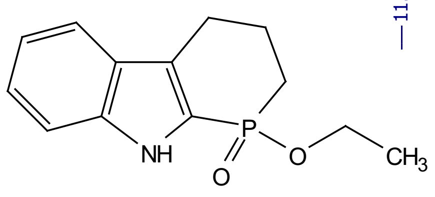
NH
P
O
O
CH3

12

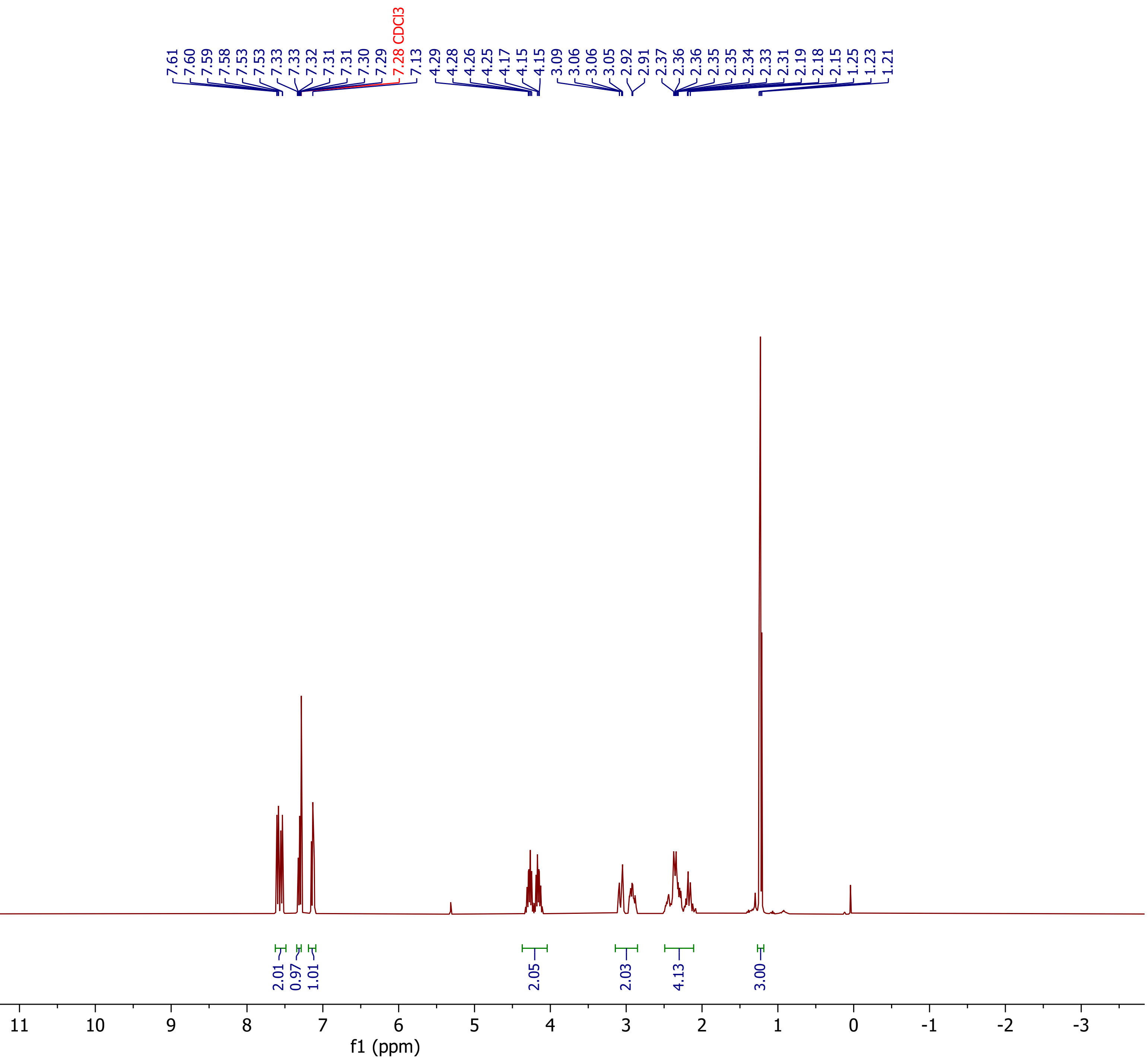
11.74
7.61
7.60
7.59
7.58
7.53
7.53
7.33
7.33
7.32
7.31
7.31
7.30
7.29
7.28 CDCl3
7.13
4.29
4.28
4.26
4.25
4.17
4.15
4.15
3.09
3.06
3.06
3.05
2.92
2.91
2.37
2.36
2.36
2.35
2.35
2.34
2.33
2.31
2.19
2.18
2.15
1.25
1.23
1.21
0.96
2.01
0.97
1.01
2.05
2.03
4.13
3.00
f1 (ppm)

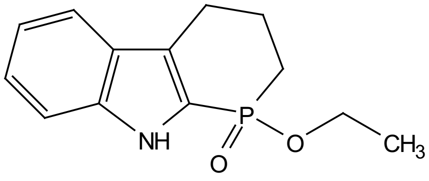

NH
P
O
O
CH3

12

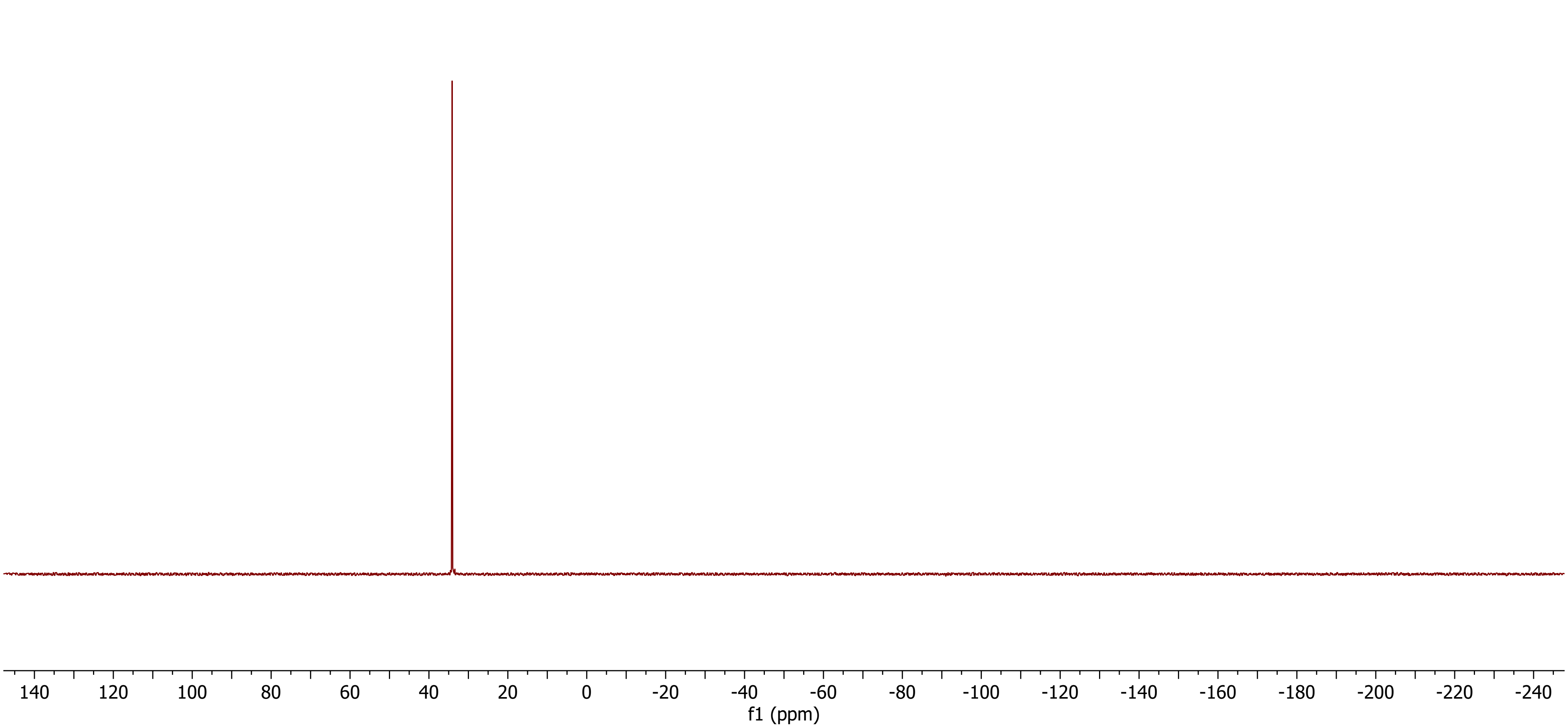

34.12
140
120
100
80
60
40
20
0
-20
-40
-60
-80
-100
-120
-140
-160
-180
-200
-220
-240
f1 (ppm)

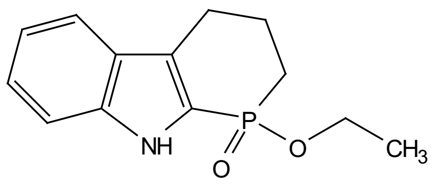

12

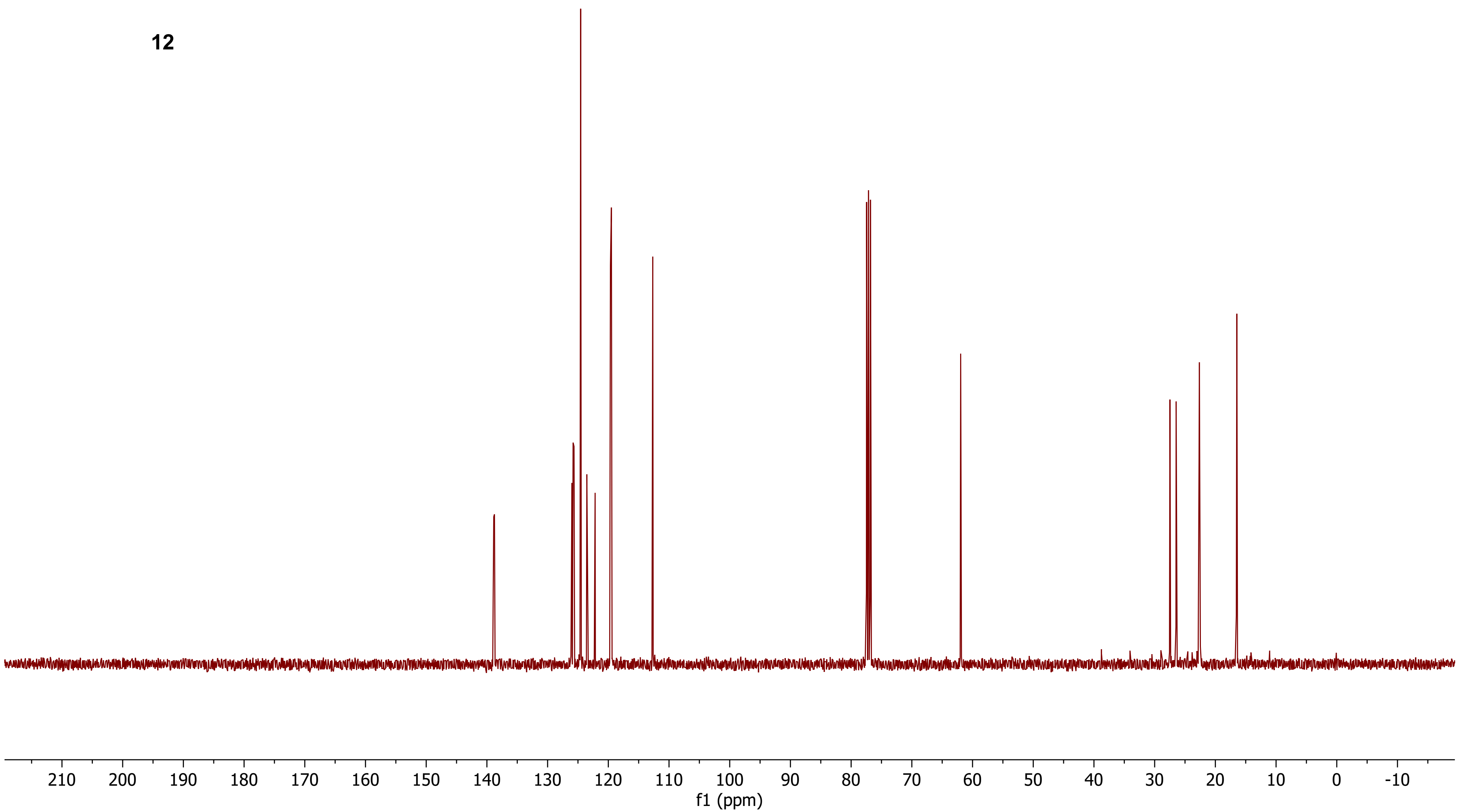

138.87
138.75
126.11
125.99
125.80
125.65
124.55
123.54
122.17
119.66
119.65
119.49
112.69
112.67
77.45 CDCl3
77.34 CDCl3
77.13 CDCl3
76.82 CDCl3
61.95
61.89
27.48
26.46
22.68
22.64
22.59
22.53
16.53
16.46
210 200 190 180 170 160 150 140 130 120 110 100 90 80 70 60 50 40 30 20 10 0 -10
f1 (ppm)

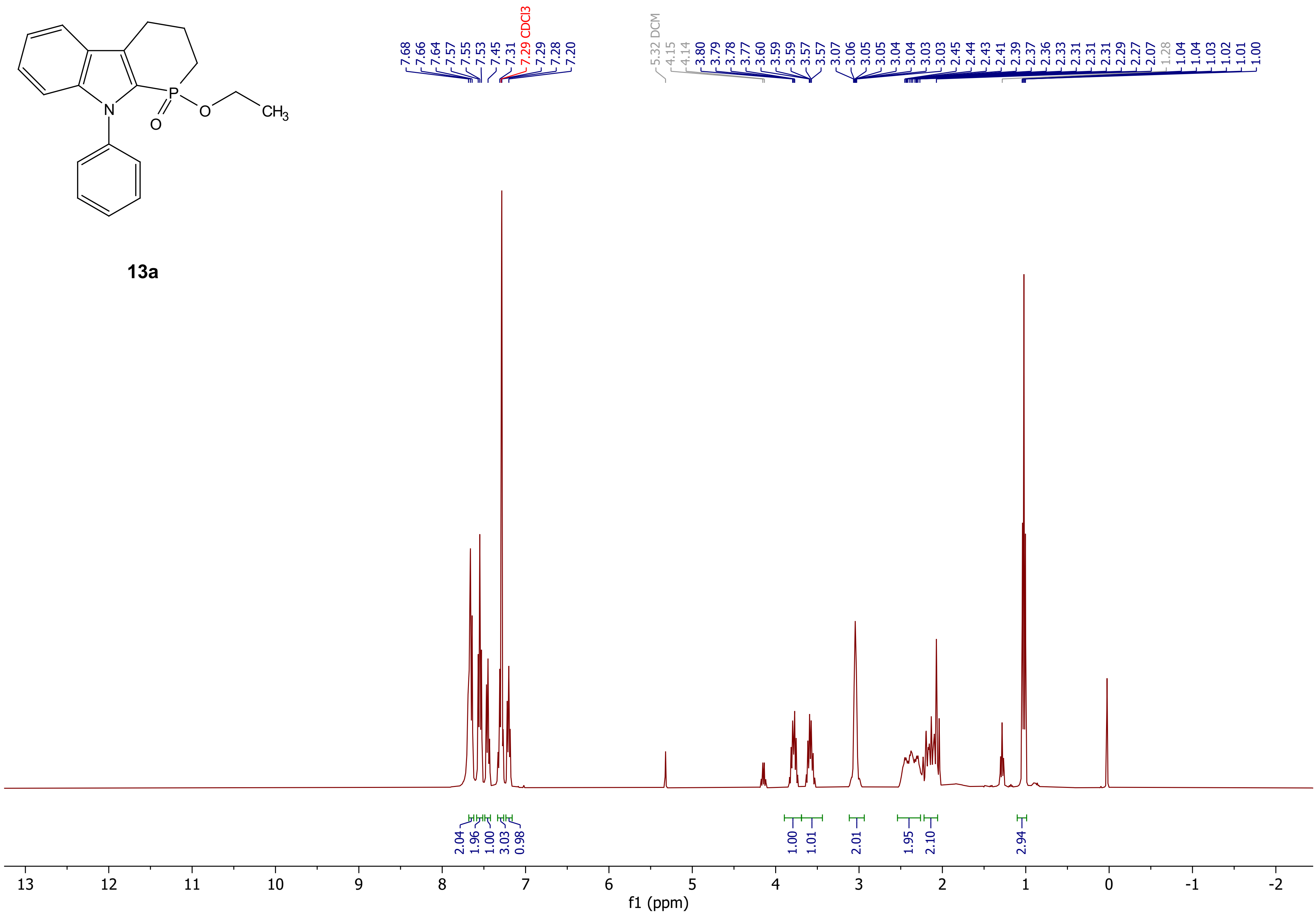

CH3
N
P
O
O
7.68
7.66
7.64
7.57
7.55
7.53
7.45
7.31
7.29 CDCl3
7.29
7.28
7.20
5.32 DCM
4.15
4.14
3.80
3.79
3.78
3.77
3.60
3.59
3.59
3.57
3.57
3.07
3.06
3.05
3.05
3.04
3.04
3.03
3.03
2.45
2.44
2.43
2.41
2.39
2.37
2.36
2.33
2.31
2.31
2.31
2.29
2.27
2.07
1.28
1.04
1.04
1.03
1.02
1.01
1.00
2.04
1.96
1.00
3.03
0.98
1.00
1.01
2.01
1.95
2.10
2.94
13
12
11
10
9
8
7
6
5
4
3
2
1
0
-1
-2
f1 (ppm)

13a

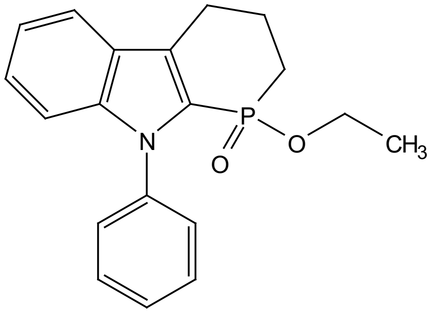

13a

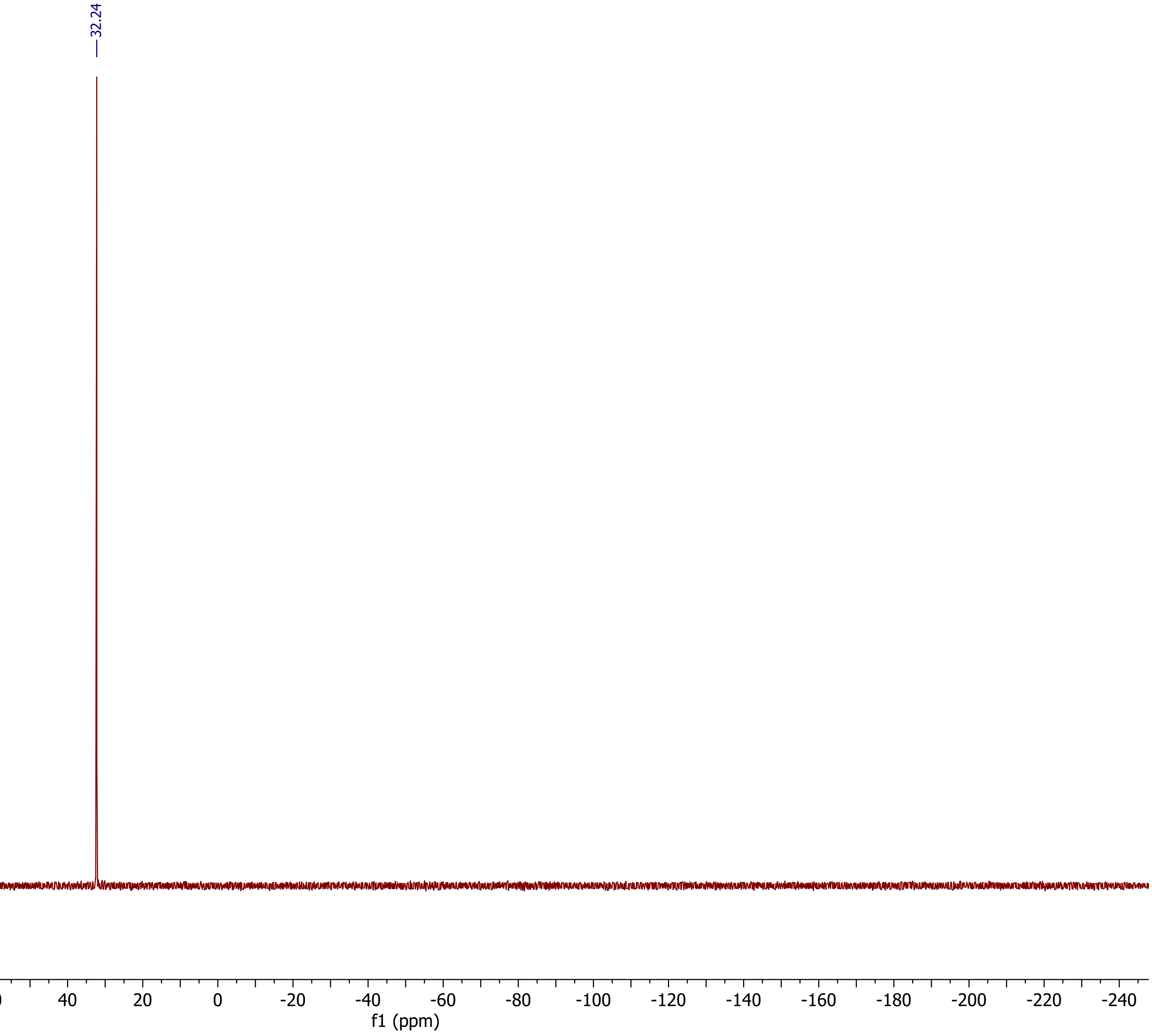

32.24
140
120
100
80
60
40
20
0
-20
-40
-60
-80
-100
-120
-140
-160
-180
-200
-220
-240
f1 (ppm)

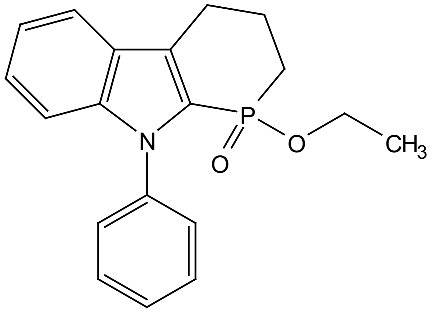
N
P
O
O
CH3

13a

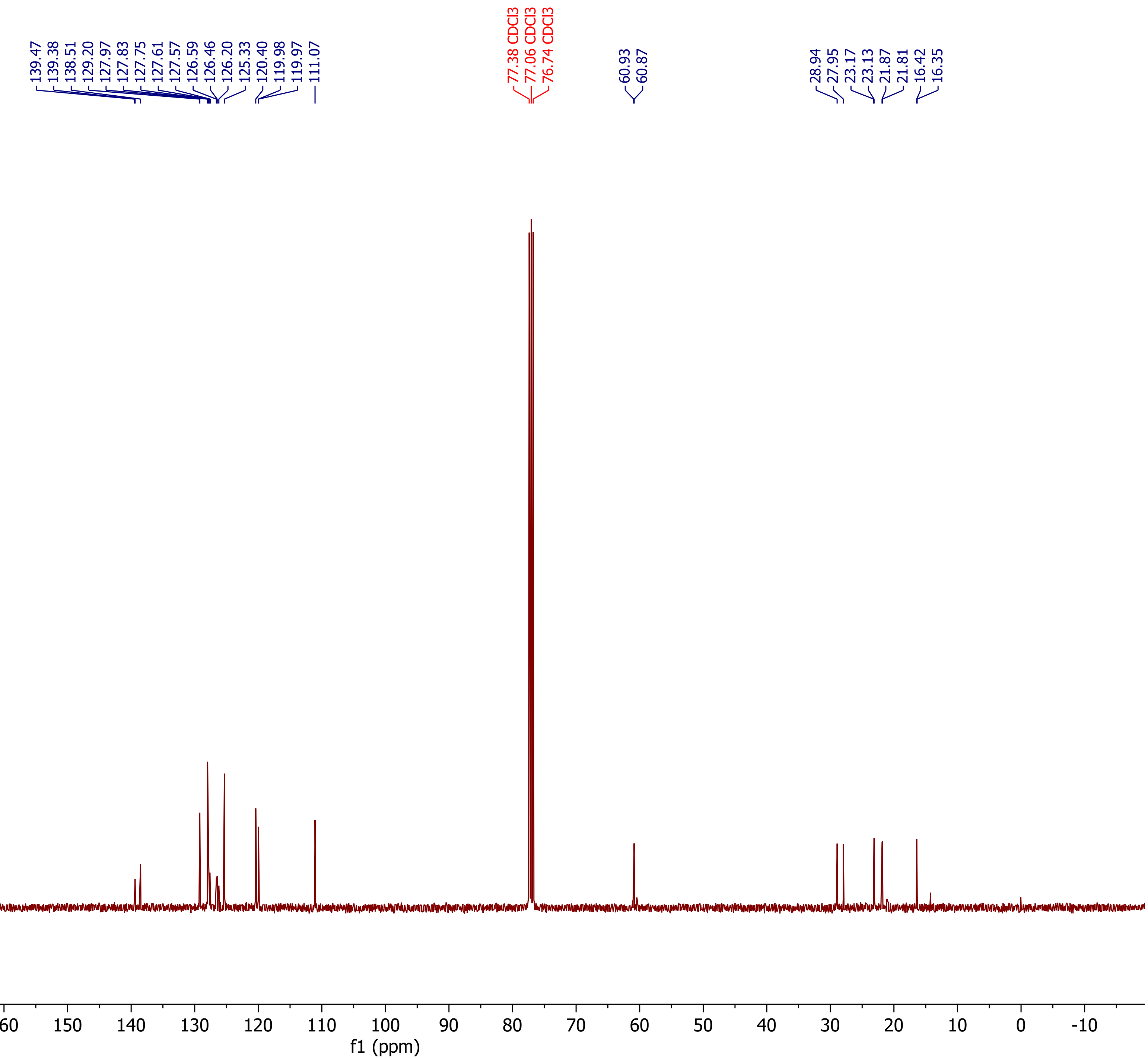
139.47
139.38
138.51
129.20
127.97
127.83
127.75
127.61
127.57
126.59
126.46
126.20
125.33
120.40
119.98
119.97
111.07
77.38 CDCl3
77.06 CDCl3
76.74 CDCl3
60.93
60.87
28.94
27.95
23.17
23.13
21.87
21.81
16.42
16.35
210 200 190 180 170 160 150 140 130 120 110 100 90 80 70 60 50 40 30 20 10 0 -10
f1 (ppm)

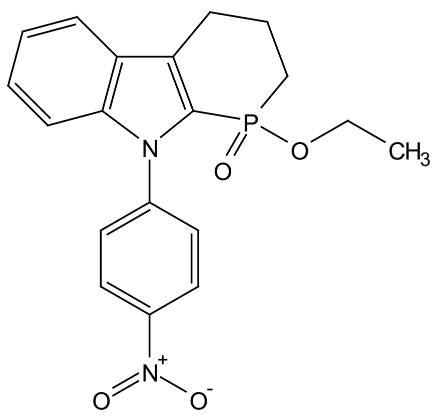

13b

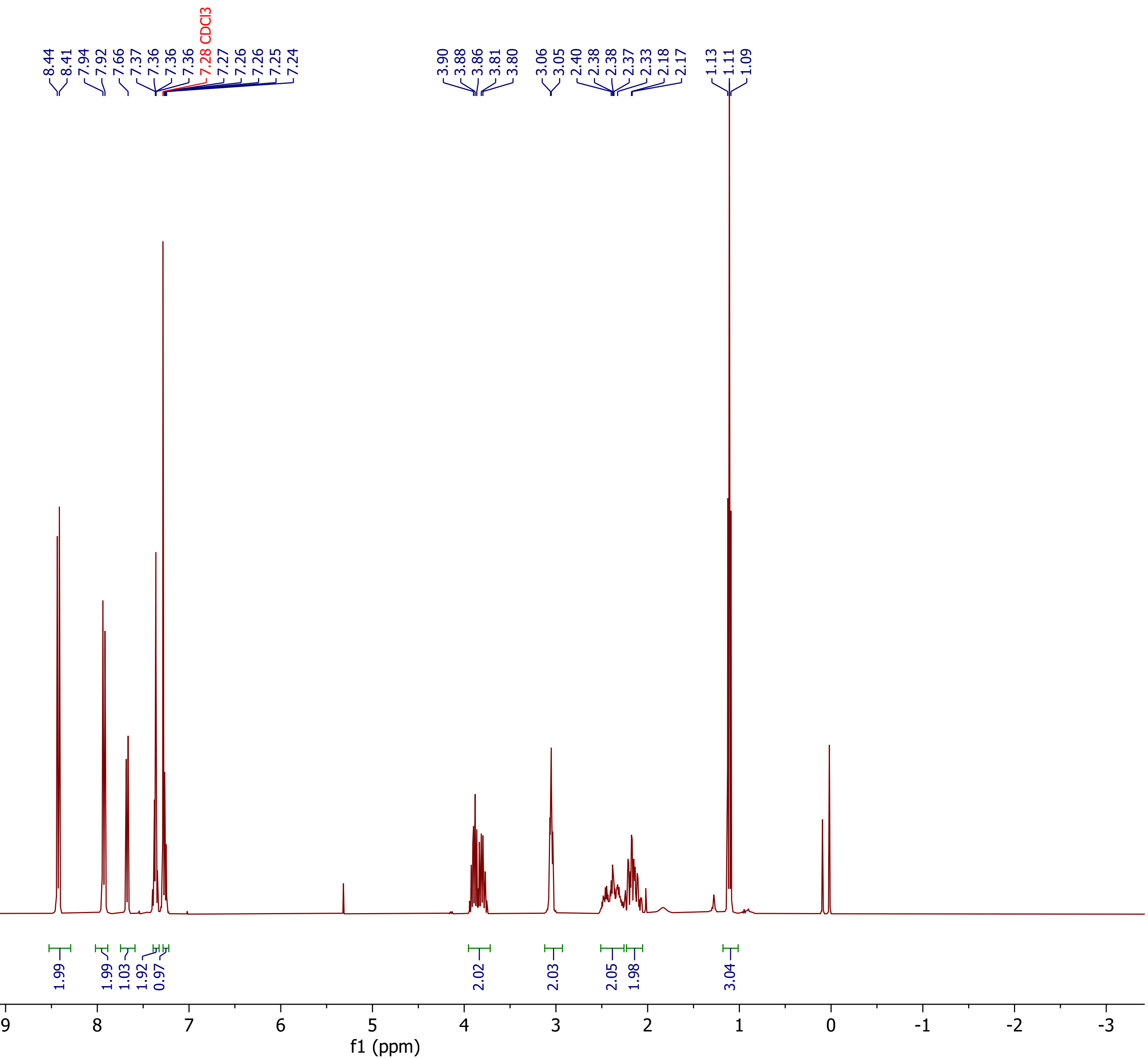

8.44
8.41
7.94
7.92
7.66
7.37
7.36
7.36
7.36
7.28 CDCl3
7.27
7.26
7.26
7.25
7.24
3.90
3.88
3.86
3.81
3.80
3.06
3.05
2.40
2.38
2.38
2.37
2.33
2.18
2.17
1.13
1.11
1.09
1.99
1.99
1.03
1.92
0.97
2.02
2.03
2.05
1.98
3.04
f1 (ppm)

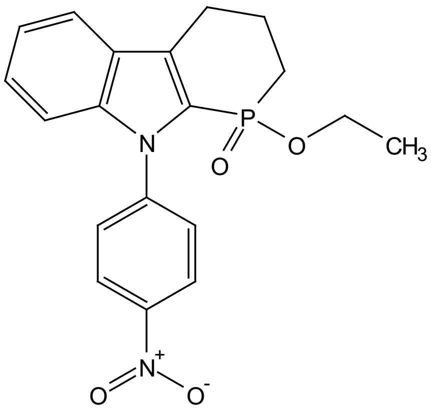
N
P
O
O
CH3
N+
O
O-

13b

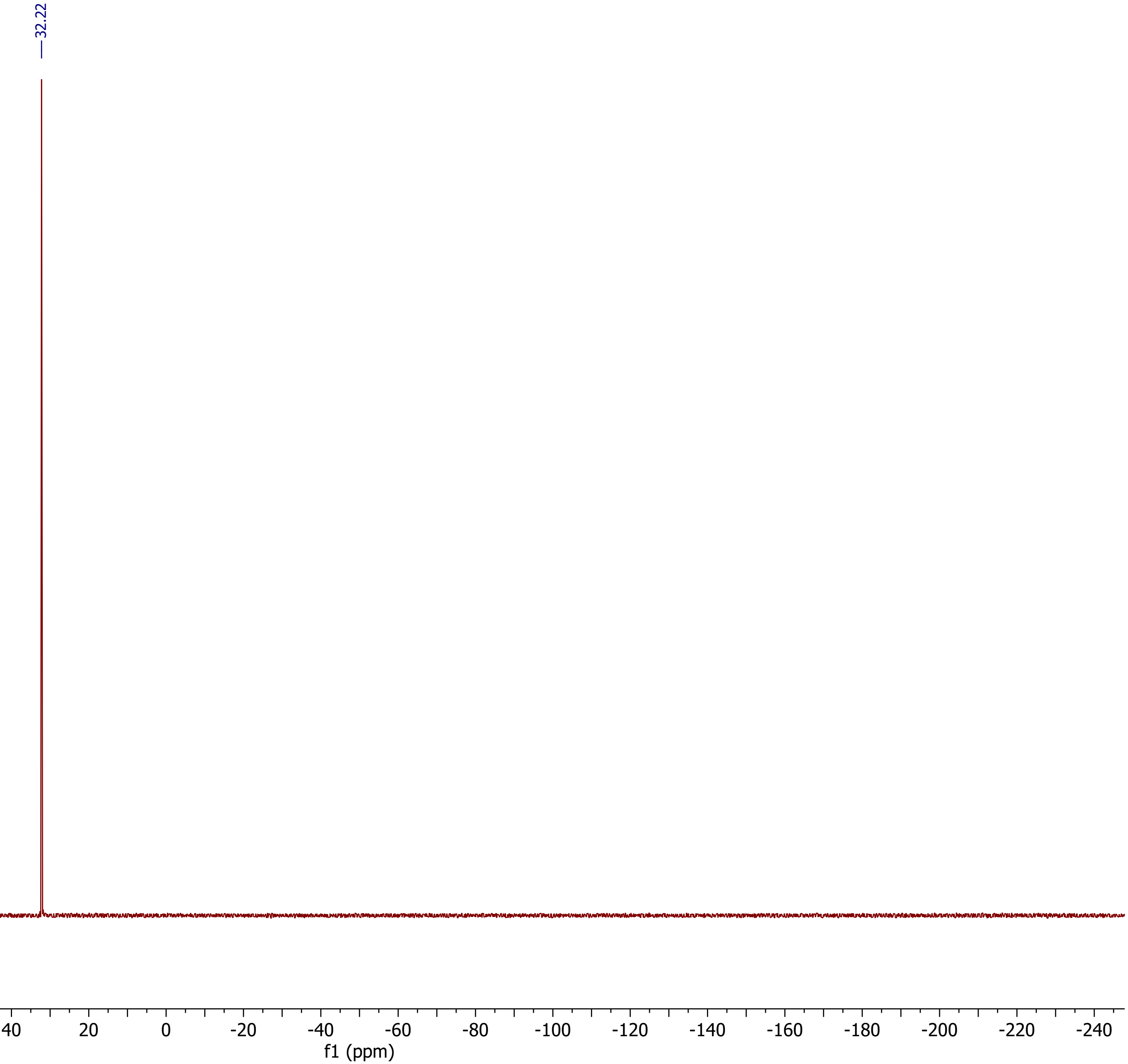
32.22
140
120
100
80
60
40
20
0
-20
-40
-60
-80
-100
-120
-140
-160
-180
-200
-220
-240
f1 (ppm)

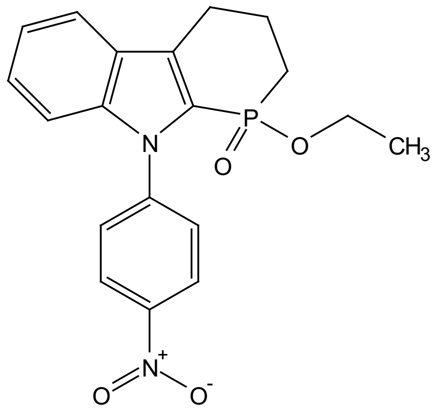
N
P
O
O
CH3
N+
O
O-

13b

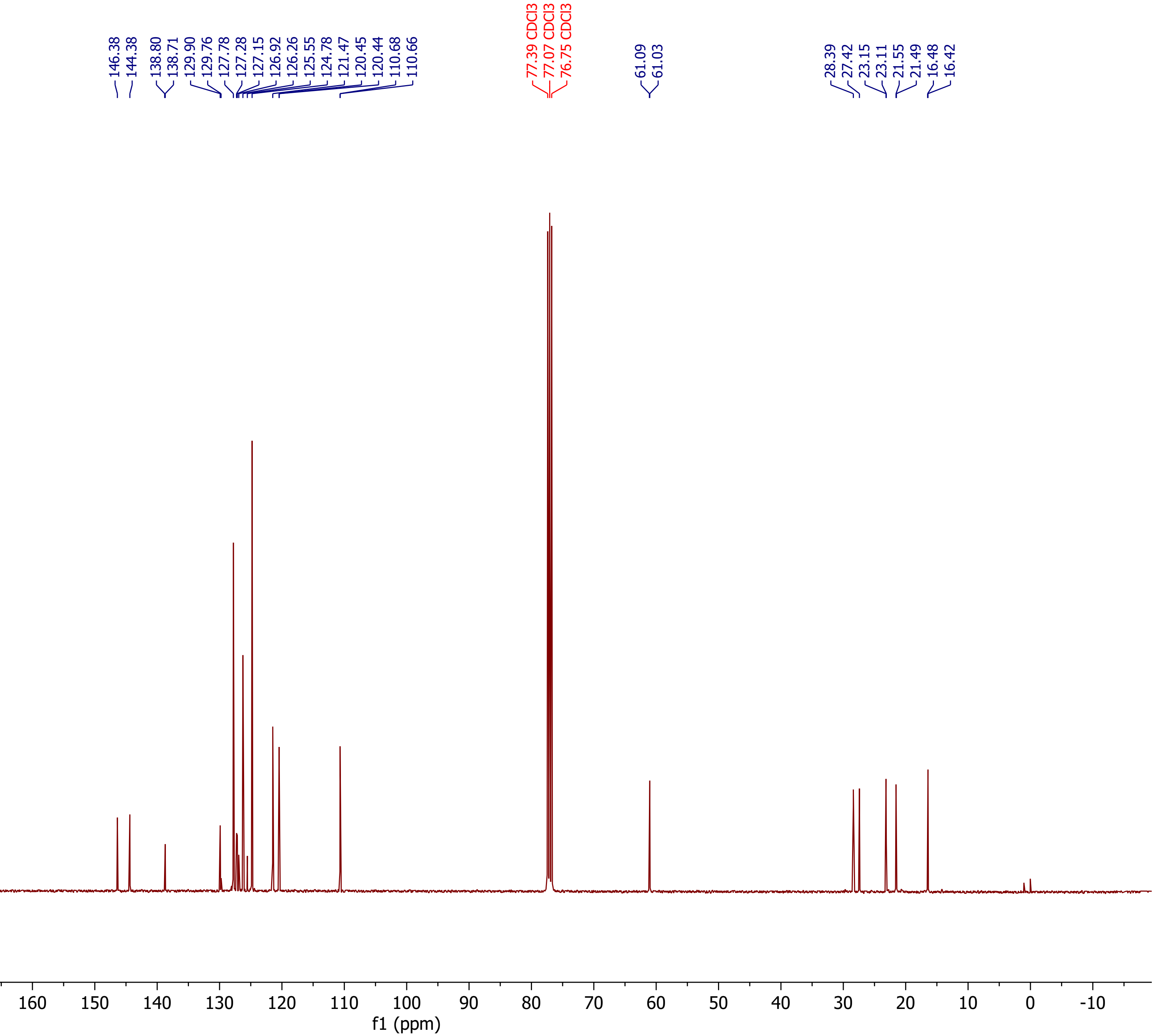
146.38
144.38
138.80
138.71
129.90
129.76
127.78
127.28
127.15
126.92
126.26
125.55
124.78
121.47
120.45
120.44
110.68
110.66
77.39 CDCl3
77.07 CDCl3
76.75 CDCl3
61.09
61.03
28.39
27.42
23.15
23.11
21.55
21.49
16.48
16.42
210
200
190
180
170
160
150
140
130
120
110
100
90
80
70
60
50
40
30
20
10
0
-10
f1 (ppm)

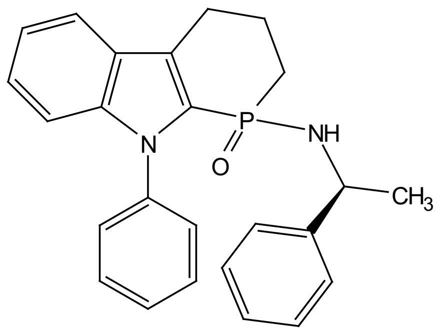

N
P
O
NH
CH3

14a

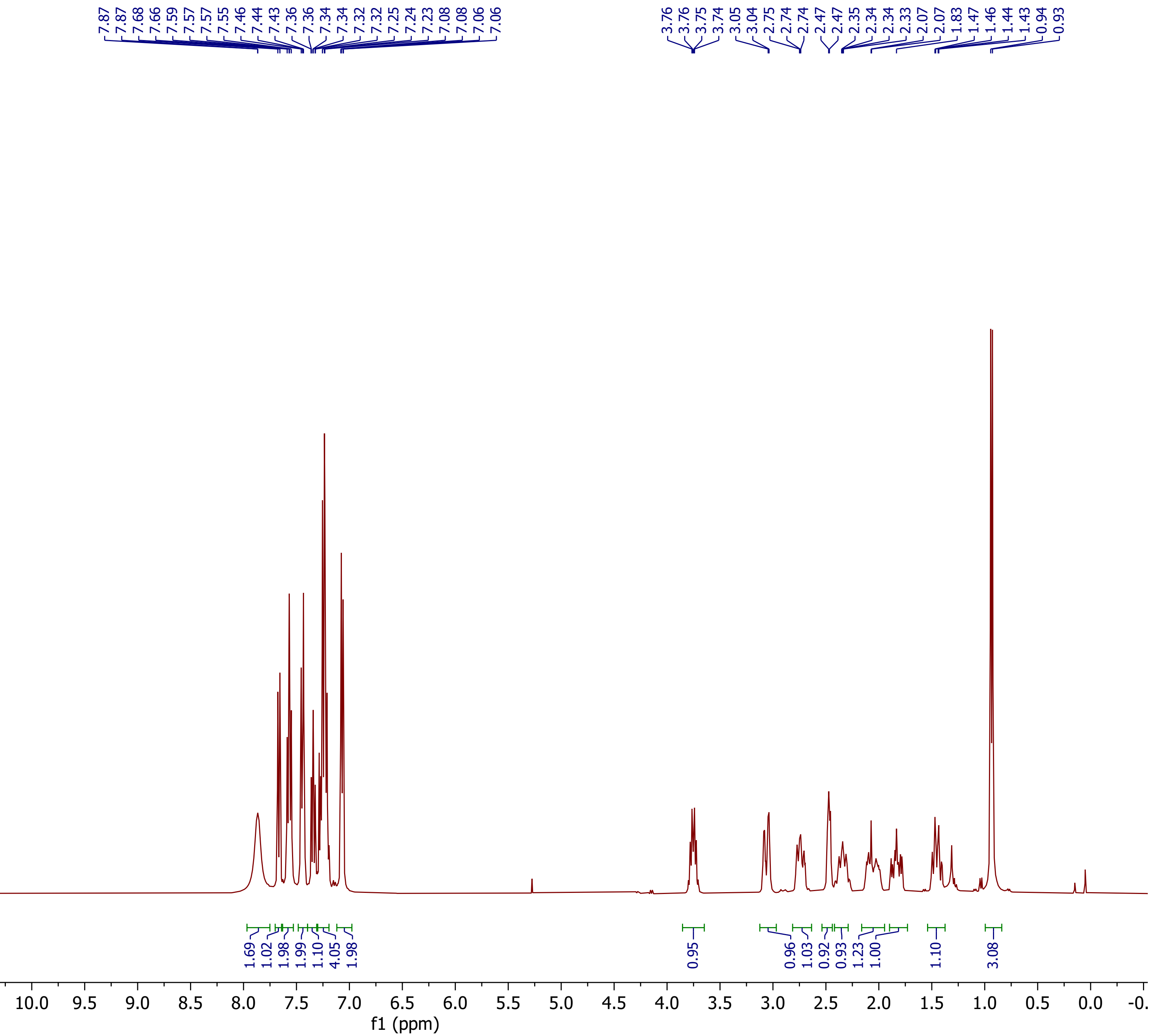

7.87
7.87
7.68
7.66
7.59
7.57
7.57
7.55
7.46
7.44
7.43
7.36
7.36
7.34
7.34
7.32
7.32
7.25
7.24
7.23
7.08
7.08
7.06
7.06
3.76
3.76
3.75
3.74
3.05
3.04
2.75
2.74
2.74
2.47
2.47
2.35
2.34
2.34
2.33
2.07
2.07
1.83
1.47
1.46
1.44
1.43
0.94
0.93
1.69
1.02
1.98
1.99
1.10
4.05
1.98
0.95
0.96
1.03
0.92
0.93
1.23
1.00
1.10
3.08
13.0
12.5
12.0
11.5
11.0
10.5
10.0
9.5
9.0
8.5
8.0
7.5
7.0
6.5
6.0
5.5
5.0
4.5
4.0
3.5
3.0
2.5
2.0
1.5
1.0
0.5
0.0
-0.
f1 (ppm)

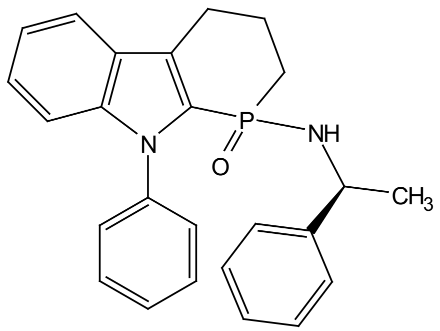

Racemic Mixture **14a**

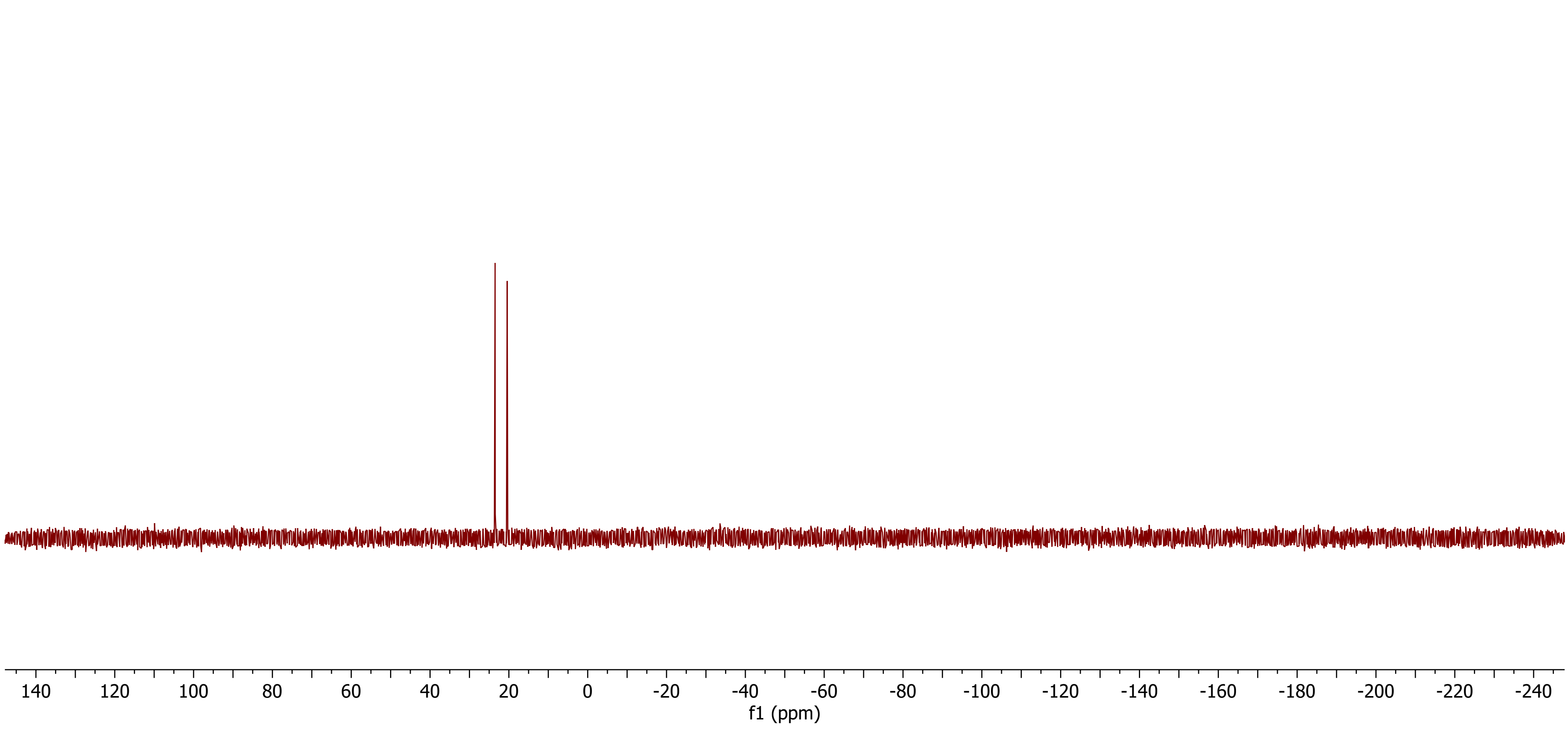

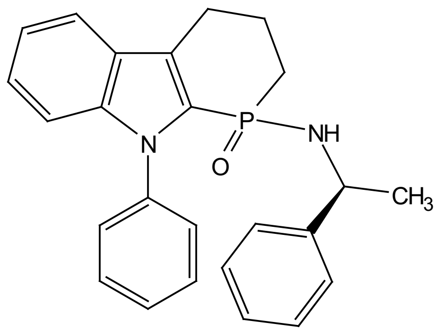
N
P
O
NH
CH3

14a

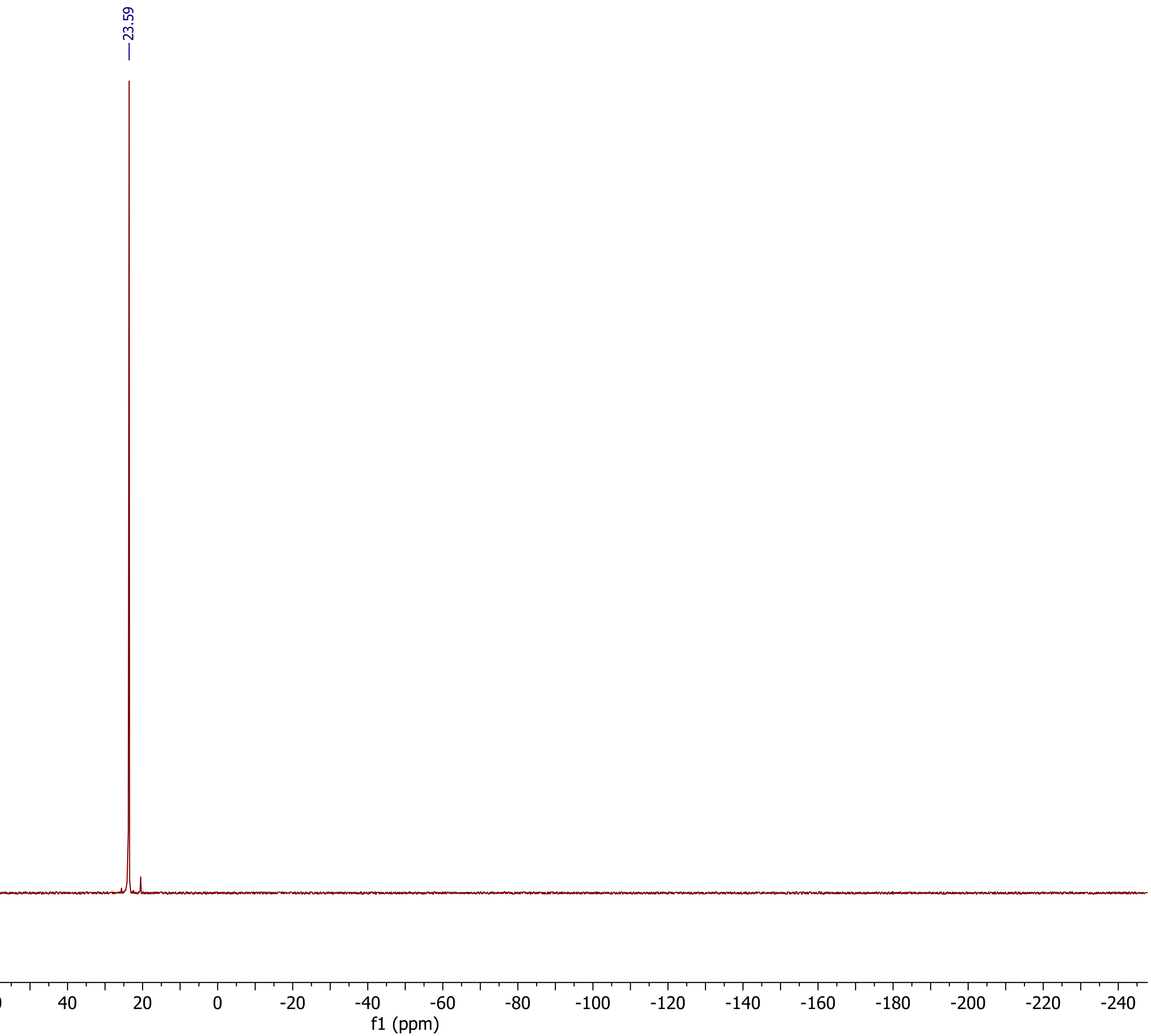
23.59
140 120 100 80 60 40 20 0 -20 -40 -60 -80 -100 -120 -140 -160 -180 -200 -220 -240
f1 (ppm)

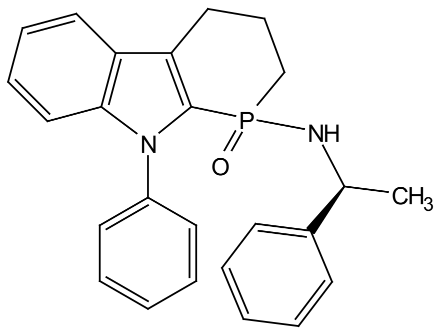
N
P
O
NH
CH3

14a

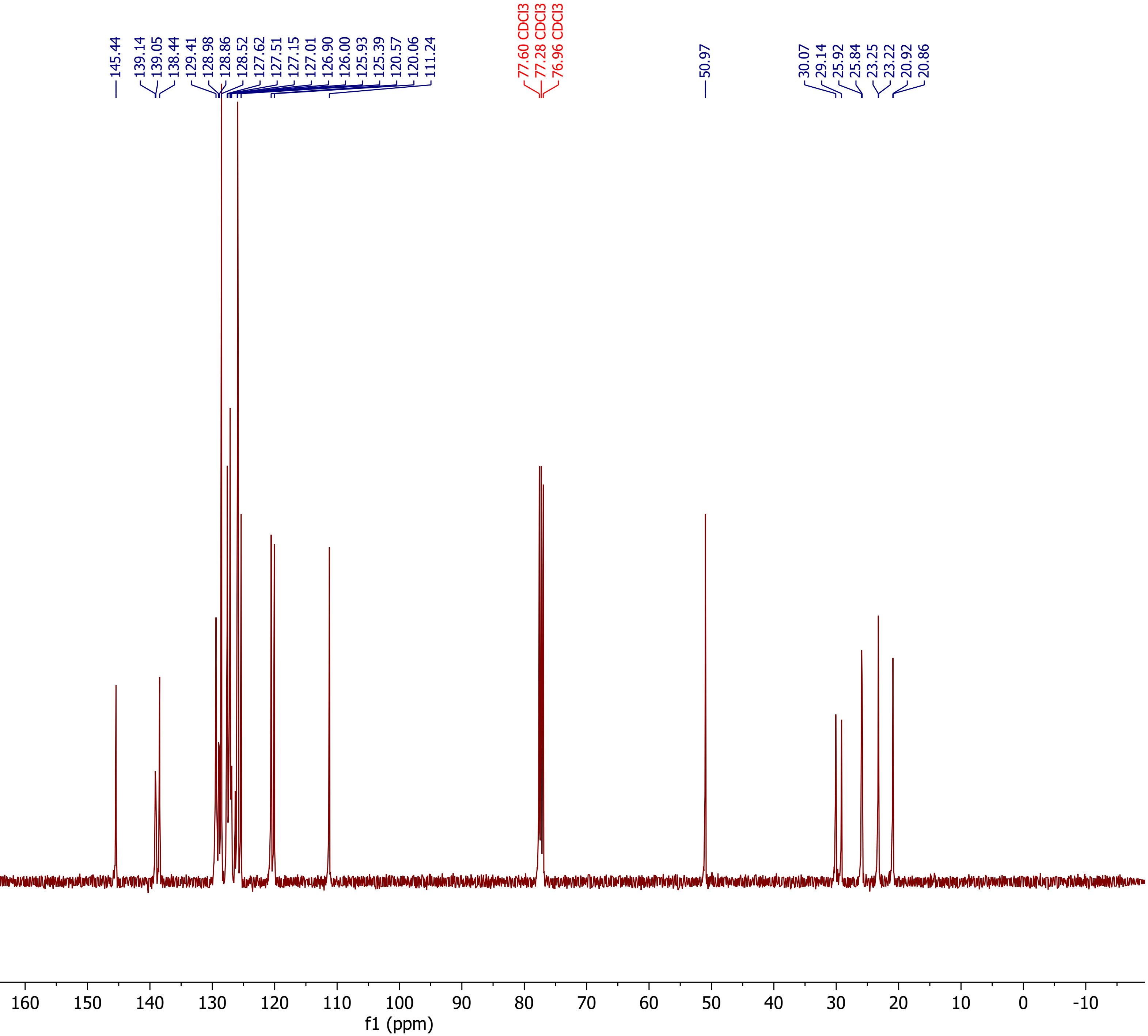
145.44
139.14
139.05
138.44
129.41
128.98
128.86
128.52
127.62
127.51
127.15
127.01
126.90
126.00
125.93
125.39
120.57
120.06
111.24
77.60 CDCl3
77.28 CDCl3
76.96 CDCl3
50.97
30.07
29.14
25.92
25.84
23.25
23.22
20.92
20.86
210
200
190
180
170
160
150
140
130
120
110
100
90
80
70
60
50
40
30
20
10
0
-10
f1 (ppm)

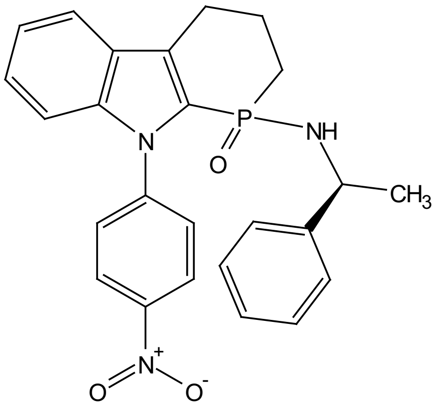
N
P
O
NH
CH3
N+
O
O-

14b

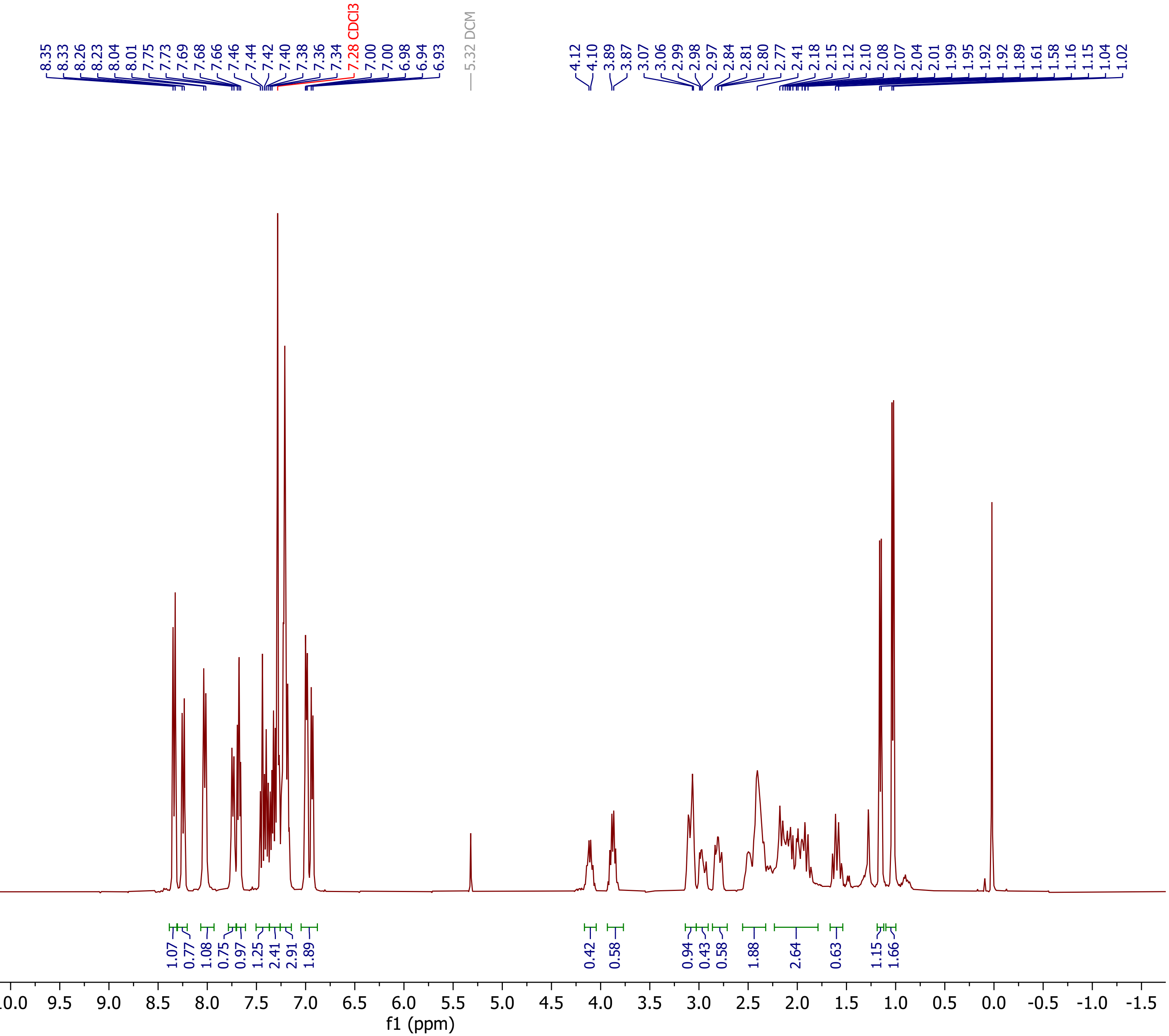
8.35
8.33
8.26
8.23
8.04
8.01
7.75
7.73
7.69
7.68
7.66
7.46
7.44
7.42
7.40
7.38
7.36
7.34
7.28 CDCl3
7.00
7.00
6.98
6.94
6.93
5.32 DCM
4.12
4.10
3.89
3.87
3.07
3.06
2.99
2.98
2.97
2.84
2.81
2.80
2.77
2.41
2.18
2.15
2.12
2.10
2.08
2.07
2.04
2.01
1.99
1.95
1.92
1.92
1.89
1.61
1.58
1.16
1.15
1.04
1.02
1.07
0.77
1.08
0.75
0.97
1.25
2.41
2.91
1.89
0.42
0.58
0.94
0.43
0.58
1.88
2.64
0.63
1.15
1.66
13.0 12.5 12.0 11.5 11.0 10.5 10.0 9.5 9.0 8.5 8.0 7.5 7.0 6.5 6.0 5.5 5.0 4.5 4.0 3.5 3.0 2.5 2.0 1.5 1.0 0.5 0.0 -0.5 -1.0 -1.5
f1 (ppm)

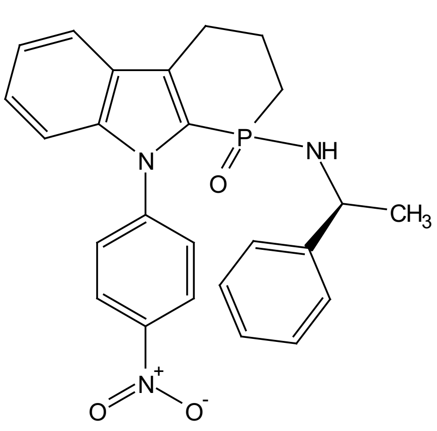
N
P
O
NH
CH3
N+
O
O-

14b

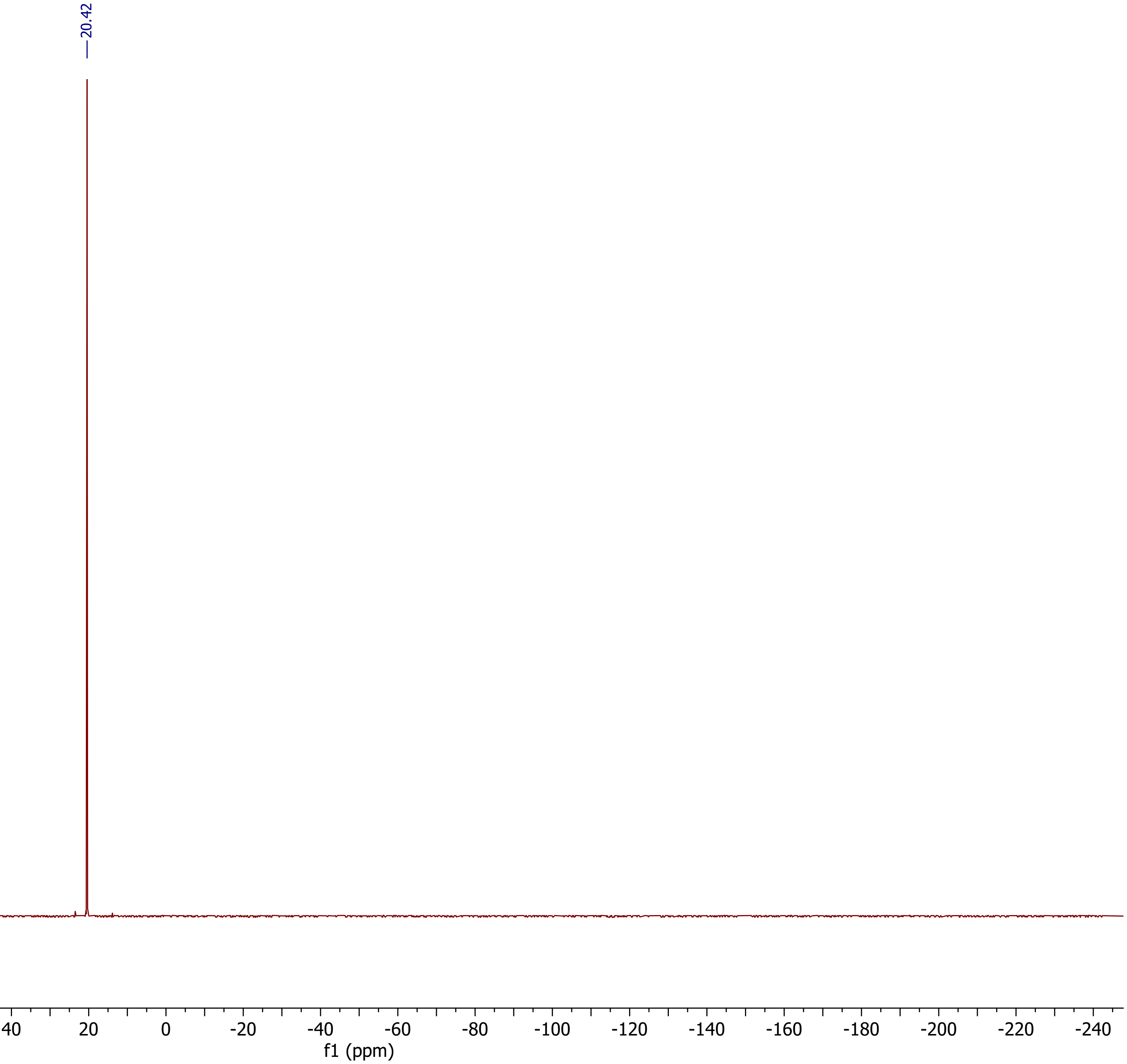
20.42
140
120
100
80
60
40
20
0
-20
-40
-60
-80
-100
-120
-140
-160
-180
-200
-220
-240
f1 (ppm)

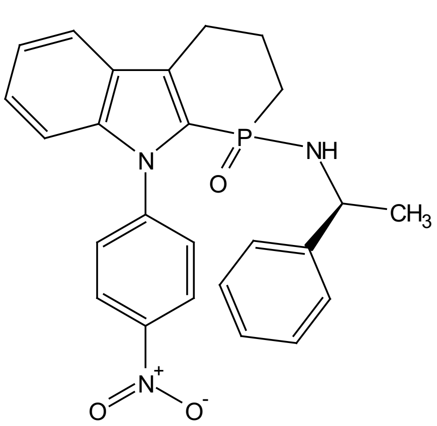

NH
CH3
N
P
O
O
N+
O-

14b

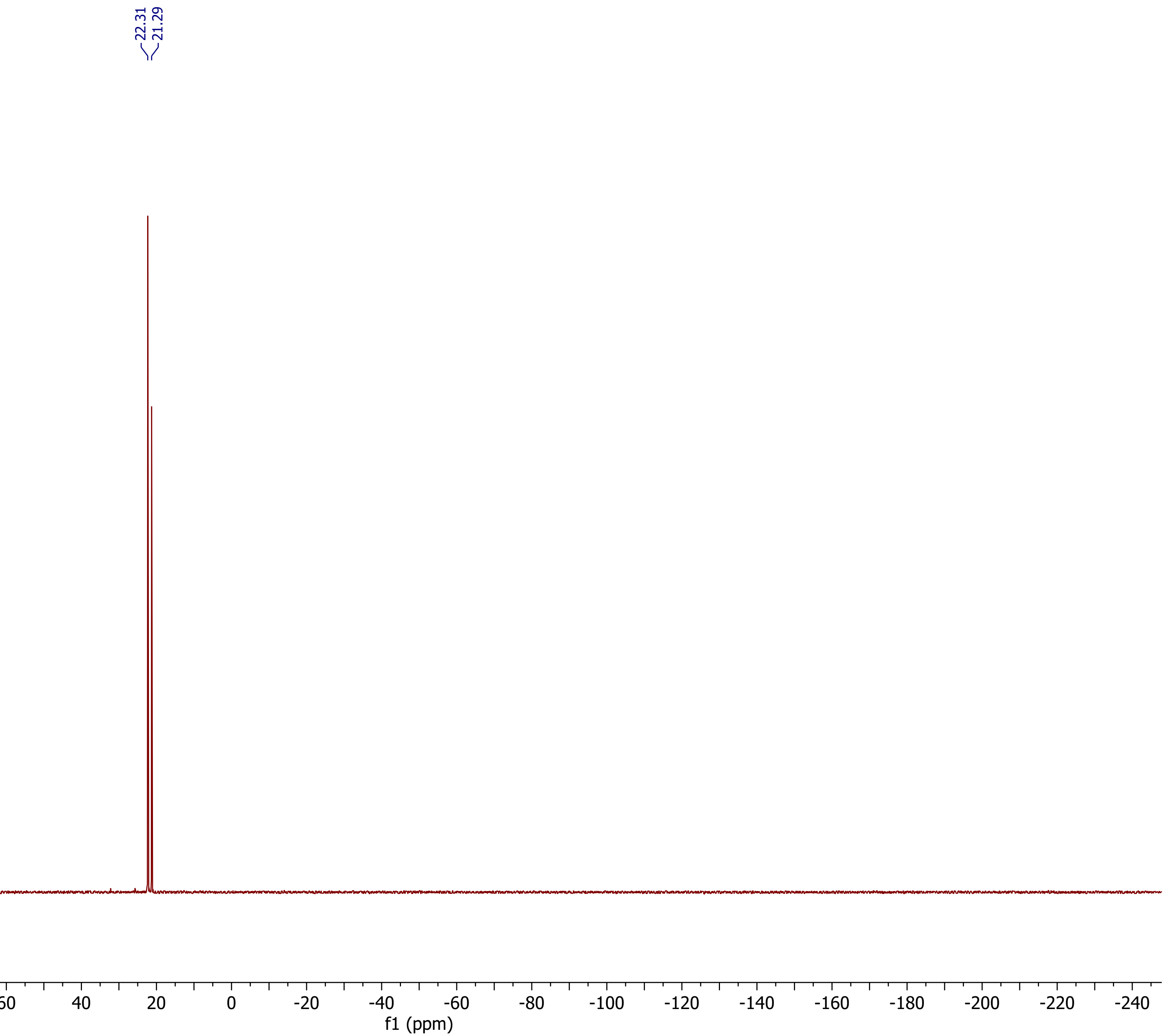

22.31
21.29
140
120
100
80
60
40
20
0
-20
-40
-60
-80
-100
-120
-140
-160
-180
-200
-220
-240
f1 (ppm)

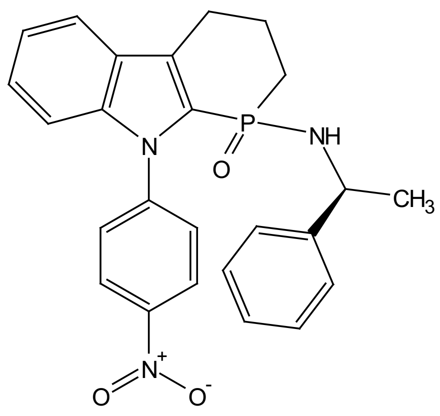

14b

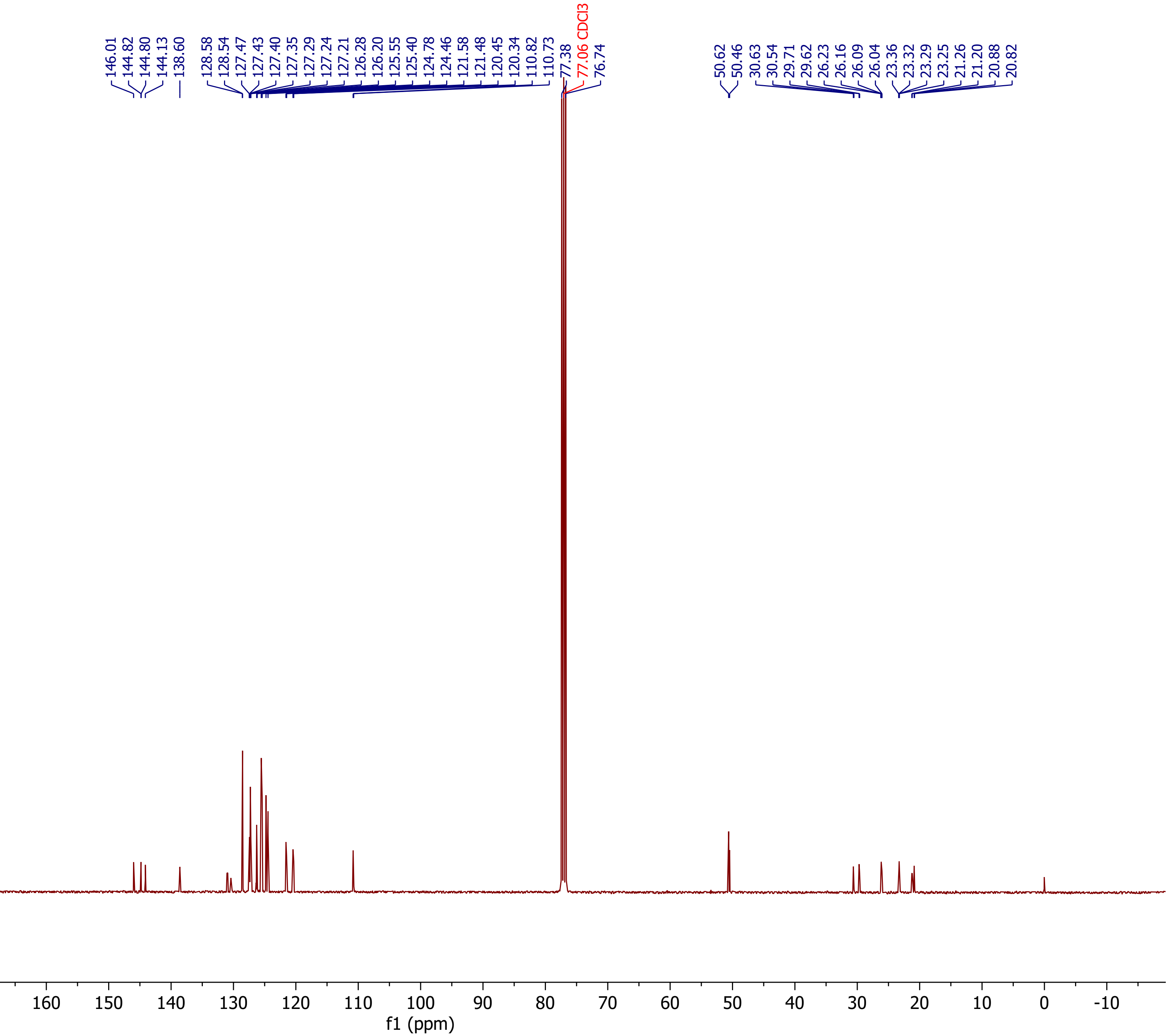

146.01
144.82
144.80
144.13
138.60
128.58
128.54
127.47
127.43
127.40
127.35
127.29
127.24
127.21
126.28
126.20
125.55
125.40
124.78
124.46
121.58
121.48
120.45
120.34
110.82
110.73
77.38
77.06 CDCl3
76.74
50.62
50.46
30.63
30.54
29.71
29.62
26.23
26.16
26.09
26.04
23.36
23.32
23.29
23.25
21.26
21.20
20.88
20.82
210 200 190 180 170 160 150 140 130 120 110 100 90 80 70 60 50 40 30 20 10 0 -10
f1 (ppm)

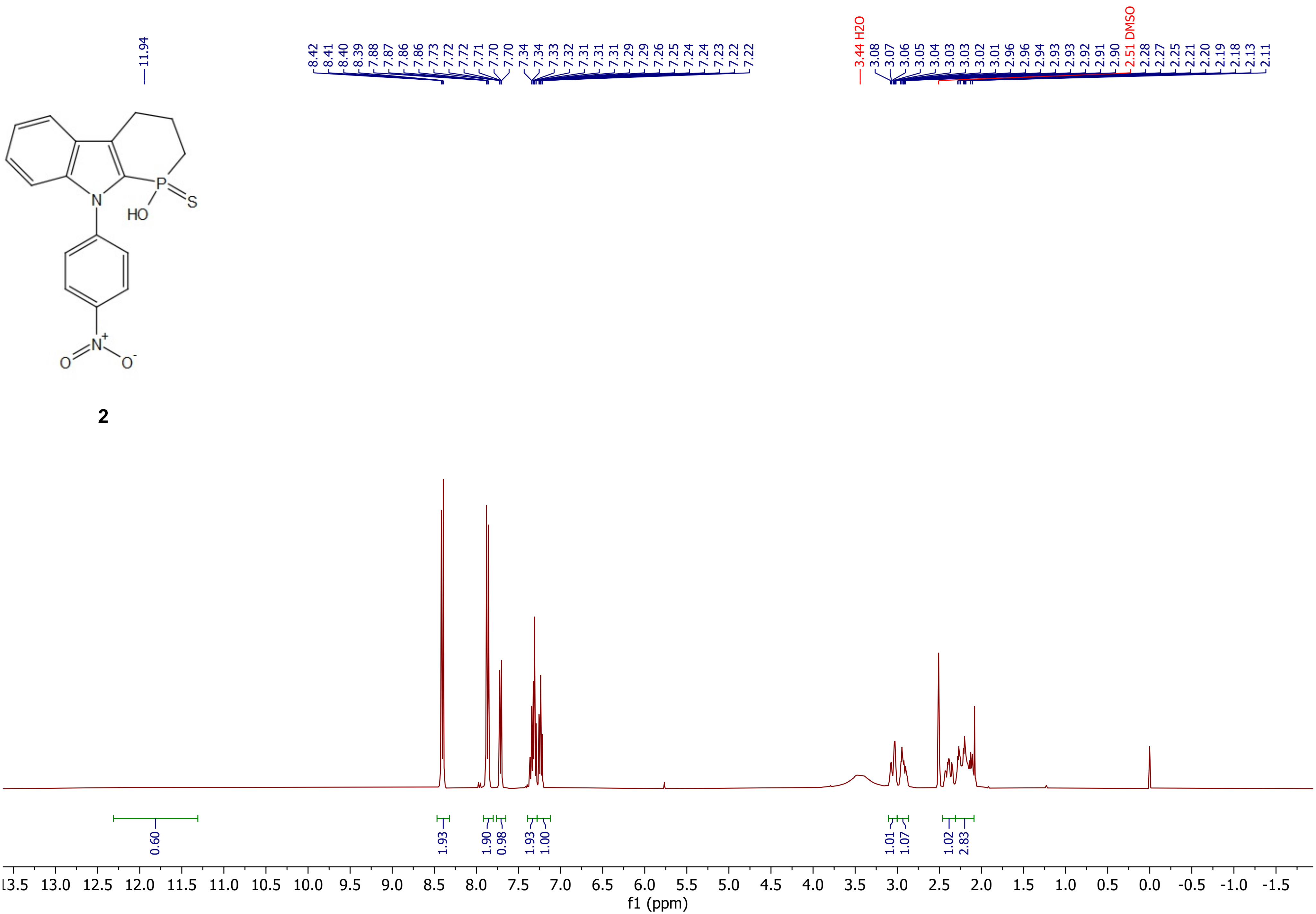
11.94
8.42 8.41 8.40 8.39 7.88 7.87 7.86 7.86 7.73 7.72 7.72 7.71 7.70 7.70 7.34 7.34 7.33 7.32 7.31 7.31 7.31 7.29 7.29 7.26 7.25 7.24 7.24 7.23 7.22 7.22
3.44 H2O
3.08 3.07 3.06 3.05 3.04 3.03 3.03 3.02 3.01 2.96 2.96 2.94 2.93 2.93 2.92 2.91 2.90
2.51 DMSO
2.28 2.27 2.25 2.21 2.20 2.19 2.18 2.13 2.11
N
P
S
HO
N+
O
O-
0.60
1.93
1.90
0.98
1.93
1.00
1.01
1.07
1.02
2.83
13.5 13.0 12.5 12.0 11.5 11.0 10.5 10.0 9.5 9.0 8.5 8.0 7.5 7.0 6.5 6.0 5.5 5.0 4.5 4.0 3.5 3.0 2.5 2.0 1.5 1.0 0.5 0.0 -0.5 -1.0 -1.5
f1 (ppm)

2

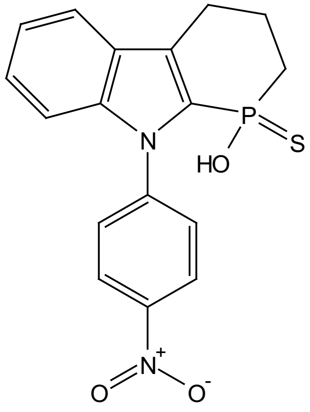
HO
P
S
N
O
N
O

2

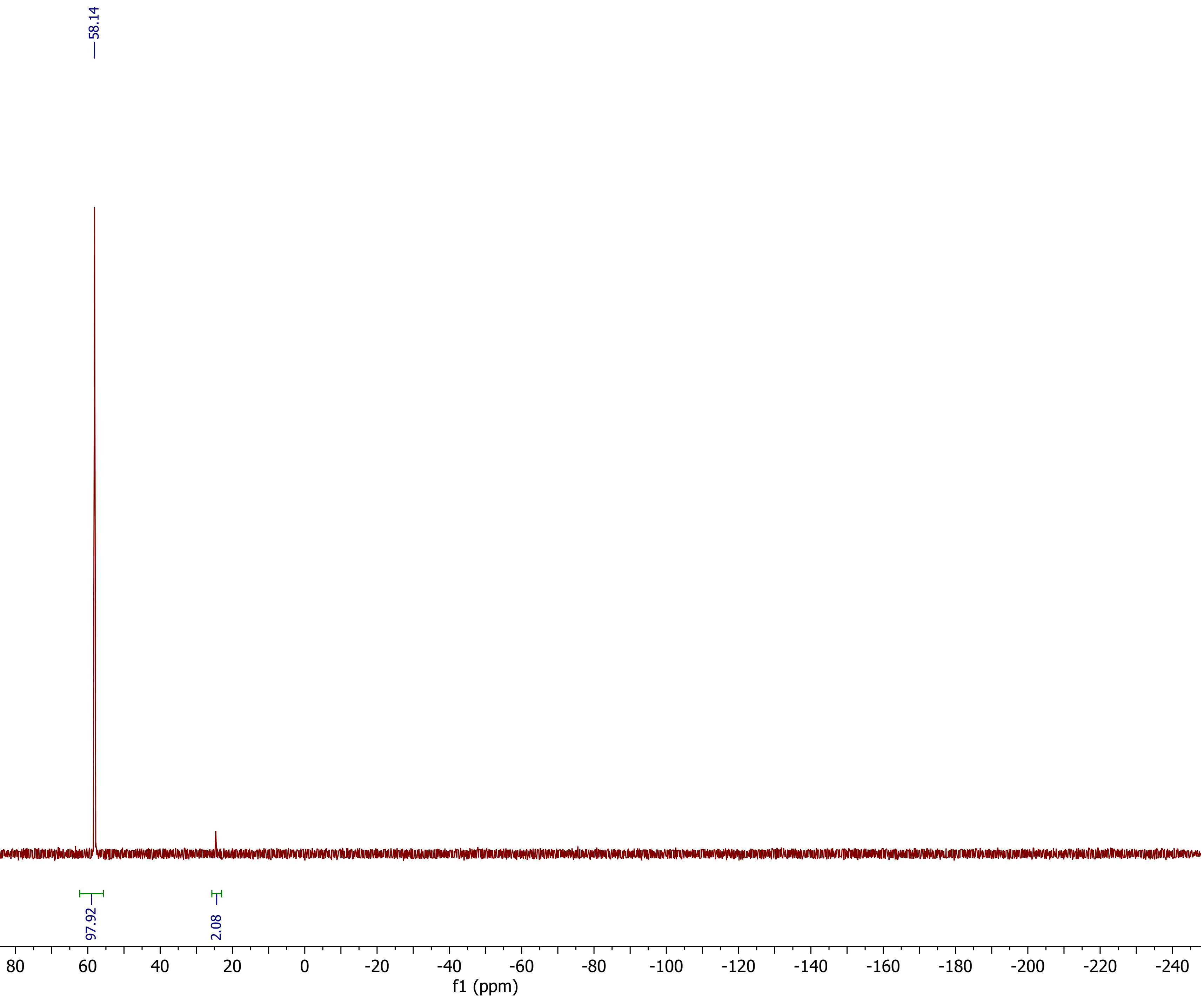
58.14
97.92
2.08
140
120
100
80
60
40
20
0
-20
-40
-60
-80
-100
-120
-140
-160
-180
-200
-220
-240
f1 (ppm)

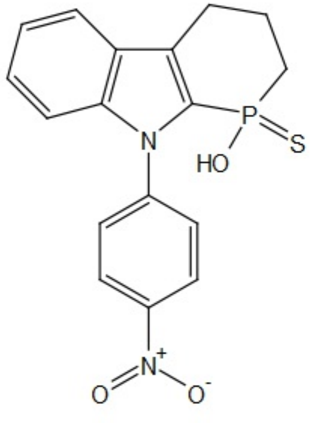
N
P
S
HO
N+
O
O-

2

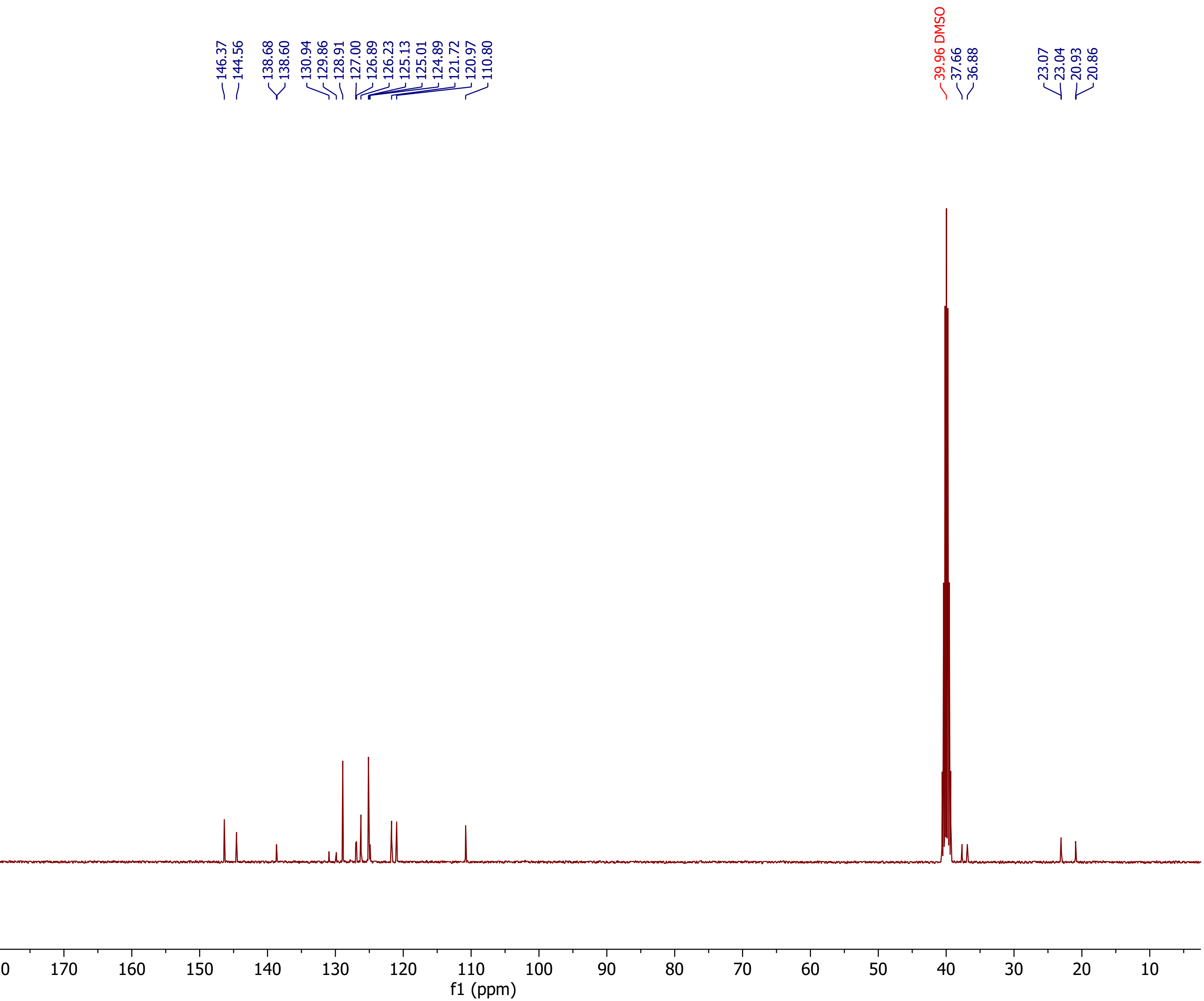
146.37
144.56
138.68
138.60
130.94
129.86
128.91
127.00
126.89
126.23
125.13
125.01
124.89
121.72
120.97
110.80
39.96 DMSO
37.66
36.88
23.07
23.04
20.93
20.86
210
200
190
180
170
160
150
140
130
120
110
100
90
80
70
60
50
40
30
20
10
f1 (ppm)

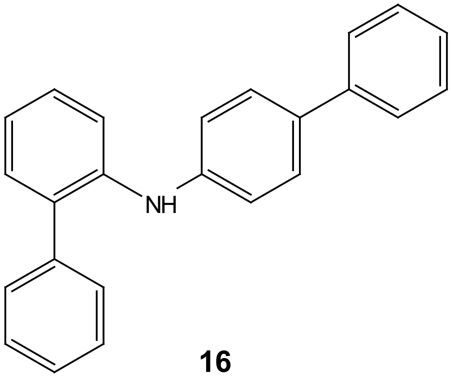
NH

16

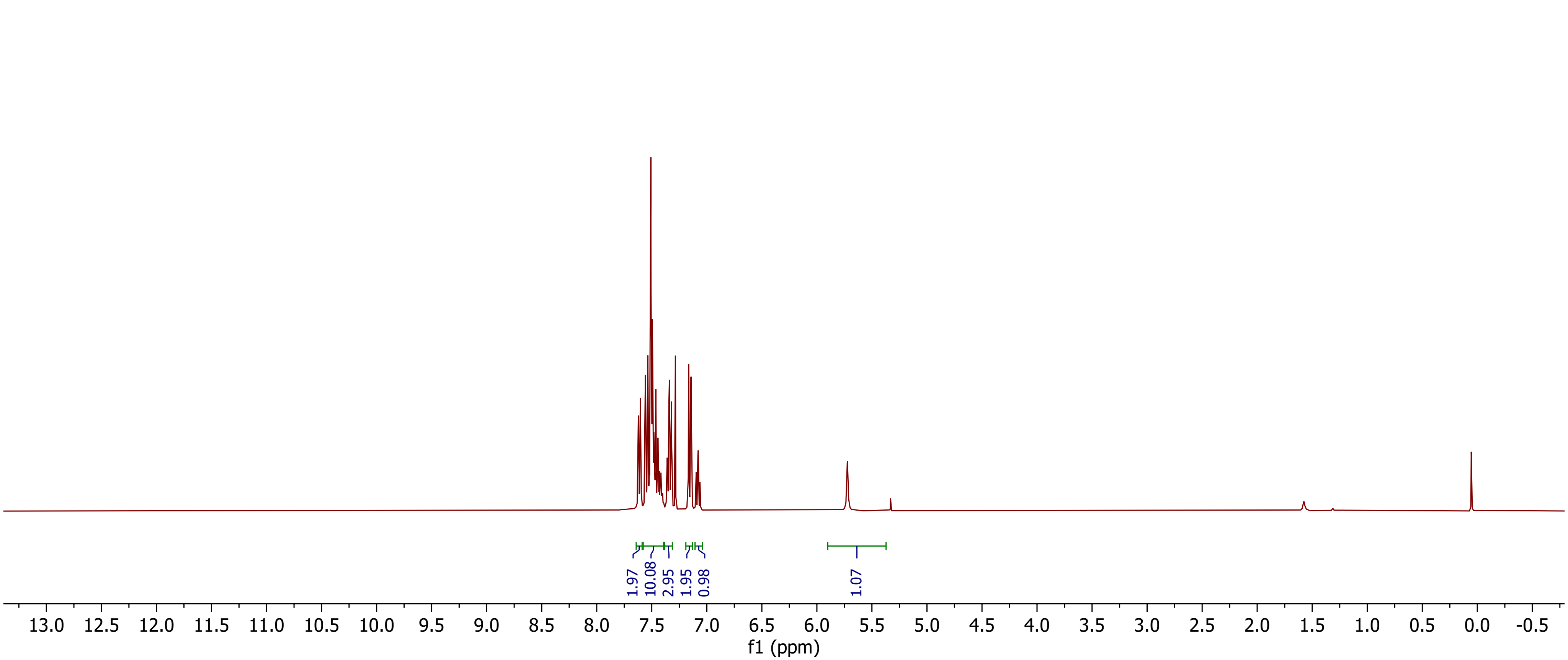
7.62
7.62
7.61
7.60
7.60
7.56
7.55
7.54
7.54
7.52
7.51
7.51
7.50
7.49
7.49
7.49
7.48
7.46
7.46
7.44
7.37
7.36
7.36
7.36
7.35
7.34
7.34
7.29 CDCl3
7.17
7.17
7.16
7.15
7.14
7.10
7.10
7.08
7.08
7.06
7.06
5.72
1.97
10.08
2.95
1.95
0.98
1.07
13.0 12.5 12.0 11.5 11.0 10.5 10.0 9.5 9.0 8.5 8.0 7.5 7.0 6.5 6.0 5.5 5.0 4.5 4.0 3.5 3.0 2.5 2.0 1.5 1.0 0.5 0.0 -0.5
f1 (ppm)

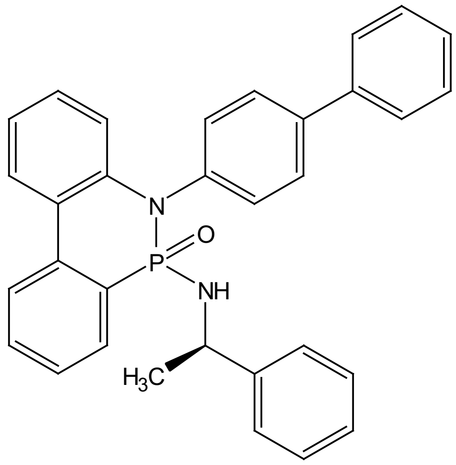

17

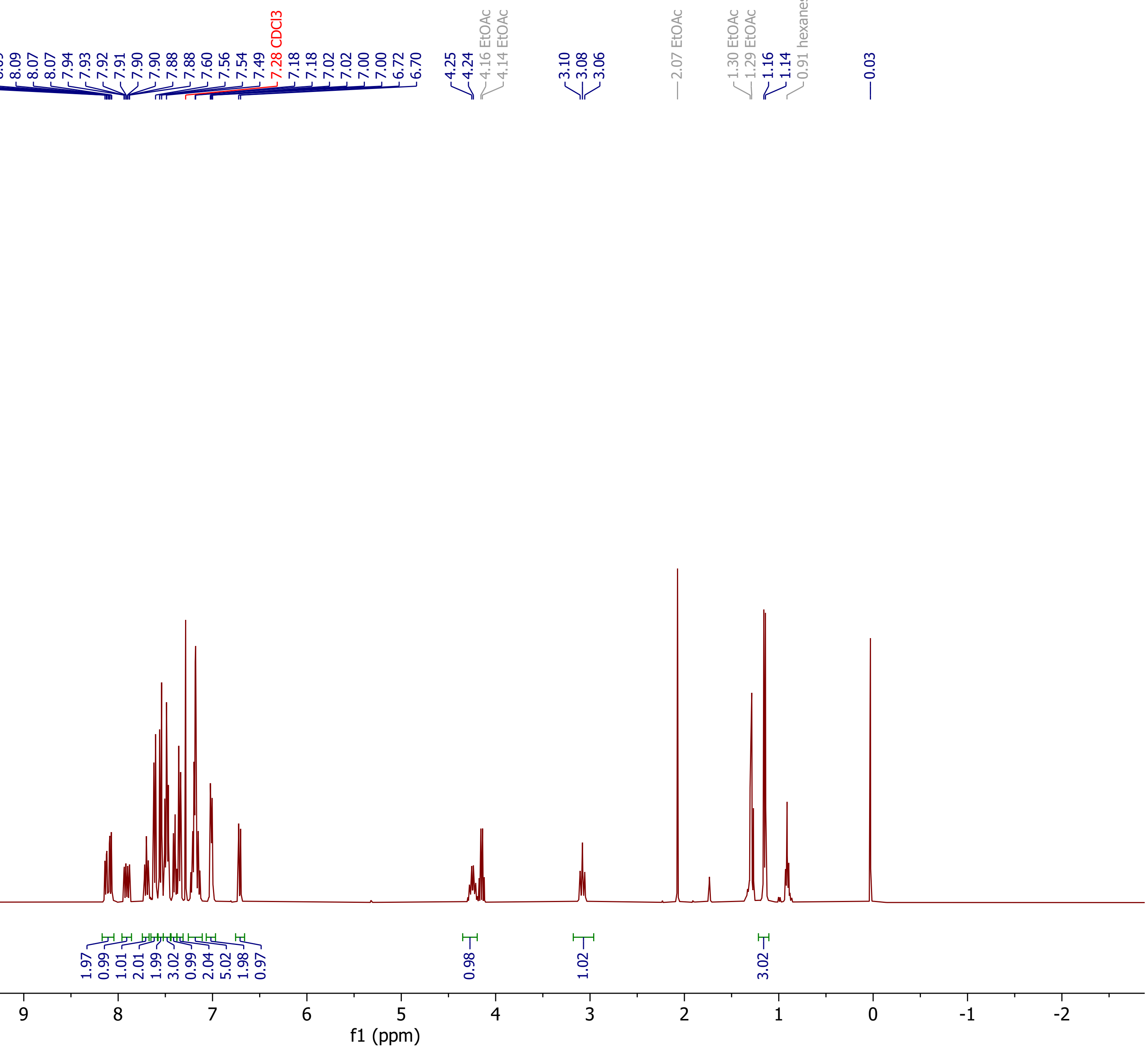

8.14
8.13
8.12
8.10
8.09
8.09
8.07
8.07
7.94
7.93
7.92
7.91
7.90
7.90
7.88
7.88
7.60
7.56
7.54
7.49
7.28 CDCl3
7.18
7.18
7.02
7.02
7.00
7.00
6.72
6.70
4.25
4.24
4.16 EtOAc
4.14 EtOAc
3.10
3.08
3.06
2.07 EtOAc
1.30 EtOAc
1.29 EtOAc
1.16
1.14
0.91 hexanes
0.03
1.97
0.99
1.01
2.01
1.99
3.02
0.99
2.04
5.02
1.98
0.97
0.98
1.02
3.02
13
12
11
10
9
8
7
6
5
4
3
2
1
0
-1
-2
f1 (ppm)

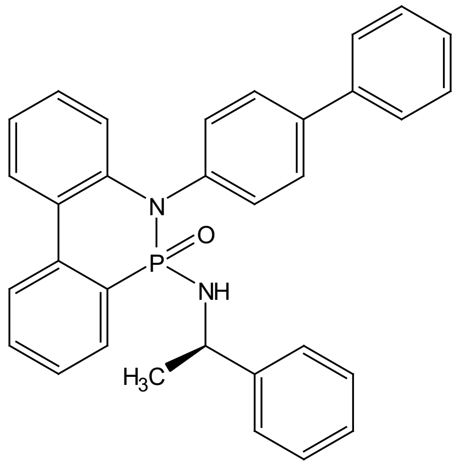
N
P
O
NH
H3C

17

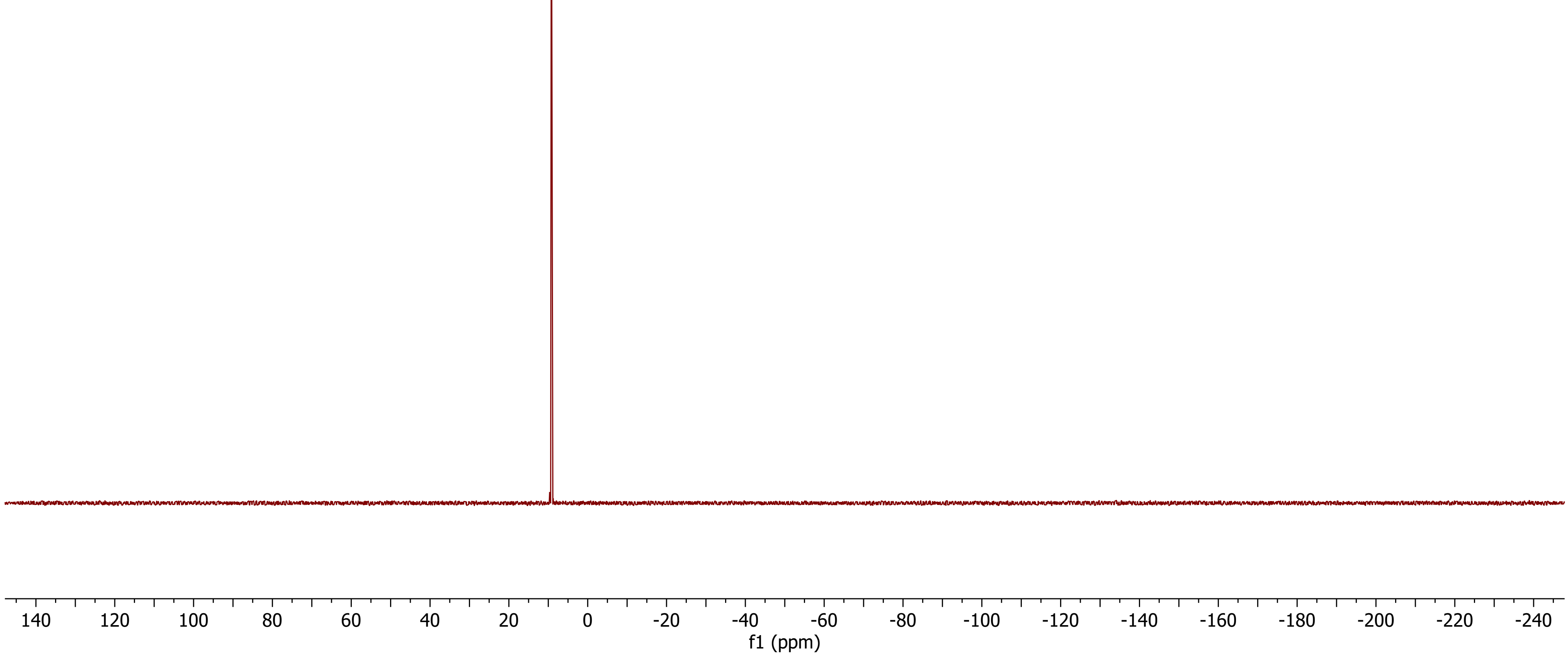
9.23
140 120 100 80 60 40 20 0 -20 -40 -60 -80 -100 -120 -140 -160 -180 -200 -220 -240
f1 (ppm)

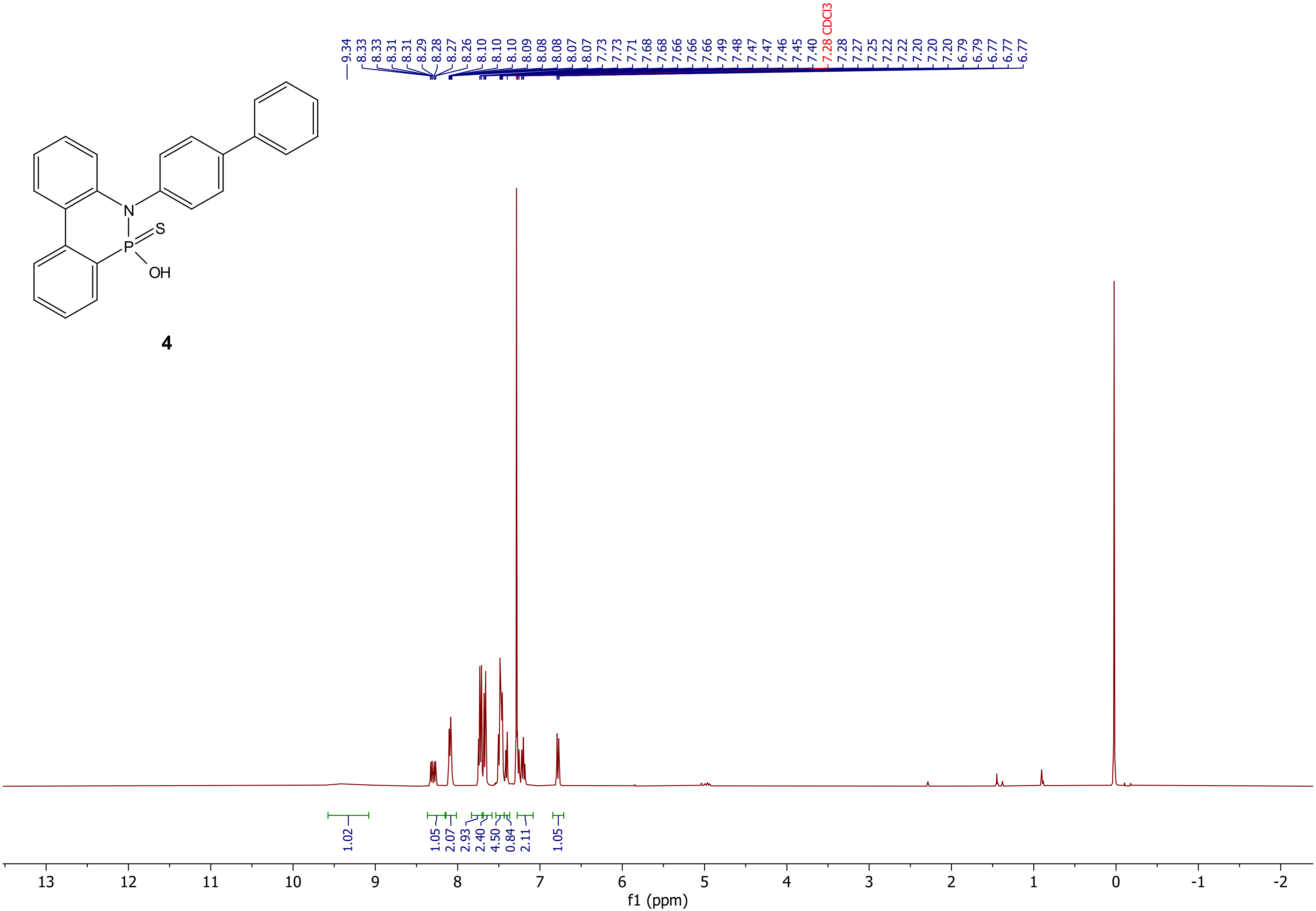
9.34
8.33
8.33
8.31
8.31
8.29
8.28
8.27
8.26
8.10
8.10
8.10
8.09
8.08
8.08
8.07
8.07
7.73
7.73
7.71
7.68
7.68
7.66
7.66
7.66
7.49
7.48
7.47
7.47
7.46
7.45
7.40
7.28 CDCl3
7.28
7.27
7.25
7.22
7.22
7.20
7.20
7.20
6.79
6.79
6.77
6.77
6.77
N
S
P
OH
4
1.02
1.05
2.07
2.93
2.40
4.50
0.84
2.11
1.05
13
12
11
10
9
8
7
6
5
4
3
2
1
0
-1
-2
f1 (ppm)

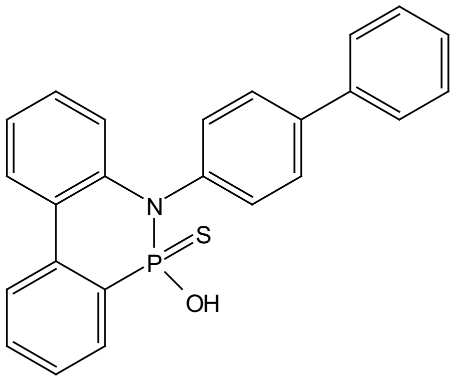
N
S
P
OH

4

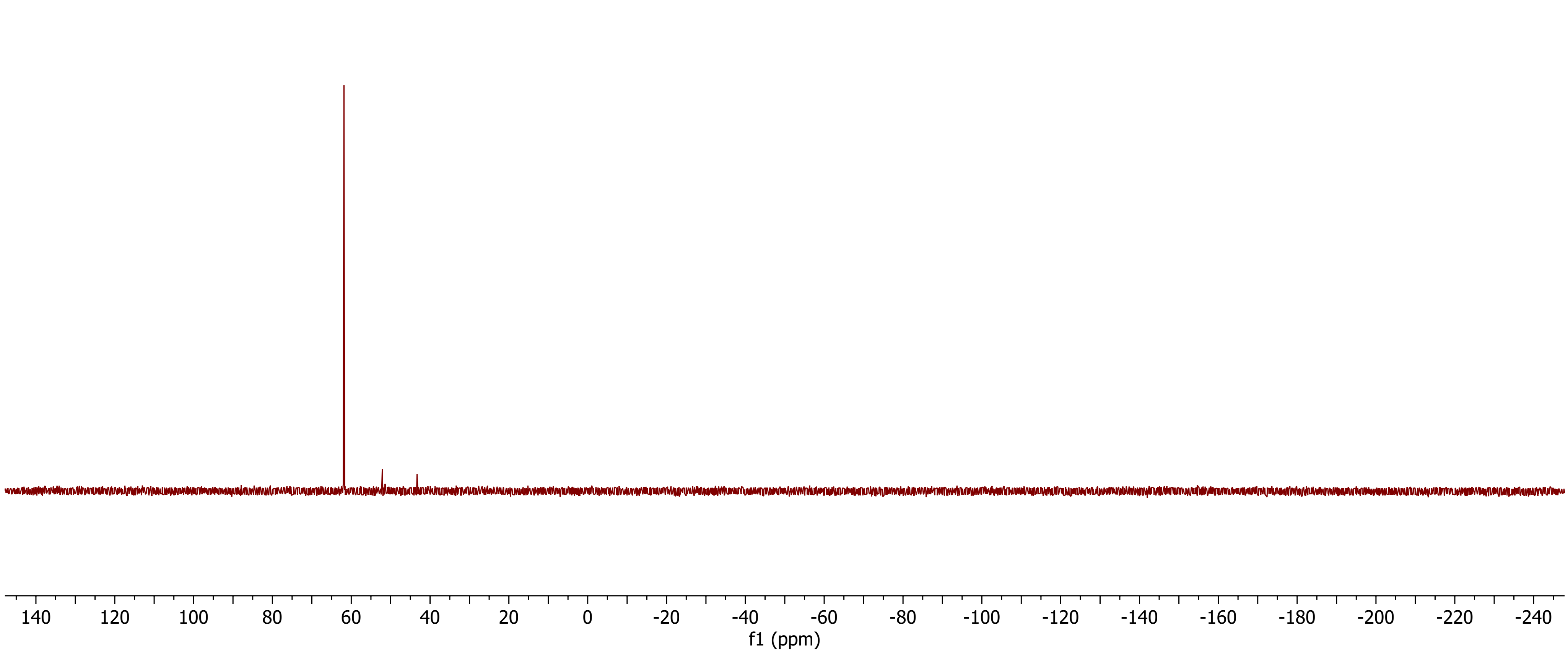
61.84
140
120
100
80
60
40
20
0
-20
-40
-60
-80
-100
-120
-140
-160
-180
-200
-220
-240
f1 (ppm)
